# Identification of diphenylurea derivatives as novel endocytosis inhibitors that demonstrate broad-spectrum activity against SARS-CoV-2 and influenza A virus both *in vitro* and *in vivo*

**DOI:** 10.1371/journal.ppat.1011358

**Published:** 2023-05-01

**Authors:** Nirmal Kumar, Irshad Maajid Taily, Charandeep Singh, Sahil Kumar, Raju S. Rajmani, Debajyoti Chakraborty, Anshul Sharma, Priyanka Singh, Krishan Gopal Thakur, Raghavan Varadarajan, Rajesh P. Ringe, Prabal Banerjee, Indranil Banerjee

**Affiliations:** 1 Cellular Virology Lab, Department of Biological Sciences, Indian Institute of Science Education and Research, Mohali (IISER Mohali), Mohali, India; 2 Department of Chemistry, Indian Institute of Technology Ropar (IIT Ropar), Rupnagar, Punjab, India; 3 Institute of Microbial Technology, Council of Scientific and Industrial Research (CSIR-IMTECH), Chandigarh, India; 4 Molecular Biophysics Unit, Indian Institute of Science, Bangalore (IISc), Bengaluru, India; Rutgers University: Rutgers The State University of New Jersey, UNITED STATES

## Abstract

Rapid evolution of severe acute respiratory syndrome coronavirus 2 (SARS-CoV-2) and influenza A virus (IAV) poses enormous challenge in the development of broad-spectrum antivirals that are effective against the existing and emerging viral strains. Virus entry through endocytosis represents an attractive target for drug development, as inhibition of this early infection step should block downstream infection processes, and potentially inhibit viruses sharing the same entry route. In this study, we report the identification of 1,3-diphenylurea (DPU) derivatives (DPUDs) as a new class of endocytosis inhibitors, which broadly restricted entry and replication of several SARS-CoV-2 and IAV strains. Importantly, the DPUDs did not induce any significant cytotoxicity at concentrations effective against the viral infections. Examining the uptake of cargoes specific to different endocytic pathways, we found that DPUDs majorly affected clathrin-mediated endocytosis, which both SARS-CoV-2 and IAV utilize for cellular entry. In the DPUD-treated cells, although virus binding on the cell surface was unaffected, internalization of both the viruses was drastically reduced. Since compounds similar to the DPUDs were previously reported to transport anions including chloride (Cl^-^) across lipid membrane and since intracellular Cl^-^ concentration plays a critical role in regulating vesicular trafficking, we hypothesized that the observed defect in endocytosis by the DPUDs could be due to altered Cl^-^ gradient across the cell membrane. Using *in vitro* assays we demonstrated that the DPUDs transported Cl^-^ into the cell and led to intracellular Cl^-^ accumulation, which possibly affected the endocytic machinery by perturbing intracellular Cl^-^ homeostasis. Finally, we tested the DPUDs in mice challenged with IAV and mouse-adapted SARS-CoV-2 (MA 10). Treatment of the infected mice with the DPUDs led to remarkable body weight recovery, improved survival and significantly reduced lung viral load, highlighting their potential for development as broad-spectrum antivirals.

## Author summary

Many pathogenic viruses including SARS-CoV-2 and IAV exploit endocytic pathways to initiate internalization into the host cell, and therefore, inhibiting these pathways with small molecules should effectively restrict viruses at the initial phase of infection. However, cytotoxicity represents one of the major obstacles in the therapeutic use of endocytosis inhibitors, as disruption of endocytosis may severely impact cell health. In this study, we identified DPUDs as a novel class of endocytosis inhibitors, which robustly blocked several IAV strains and SARS-CoV-2 variants of concern (VOC) in tissue culture cells. Remarkably, the DPUDs did not display any significant cytotoxicity at concentrations effective against the viruses. Our data indicate, among different endocytic pathways, clathrin-mediated endocytosis was most sensitive to DPUD treatment. We found that the DPUDs transported chloride ions into the cell, and disrupted endocytic trafficking possibly by perturbing intracellular chloride homeostasis. Confirming the antiviral activity of DPUDs in tissue culture cells, we evaluated their therapeutic potential in animal models of SARS-CoV-2 and influenza infections. SARS-CoV-2- or IAV-infected mice that received DPUD treatment displayed remarkable improvement in survival and clinical features as compared to the control animals, indicating their potential to be developed as highly-effective, broad-spectrum antivirals.

## Introduction

Targeting endocytic pathways that many viruses exploit to enter the host cells represents an attractive strategy for antiviral development, as deterring viruses at their entry portals should block the subsequent steps of virus life cycle [1]. Since many viruses share common endocytic pathways for cellular entry, pharmacological agents targeting endocytosis should broadly inhibit viruses at the initial phase of infection. Moreover, such agents are unlikely to induce resistance as they target cellular factors or processes rather than the virus-encoded proteins that often acquire escape mutations, rendering the virus-targeted antivirals ineffective [2]. Most of the anti-SARS-CoV-2 and anti-IAV drugs currently on the market are direct-acting antivirals, and the rapid emergence of viral strains with mutations capable of subverting the action of drugs represents a major concern. Recent studies provided evidence of the emergence of resistance mutation in SARS-CoV-2 against remdesivir, a drug that targets the viral RNA-dependent RNA polymerase nsp12, and is widely used in the treatment of hospitalized patients with coronavirus disease (COVID-19) [3,4]. SARS-CoV-2 variants with mutations conferring resistance against the approved drug nirmatrelvir, an inhibitor of the viral protease M^Pro^, have also been reported [5]. Similarly, several resistance mutations in IAV against the approved drugs targeting the viral protein neuraminidase have been detected [6]. The emergence of drug-resistant viral strains may potentially impart adverse consequences for therapeutic interventions, and underscores the need for urgently identifying novel compounds with higher barrier to resistance than the direct-acting, canonical antivirals. Complementing traditional approaches directed at virus-encoded targets, host-directed antiviral strategies are viable options to effectively attenuate the existing and emerging viruses. Among several possibilities in host-directed interventions, targeting virus entry by perturbing endocytic pathways offers exciting potential for broad inhibition of a wide range of viruses.

In the viral entry program, the viruses that do not fuse at the plasma membrane depend on endocytic pathways to enter the host cells. Compounds that directly affect the functions of the endocytic machinery or interfere with the signalling cascades that activate endocytic uptake of viruses can potentially block virus entry [2]. SARS-CoV-2 entry is initiated by the engagement of the viral spike (S) glycoprotein with the ACE2 receptor present at the plasma membrane [7]. In addition to ACE2, several other molecules have been uncovered, which have been suggested to serve as alternate receptors or co-receptors. These include Neuropilin-1 [8,9], C-type lectins, DC-SIGN and L-SIGN [10], CD147 [11], heparan sulfate [12], and HDL-scavenger receptor B type 1 [13]. Upon binding to the ACE2 receptor, SARS-CoV-2 either fuses at the plasma membrane through activation of the S glycoprotein by the transmembrane serine protease TMPRSS2 [14], or the virus is internalized via endocytosis and fuses at the endosomal membrane, following S glycoprotein activation by the endosomal cathepsins [15,16]. Although the virus prefers activation of the S glycoprotein by TMPRSS2 and fusion at the plasma membrane, it enters via endocytosis if the cell is TMPRSS2 deficient or if the virus fails to encounter TMPRSS2 at the plasma membrane [17]. Among the endocytic routes of entry, SARS-CoV-2 uses either clathrin-mediated endocytosis (CME) [18] or the clathrin-independent carriers/ GPI-anchored protein-enriched early endosomal compartment (CLIC/GEEC) endocytic pathway [19]. In the host cell entry of human IAV, the virus particle first binds to the α2,6-linked sialic acid on the cell surface via its hemagglutinin (HA) glycoprotein. IAV engages different internalization receptors including the epidermal growth factor receptor (EGFR) [20], voltage-dependent calcium channel Cav1.2 [21], and nucleolin [22]. Recent studies indicated that IAV entry can also occur by sialoglycan-independent mechanisms [23]. Upon engaging with the surface receptors, IAV enters the host cell via either CME or macropinocytosis [23,24]. While CME is utilized by several viruses including orthomyxoviruses, coronaviruses, flaviviruses, alphaviruses, and rhabdoviruses [18,25], larger viruses such as poxviruses and filoviruses that cannot fit into the clathrin-coated vesicles use macropinocytosis for cellular entry [2,26–28].

Since many viruses depend on endocytosis to gain access to the cell interior for replication, inhibition of endocytic pathways represents a promising antiviral strategy to prevent viral infections. Despite several advantages, clinical application of host-directed interventions has been limited due to potential toxicity, and finding agents with potent antiviral activity but with minimal or tolerable toxicity, has remained a major challenge in antiviral development. In this study, we have identified 1,3-diphenylurea (DPU) derivatives (DPUDs) as a new class of potential antivirals that robustly inhibited SARS-CoV-2 and IAV infections both *in vitro* and *in vivo*, without inducing toxicity. The hit compounds that we identified through high-content screening against the viruses included the DPUDs with electron-withdrawing trifluoromethyl groups at the *meta* positions (DPUD-1), *para* positions (DPUD-2), fluoro groups at the *meta* positions (DPUD-16), trifluoromethoxy groups at the *para* positions (DPUD-20), and bromo groups at the *meta* positions (DPUD-23). Since the hit DPUDs restricted both the viruses, we reasoned that they might target common pathway(s) critical for infection. We examined the host cell entry of the viruses using fluorescently-labelled and pseudotyped SARS-CoV-2, and indirect immunofluorescence (IIF)-based IAV entry assays that we previously developed [29]. We found that DPUD treatment significantly reduced the entry of both SARS-CoV-2 and IAV. However, virus entry was unperturbed in cells treated with the unsubstituted compound i.e. DPU, highlighting the importance of the above electron-withdrawing functional groups in virus entry inhibition. Compounds similar to DPUDs with electron-withdrawing groups were reported to transport chloride (Cl^-^) across lipid membrane [30], and changes in subcellular Cl^-^ concentration and transport were found to affect vesicular trafficking and endosomal acidification [31]. Using large unilamellar vesicle (LUV)-based *in vitro* Cl^-^ transport assay and by expressing a genetically-encoded, yellow fluorescent protein (YFP) Cl^-^ sensor in cells, we found that DPUDs transported Cl^-^ across lipid membrane and led to intracellular Cl^-^ accumulation, which possibly attenuated virus trafficking into the cells. To test whether the DPUDs affect endocytic pathways in general, we monitored the uptake of different endocytic cargoes. Treatment with the DPUDs significantly reduced both receptor-mediated endocytosis and macropinocytosis, whereas no effect was observed in cells treated with DPU. Taken together, our data suggest that DPUDs inhibit virus infection by affecting endocytic trafficking, possibly through perturbation of intracellular Cl^-^ homeostasis. Finally, we tested the efficacy of two DPUDs (DPUD-1 and -23) in mice challenged with SARS-CoV-2, and found that treatment with the DPUDs led to remarkable body weight recovery, significantly reduced lung viral load, and provided complete protection against lethality.

## Results

### Antiviral screenings identify DPUDs as inhibitors of IAV and SARS-CoV-2 infection

**Fig 1:**
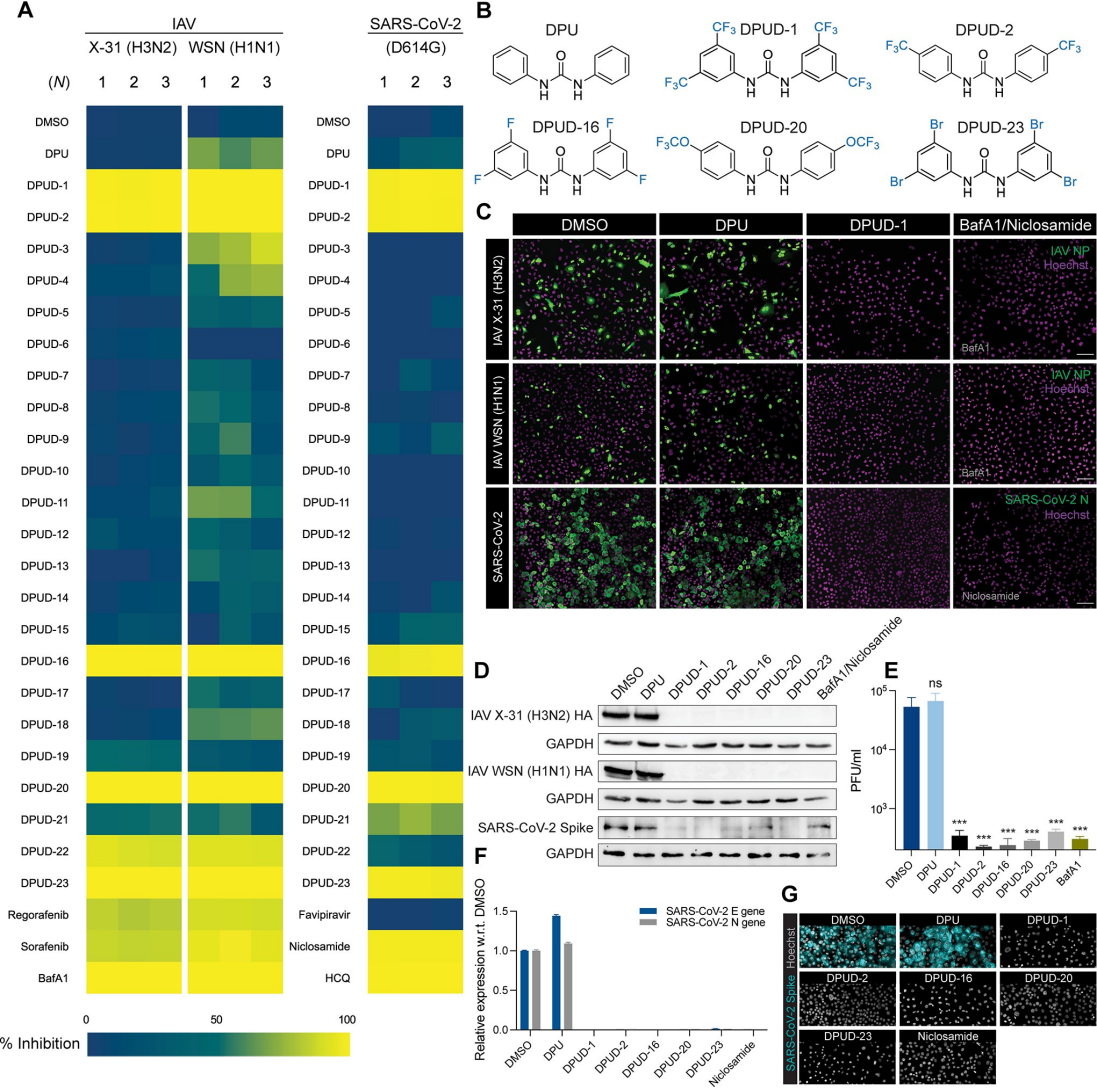
DPUDs inhibit IAV and SARS-CoV-2 infections in tissue culture cells. **(A)** Heatmaps of DPUD (10 *μ*M) screens against IAV X-31 (H3N2), WSN (H1N1), and SARS-CoV-2 (D614G). **(B)** Molecular structures of DPU and ‘hit’ compounds (DPUD-1, -2, -16, -20, and -23) that blocked IAV and SARS-CoV-2 infections by 95–100%. **(C)** High-content microscopy images of IAV- and SARS-CoV-2-infected cells. Nuclei were stained with Hoechst (magenta), and the viral NP/N proteins (green) were detected by IIF. Scare bars, 50 *μ*m. **(D)** Western blots showing inhibition of IAV (X-31 and WSN strains) and SARS-CoV-2 (D614G) infections by DPUDs (10 *μ*M) in A549 and Vero-E6 cells, respectively. IAV HA and SARS-CoV-2 Spike protein were detected using anti-HA antibody and anti-S antibody, respectively. GAPDH served as a loading control. **(E)** Results of viral plaque assay from the supernatants of A549 cells infected with IAV WSN strain. **(F)** Relative expression of SARS-CoV-2 N and E genes, detected by RT-PCR, in the supernatants of infected cells. **(G)** High-content microscopy images SARS-CoV-2-infected cells. Nuclei were stained with Hoechst (grey), and the viral Spike protein (cyan) was detected by IIF. All data are represented as mean ± SD. The *P*-value was determined using one-way ANOVA with multiple comparisons w.r.t. DMSO. ns: *P* >0.05, *P <0.05, **P <0.01, ***P <0.001, ****P <0.0001.

In an initial high-content small molecule screen with 28 compounds (10 *μ*M), we identified a DPUD (DPUD-1) that blocked IAV infection by 99.8% in human alveolar epithelial cells (A549), without inducing any apparent cytotoxicity (**S1 Table**). Encouraged by the potent antiviral activity of the compound, we synthesized twenty two additional DPUDs (**S2 Table**) by derivatising the starting compound i.e. DPU. The derivatized DPUDs were characterized by nuclear magnetic resonance (NMR) spectroscopy (**S3 Table**), and the purity of the compounds was assessed by high-performance liquid chromatography (HPLC). The purity of all the DPUDs was found to be >99% as determined by HPLC (**S4 Table**). After characterization and purity check, we conducted high-content screening of the DPUDs (DPUD-1 to -23) at 10 *μ*M concentration against two IAV strains (A/X-31, H3N2 and A/WSN/1933, H1N1) and the SARS-CoV-2 Wuhan strain with the D614G mutation in the S glycoprotein (hereafter called the D614G strain) using cell-based infection assays. A549 cells and African green monkey kidney cells (Vero-E6) were infected with IAV and SARS-CoV-2, respectively, and the infected cells were detected by indirect immunofluorescence (IIF) using antibodies against the nucleoproteins of IAV (NP) and SARS-CoV-2 (N). Infection index was calculated by dividing the NP- or N-expressing cells with the total number of cells (**S1A Fig**). In the screen against SARS-CoV-2 infection, we used favipiravir, niclosamide and hydroxychloroquine (HCQ) as positive controls as these compounds were previously reported to inhibit SARS-CoV-2 [32]. In the screens against IAV infection, we included regorafenib and sorafenib, two urea-based kinase inhibitors that block virus fusion [33], and bafilomycin A1 (BafA1), a highly potent and selective inhibitor of vacuolar H^+^-ATPases (vATPase) that inhibits infection by blocking endosomal acidification [29]. Since the DPUDs are not soluble in water, but soluble in dimethyl sulfoxide (DMSO), we included DMSO as a negative control. Among all the tested compounds, DPUD-1, -2, -16, -20 and -23 blocked IAV and SARS-CoV-2 infections by 95–100% (**Figs 1A, 1C, and S1B**). However, DPU did not inhibit any of the viruses, and we could not detect any block in SARS-CoV-2 N protein expression by favipiravir. Notably, the heatmaps of the screens showed strikingly similar patterns of inhibition by the hit DPUDs against both the viruses (**Fig 1A**).

After screening the DPUDs against IAV and SARS-CoV-2 infections, we confirmed the efficacy of the hit DPUDs against the viral infections by western blotting, viral plaque assay, RT-PCR, and confocal imaging. Western blotting against IAV HA and SARS-CoV-2 S glycoprotein revealed marked decrease in the viral protein levels in the DPUD-treated cells as compared to the cells treated with either DMSO or DPU (**Fig 1D**). To examine the effect of the DPUDs in the production of infectious virions, we performed IAV (WSN) plaque assay in Madin-Darby canine kidney (MDCK) cells. Treatment with the DPUDs significantly reduced IAV titer as compared to DMSO or DPU controls, indicating that a block at the early stage of viral infection as evidenced by the inhibition of viral protein expression, consequently led to a reduction in viral progeny production (**Fig 1E**). We also checked SARS-CoV-2 viral titer from the supernatant of infected cells by reverse transcription-polymerase chain reaction (RT-PCR) targeting the viral E and N genes. We found that treatment with the hit DPUDs (10 *μ*M) decreased viral titres by 99–100% as compared to the DMSO or DPU controls (**Fig 1F**). Confocal imaging of SARS-CoV-2-infected cells using IIF against the viral S glycoprotein further confirmed that while DMSO- or DPU-treated cells were highly susceptible to infection, treatment with the DPUDs (10 *μ*M) robustly blocked the expression of S glycoprotein (**Fig 1G**). Next, we examined the inhibitory potency of the DPUDs (10 *μ*M) against IAV infection in MDCK and HeLa cells, in addition to A549 cells. We infected MDCK and HeLa cells with IAV (X-31) in presence of the DPUDs, and found that all the compounds blocked infection by 90–99% in MDCK cells. Although DPUD-20 reduced IAV infection by 56% in HeLa cells, the other DPUDs blocked infection by >99% (**S1C Fig**). The results of IAV infection in MDCK and HeLa cells suggested that the potency of the DPUDs is not restricted to any particular cell line; the majority of the hit DPUDs can be effective against IAV infection across different cell lines.

We so far evaluated the level of attenuation of SARS-CoV-2 infection by the DPUDs in Vero-E6 cells which express the ACE2 receptor, but lack TMPRSS2 that facilitates viral fusion at the plasma membrane by cleaving the S glycoprotein at the S1/S2 junction and activating the S2 domain for fusion [14,34]. In the absence of cell surface TMPRSS2 in the Vero-E6 cells, SARS-CoV-2 majorly utilizes endocytic pathways for cellular entry and depends on endosomal cathepsins for activation of the S glycoprotein for fusion [35–37]. To examine the anti-SARS-CoV-2 potency of the DPUDs in cells that express TMPRSS2 at the cell surface in addition to ACE2, we included Vero-E6 cells stably expressing TMPRSS2 (Vero-E6-TMPRSS2). We pre-treated Vero-E6-TMPRSS2 cells with the DPUDs (10 *μ*M) or BafA1 (50 nM) or niclosamide (10 *μ*M) in presence/absence of the TMPRSS2 inhibitor camostat [38] (50 *μ*M) for 1 h, following which we infected the cells with SARS-CoV-2 (D614G) with the DPUDs/BafA1/niclosamide at the indicated concentrations, both in presence/absence of camostat (50 *μ*M). We fixed the cells at 24 hours post-infection (h.p.i.), and performed IIF against the viral N protein to detect the infected cells. We found that irrespective of camostat treatment, the DPUDs, BafA1, and niclosamide blocked SARS-CoV-2 infection in the Vero-E6-TMPRSS2 cells by 91–100% (**S1D Fig**). Taken together, the above results suggest that DPUDs can robustly block SARS-CoV-2 infection both in absence or presence of active/ pharmacologically inhibited TMPRSS2.

### DPUDs exhibit high selectivity indices and display broad-spectrum activity against different strains of IAV and SARS-CoV-2

**Fig 2:**
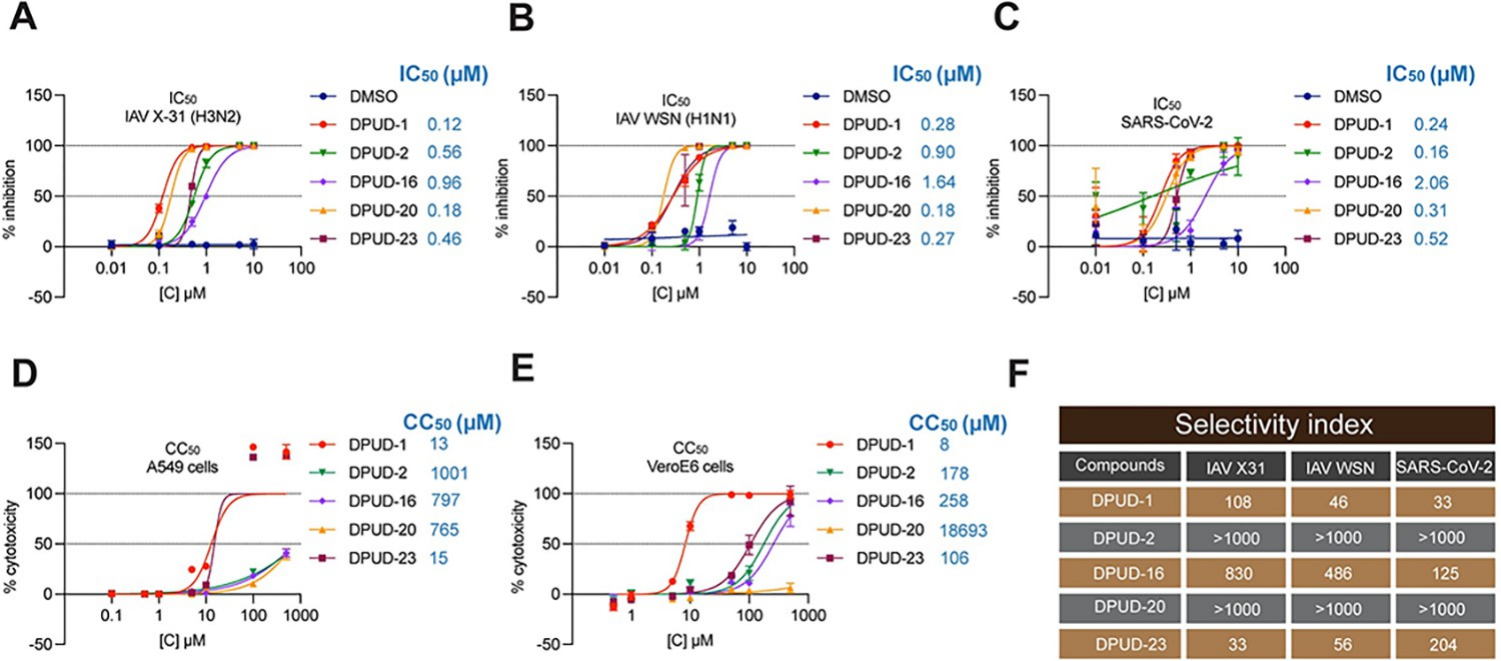
DPUDs exhibit high selectivity indices against IAV and SARS-CoV-2 infection. **(A-C)** Graphs showing concentration-dependent effect of DPUDs against IAV (X-31 and WSN) and SARS-CoV-2 (D614G) infections in A549 and Vero-E6 cells, respectively. The half-maximal inhibitory concentration (IC_50_) values corresponding to each compound are shown. **(D-E)** Cytotoxicity assessment DPUDs in A549 and Vero-E6 cells. The cells were treated with different concentrations of DPUDs for 24 h, following which, LDH cytotoxicity assay was performed. The 50% cytotoxic concentration (CC_50_) values corresponding to each compound are shown. **(F)** Selectivity indices of the compounds for IAV X-31 and WSN, and SARS-CoV-2 (D614G) infections.

After identifying the hit DPUDs and checking their efficacy against the IAV WSN, X-31 and SARS-CoV-2 D614G strains, we determined the half maximal inhibitory concentration (IC_50_) of the compounds (DPUD-1, -2, -16, -20 and -23) against IAV and SARS-CoV-2 in A549 and Vero-E6 cells, respectively. The hit DPUDs were serially diluted from 10 *μ*M to 10 nM, and the cells were pre-treated with respective dilutions of the compounds in infection medium at 37°C for 1 h. Following pre-treatment, the cells were infected with the viruses (IAV in A549 cells and SARS-CoV-2 in Vero-E6 cells) in presence of different concentrations of DPUDs, and after infection, the cells were incubated at 37°C for 10 h (IAV) or 24 h (SARS-CoV-2) in a CO_2_ incubator. After incubating the cells for appropriate time periods to allow the expression of viral proteins (NP for IAV and N for SARS-CoV-2), we performed IIF to detect the infected cells. Next, we determined the IC_50_ values using non-linear regression function and plotted the DPUDs vs. normalised response-variable slope. We found that except DPUD-16, all the other compounds showed IC_50_ values <1.0 *μ*M for both IAV and SARS-CoV-2. The IC_50_ for IAV (H1N1) and SARS-CoV-2 (D614G) were 1.64 *μ*M and 2.06 *μ*M, respectively (**Fig 2A–2C**). Next, we determined the 50% cytotoxic concentration (CC_50_) of the DPUDs in A549 and Vero-E6 cells (**Fig 2D and 2E**) by colorimetric measurement of lactate dehydrogenase (LDH) released in the culture medium from the treated cells. A549 or Vero-E6 cells were treated with the DPUDs serially diluted from 500 *μ*M to 1 *μ*M in the cell culture medium, and the released LDH was measured 24 h post-treatment from the supernatant to calculate the CC_50_ values. After determining the IC_50_ and CC_50_ values, we calculated the selectivity index (SI), which reflects the suitability of a compound for evaluation of its potential for therapeutic application. In general, an SI value ≥10 of any compound is considered suitable for further investigation. We found that all the hit DPUDs had SI values >30, and DPUD-2 and -20 had SI values >1000, indicating the suitability of all the hit compounds for further evaluation as potential antivirals (**Fig 2F**). To check the breath of the inhibitory effect of the hit DPUDs, we tested them against two additional strains of IAV (A/New York/55/2004 (NYMC), H3N2, and A/Udorn/1972 (Udorn), H3N2) and the SARS-CoV-2 variants of concern (VOC) (Delta, B.1.617.2, Omicron, B.1.1.529, and Omicron BA.5). We found that while DPUD-16 and DPUD-20 reduced IAV NYMC and Udorn infections by 52–97%, DPUD-1, -2, and -23 inhibited both the viruses by almost 100% (**S2A and S2B Fig**). In SARS-CoV-2 infections with the VOC, DPUD-20 did not show any significant inhibitory potency. Although DPUD-16 was ineffective against SARS-CoV-2 Delta, it significantly blocked the SARS-CoV-2 Omicron variants. However, DPUD-1, -2, and -23 reduced SARS-CoV-2 Delta infection by 87%, 75% and 85%, respectively. The same compounds blocked both the SARS-CoV-2 Omicron variants by 95–100% (**S2C–S2E Fig**). The above results suggest that among the hit DPUDs, DPUD-1, -2, and -23 can robustly attenuate multiple IAV and SARS-CoV-2 strains, indicating their broad-spectrum antiviral potency.

### DPUDs target cellular entry of IAV and SARS-CoV-2

**Fig 3:**
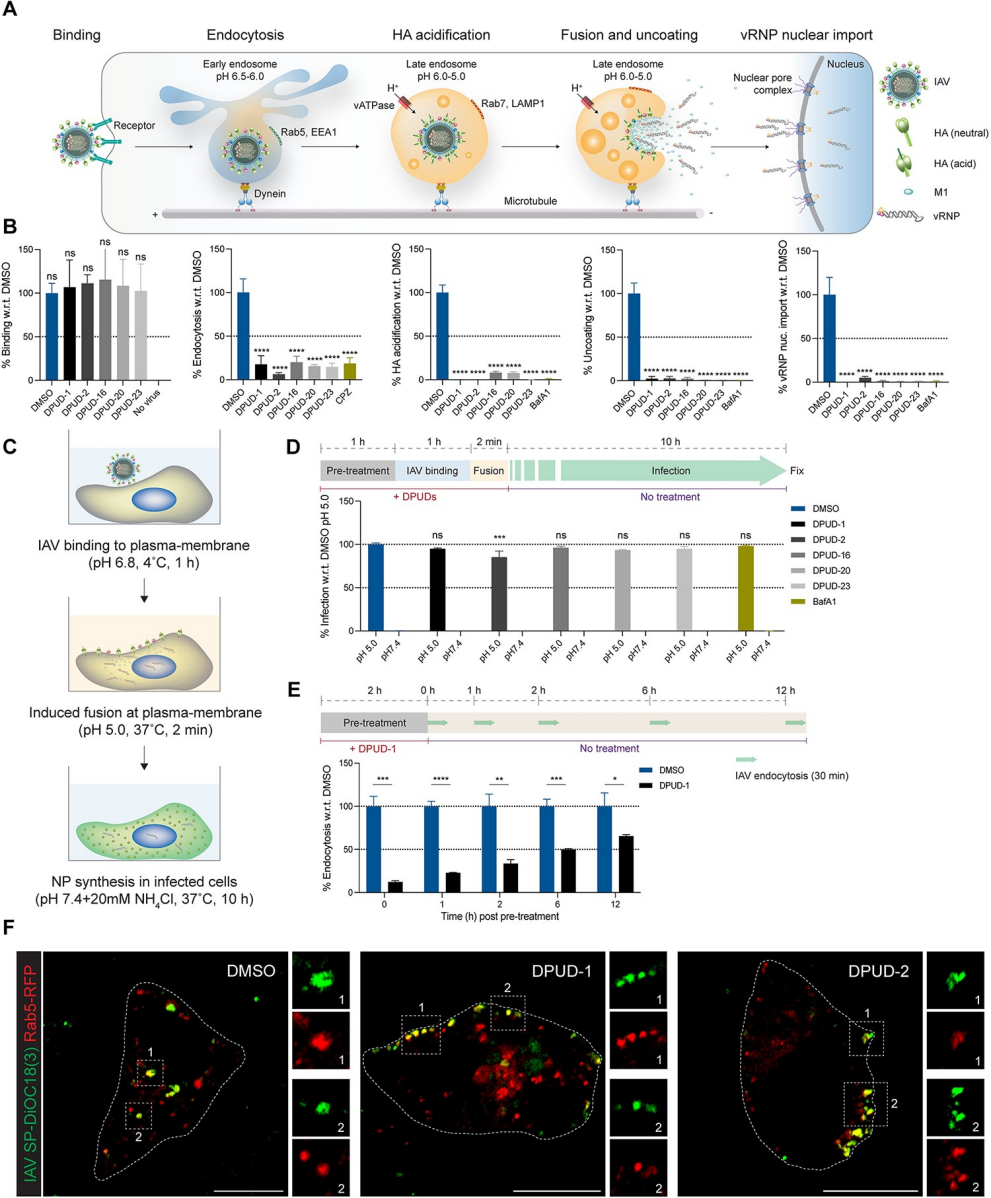
DPUDs block IAV endocytosis. **(A)** Schematic diagram showing the multi-step IAV entry process. **(B)** Effect of DPUDs (10 *μ*M) on IAV binding to PM, endocytosis, HA acidification in late endosomes, capsid uncoating, and vRNP nuclear import. **(C)** Schematic diagram showing the steps of PM bypass IAV infection assay. **(D)** Restoration of IAV infection by induced fusion of virus particles at PM in presence of DPUDs (10 *μ*M). **(E)** IAV endocytosis is blocked in A549 cells pre-treated with DPUD-1 (10 *μ*M). Cells were pre-treated with DPUD-1 (10 *μ*M) for 2 h, followed by removal of the compound. After pre-treatment, IAV endocytosis assay was performed at 0, 1, 2, 6, and 12 h. The *P*-value was determined using unpaired *t* test w.r.t. DMSO. ns: *P* >0.05, *P <0.05, **P <0.01, ***P <0.001, ****P <0.0001. **(F)** Images from live cell microscopy, monitoring IAV entry. IAV (X-31) particles were labelled with SP-DiOC18(3) (green) and were allowed to enter A549 cells expressing Rab5-RFP (red) in presence of DMSO or DPUD-1 or DPUD-2 (10 *μ*M) for 25–30 min, following which, images were acquired. Yellow signal indicates colocalization of the virus (green) and Rab5-positive early endosome (red). Cell boundary is shown with dotted line. Scare bars, 10 *μ*m. The *P*-value was determined using one-way ANOVA with multiple comparisons w.r.t. DMSO. ns: *P* >0.05, *P <0.05, **P <0.01, ***P <0.001, ****P <0.0001.

Next, we interrogated which step of virus infection cycle was targeted by the hit DPUDs. We first examined the effect of the DPUDs on IAV entry using our previously developed quantitative assays to monitor IAV at different stages of infection, including cellular entry [29]. Cellular entry of IAV is a multi-step process (**Fig 3A**), which begins with binding of the virus to the sialic acid-containing glycoproteins on the plasma membrane, followed by internalization through clathrin-mediated endocytosis and macropinocytosis [23,39]. After sorting to acidic late endosomes, viral HA undergoes low pH-induced conformational change, facilitating fusion between the viral envelop and the endosomal membrane, which leads to capsid uncoating and release of viral ribonucleoproteins (vRNPs) into the cytosol [40]. The vRNPs are then imported into the nucleus for replication and transcription [41]. We pre-treated cells with the DPUDs (10 *μ*M) for 1 h, following which we performed the IAV entry assays using the X-31 strain in the presence of the compounds (10 *μ*M). Although we did not observe any significant difference in virus binding, virus endocytosis was blocked by ≥80% in cells treated with the DPUDs. The subsequent steps of IAV entry i.e. HA acidification, capsid uncoating and vRNP nuclear import were almost completely blocked (**Figs 3B and S3A**). To further confirm that DPUDs target IAV endocytosis, we induced virus fusion at the plasma membrane (PM) at pH 5.0, a process that allows direct delivery of vRNPs into the cytosol, bypassing the endocytic route of vRNP delivery [42]. As a control, we used a pH 7.4 medium with ammonium chloride (NH_4_Cl), which does not induce virus fusion at the PM. After inducing fusion of the virus particles at the PM at pH 5.0, the cells were shifted to a pH 7.4 medium in which we added NH_4_Cl to prevent infection by the unfused, endocytosed viruses (**Fig 3C**). After 10 h post-fusion, we performed IIF to detect NP in the infected cells. Treatment with the DPUDs (10 *μ*M) resulted in 80–98% infection as compared to the DMSO control (**Fig 3D**). Restoration of infection by induced viral fusion at the PM indicated that the DPUDs specifically target IAV endocytosis; if viral genome is directly delivered into the cytosol bypassing the endocytic entry routes, the DPUDs fail to attenuate infection. Next, we performed time of addition assay to check the effect of DPUD-1 pre-treatment on IAV endocytosis over time. We pre-treated A549 cells with DPUD-1 (10 *μ*M) for 2 h, following which we removed the compound, thoroughly washed the cells with infection medium to ensure complete removal of the compound, and added fresh infection medium to the cells. We performed IAV endocytosis assay for 30 min in the DPUD-1-pre-treated cells at 0 h, 1 h, 2 h, 6 h, and 12 h post DPUD-1 removal. Remarkably, we found that only 2 h pre-treatment of the cells with DPUD-1 was sufficient to reduce IAV endocytosis by 88% as compared to the DMSO control when the endocytosis assay was performed at 0 h post removal of the compound. The inhibitory effect of DPUD-1 pre-treatment on virus endocytosis progressively decreased over time, but even after 12 h post removal of the compound, virus endocytosis was reduced by 35% (**Fig 3E**). This suggests that DPUD-1 exerts prolonged inhibitory effect on the endocytic pathways exploited by IAV for host cell entry.

After confirming the DPUDs’ inhibitory effect on IAV endocytosis in fixed cells, we examined the effect of two compounds (DPUD-1 and DPUD-2) on virus entry in live cells. We overexpressed in A549 cells red fluorescent protein (RFP)-tagged Rab5, an early endosome marker, and to visualize IAVs, we labelled the virus particles with a fluorescent lipophilic dye, SP-DiOC18(3). We pre-treated the cells with DPUD-1 and DPUD-2 (10 *μ*M) for 1 h, following which, we added the fluorescently labelled virus particles to the cells in presence of the compounds. After allowing the viruses to internalize for 20–30 min at 37°C, we visualized them under a confocal microscope. We observed that although the virus particles could successfully internalize and reach the perinuclear region in the DMSO-treated cells, they failed to enter the DPUD-1- or DPUD-2-treated cells. In DMSO controls, the viruses displayed rapid and directed movements from the cell periphery towards the nuclei, often colocalizing with the Rab5-positive vesicles. However, upon DPUD-1 or DPUD-2 treatment, the majority of the virus particles remained stuck near the PM in clusters, colocalizing with a peripheral and relatively static pool of Rab5-positive vesicles (**Fig 3F**). Confirming the inhibitory effect of the DPUDs in IAV endocytosis, we addressed whether the compounds can also target SARS-CoV-2 entry. As a faithful model of SARS-CoV-2 entry, we used a luciferase-encoding and replication-defective human immunodeficiency virus-1 (HIV-1) [43], pseudotyped with the D614G, Delta and Omicron variants of the S protein. Since parental HEK293T cells were refractory to infection with the pseudotyped SARS-CoV-2 viruses, we used hACE2-expressing (HEK293T-hACE2) cells that were susceptible to infection with the pseudotyped viruses, as evidenced by luciferase expression in the infected cells. DPUD treatment in HEK293T-hACE2 cells robustly suppressed infection with all the pseudotyped SARS-CoV-2 variants, and we reasoned that the block in infection was due to virus entry inhibition as the pseudotyped viruses were replication-defective (**S3B–S3D Fig**). Also, we checked the entry of authentic SARS-CoV-2 (D614G) in the DPUD-1-treated Vero-E6 cells by allowing SP-DiOC18(3)-labelled virus particles internalize into the cells for 4 h. We found, DPUD-1 treatment robustly inhibited SARS-CoV-2 internalization (**S3E Fig**) Taken together, our results demonstrate that the DPUDs block cellular entry of both IAV and SARS-CoV-2.

### DPUDs target multiple endocytic pathways with most potent effect on clathrin-mediated endocytosis

**Fig 4:**
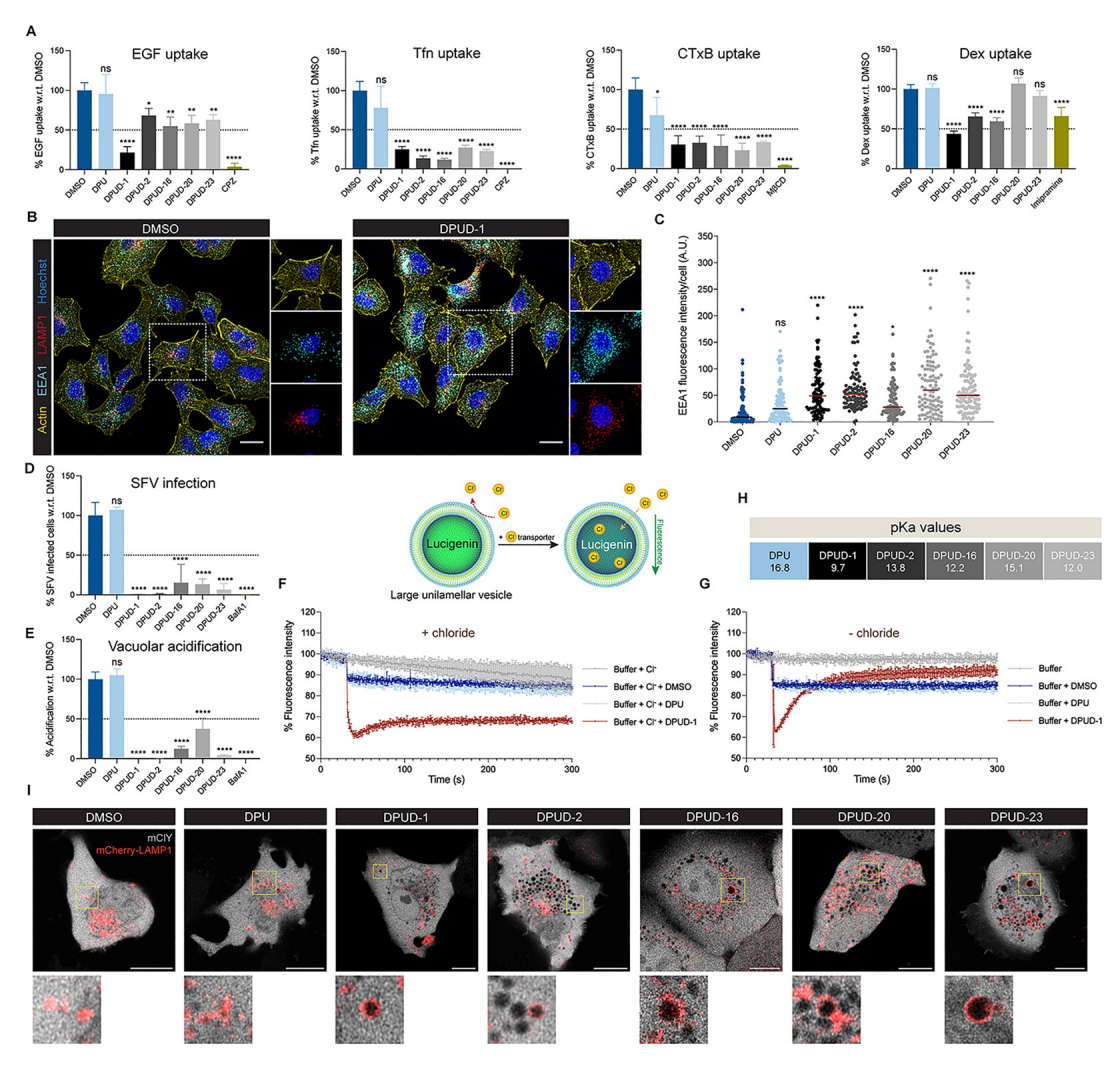
DPUDs target endocytosis and facilitate intracellular Cl^-^ transport. (**A**) Effect of DPUDs (10 *μ*M) on the cellular uptake of EGF, Tfn, CTxB, and Dex. **(B)** Confocal images of A549 cells treated with DPUD-1 (10 *μ*M) or DMSO for 1 h. IIF was performed to detect early and late endosomes using antibodies against EEA1 (cyan) and LAMP1 (red), respectively. Phalloidin-AF647 and Hoechst were used to stain actin filaments (yellow) and nuclei (magenta), respectively. Scale bars, 20 *μ*m. **(C)** Quantification of EEA1 fluorescence intensities in A549 cells treated with DPUDs (10 *μ*M) for 1 h. One hundred cells were counted for each compound. **(D)** Treatment with DPUDs (10 *μ*M) during entry phase (2 h) blocked SFV infection in BHK-21 cells. **(E)** Quantification of vacuolar acidification, probed by an acid-sensing dye LysoTracker Red DND-99. A549 cells were pre-treated with DPUDs (10 *μ*M) for 1 h, followed by incubation with the dye for 1 h in presence of the compounds. Vacuolar acidification was measured by quantifying the intensity of LysoTracker Red DND-99. **(F)** DPUD-1 facilitates Cl^-^ transport across lipid membrane. Large unilamellar vesicles (LUVs) containing Cl^—^sensitive lucigenin were generated. In presence of chloride ions, DMSO/DPU (10 *μ*M)/DPUD-1 (10 *μ*M) was added and the fluorescent intensity over time was measured. **(G)** Lucigenin fluorescence intensity in LUVs over time upon DMSO/DPU (10 *μ*M)/DPUD-1 (10 *μ*M) addition in absence of chloride ions. **(H)** Calculated pKa values of DPU and DPUDs. **(I)** Effect of DPUDs (10 *μ*M) in intracellular Cl- accumulation. A genetically-encoded YFP Cl^-^ sensor (mClY) (green) and mCherry-tagged LAMP1 (mCherry-LAMP1) (red) were expressed in A549 cells, and the cells were treated with DPUDs (10 *μ*M) for 1 h. Cell boundary is shown with dotted line. Dark regions in the zoomed images indicate Cl- accumulation. Scale bars, 10 *μ*m. The *P*-value was determined using one-way ANOVA with multiple comparisons w.r.t. DMSO. ns: *P* >0.05, *P <0.05, **P <0.01, ***P <0.001, ****P <0.0001.

To check whether IAV or SARS-CoV-2 entry inhibition by the DPUDs was virus-specific, or due to perturbation of endocytic pathways in general, we examined the cellular uptake of fluorescently-tagged epidermal growth factor (EGF), transferrin (Tfn), and cholera toxin B (CTxB), known to enter cells via clathrin-mediated and non-clathrin endocytic pathways [44,45], While DPU did not considerably affect the uptake of EGF, Tfn or CTxB, the DPUDs significantly blocked their internalization with most pronounced effect on Tfn uptake. We also examined the fluid-phase, macropinocytic uptake of fluorescently-tagged dextran (Dex), and found that DPUD-1, -2, and -16 significantly reduced Dex uptake (**Figs 4A and S4**). Confocal imaging of the DPUD-treated cells revealed significantly increased expression of EEA1, a marker of early endosome, and dispersed LAMP1-positive late endosome/lysosome (**Figs 4B, 4C, and S5A–S5F**). Since the clathrin-dependent pathway was most sensitive to the DPUDs among the endocytic pathways examined, we speculated that other viruses than IAV and SARS-CoV-2 that exclusively use this entry pathway would also be affected. To test this, we infected DPUD-treated BHK-21 cells with Semliki Forest virus (SFV), known to enter cell via clathrin-mediated endocytosis [46]. We pre-treated cells with the DPUDs for 2 h, and following pre-treatment, we infected them with SFV in presence of the compounds. The cells were treated with the DPUDs only during the initial 2 h of infection to check their effect on virus entry. We found that while DPU did not have any effect, the DPUDs robustly inhibited SFV infection (**Fig 4D**), indicating that the DPUDs can broadly inhibit viruses that enter cells through the pathways that the compounds target. Further, we checked endosomal acidification using LysoTracker, a fluorescent acidotropic probe that accumulates in acidic organelles. Treatment with DPUDs robustly inhibited organelle acidification with almost no LysoTracker-positive organelles detected in the cells treated with DPUD-1 or -2 (**Figs 4E and S4**). Taken together, the above results suggest that the DPUDs affect multiple endocytic pathways, which can potentially inhibit diverse viruses that avidly use these pathways for cell entry.

### DPUDs transport Cl^-^ across lipid membranes and lead to intracellular Cl^-^ accumulation

Compounds with electron-withdrawing functional groups and similar chemical properties as the DPUDs were previously reported to transport anions, including Cl^-^, across lipid membranes [30,47]. The presence of electron-withdrawing functional groups on the aromatic rings increases the acidity of the urea proton, which in turn facilitates hydrogen bonding with Cl^-^. Since Cl^-^ plays a critical role in membrane-trafficking and provides the main electrical shunt for endocytic organelle acidification [31], we hypothesized that the observed impairment of endocytosis by the DPUDs could be linked to dysregulated Cl^-^ transport. We, therefore, examined the effect of DPU and DPUD treatment on Cl^-^ transport across membranes using an *in vitro* assay with large unilamellar vesicles (LUVs) containing a chloride-sensitive fluorophore, lucigenin [48]. Addition of DPUDs caused a rapid drop in lucigenin fluorescence, indicating Cl^-^ influx into the LUVs. However, DPU addition did not lead to any substantial decrease in fluorescence (**Figs 4F, 4G, and S5G**). To verify that the drop in lucigenin signal was specific to the effect of the DPUDs on Cl^-^ transport activity, but not due to lucigenin leakage from disrupted LUVs, we performed calcein-release assay as previously described [49]. None of the compounds exhibited significant calcein release from the LUVs, indicating that LUV integrity was not impacted by DPUD treatment (**S5H Fig**). Since the *in vitro* experiments indicated that the DPUDs, but not DPU, can transport Cl^-^ across lipid membrane, we hypothesized that the substitutions on the phenyl rings of DPU impart charge differences on the molecules so that the affinity of the DPUDs for Cl^-^ increases. We, therefore, calculated the pKa values of DPU and the DPUDs using B3LYP/6-31G(d,p) to estimate the acid dissociation constants of the compounds and to check whether the values corroborate with our findings, especially with regard to the efficiency of the compounds in *in vitro* Cl^-^ transport and neutralization of luminal pH. While the calculated pKa value of DPU was 16.8, DPUD-1, -2, -16, -20 and -23 had pKa values of 9.7, 13.8, 12.2, 15.1, 12.0, respectively (**Fig 4H**). The lesser pKa values of the DPUDs compared to DPU indicated their higher acidity of the urea protons with increased affinity to Cl^-^ than DPU. Among the DPUDs, the pKa value of DPUD-20 was the highest, and the higher pKa value of DPUD-20 than the other DPUDs may account for its least Cl^-^ transport efficiency among the tested compounds (**S5G Fig**). Moreover, while DPUD-1, -2, -16 and 20-23 reduced endosomal acidification by 88–100%, only 67% decrease in acidification was observed in cells treated with DPUD-20 (**Fig 4E**). This indicates a possible correlation between the Cl^-^ transport efficiency of the DPUDs and the extent of luminal pH neutralization by them. Next, we addressed whether the DPUDs can transport Cl^-^ into cells. To test this, we expressed in A549 cells a genetically-encoded YFP Cl^-^ sensor (mClY), which responds rapidly and reversibly to changes in Cl^-^ concentration, with increased Cl^-^ concentration leading to a loss of fluorescence signal [50]. We also expressed mCherry-tagged LAMP1, a marker of late endosome/lysosome, in the cells to check whether Cl^-^ accumulates in endosomal compartments. DPUD treatment resulted in larger and higher number of YFP-negative vesicles compared to the cells treated with either DPU or DMSO, and LAMP1 signal was observed around many YFP-negative vesicles (**Fig 4I**). The above results indicate that DPUD treatment leads to dysfunction of endocytic trafficking, possibly by increasing intra-vesicular Cl^-^ accumulation and altering intracellular Cl^-^ homeostasis.

### DPUDs protect mice from lethal challenge with IAV or SARS-CoV-2

After confirming the antiviral potency of the DPUDs *in vitro*, we addressed whether they are effective against IAV infection in mice. First, we intranasally infected C57BL/6 mice with 10^3^ plaque-forming units (PFUs) of IAV (WSN). After 24 h, we intraperitoneally administered DPUD-1 at 1 mg/kg of body weight twice daily for 5 days. We included 6 mice per group, and used DMSO and oseltamivir acid as negative and positive controls, respectively (**S6A Fig**). Next, we measured the bodyweights of the mice for 10 days post-infection (d.p.i). We observed that DPUD-1 was effective in body weight recovery of the infected mice (**S6B Fig**). We also observed improved survival of the DPUD-1-treated mice in comparison to DMSO controls till 21 days post-infection (d.p.i.) (**S6C Fig**). Next, we determined the lung viral titres of the mice sacrificed on the sixth day post-infection by RT-PCR targeting the IAV NP gene and by virus plaque assay. We found significant reduction in lung viral titres in the DPUD-1-treated mice as compared to DMSO controls (**S6D and S6E Fig**). Also, in virus plaque assay we found significant reduction in IAV (WSN) titre upon DPUD-1 (1 *μ*M) treatment. The virus titre in the DPUD-1-treated cells was comparable to the titre in cells treated with oseltamivir acid (1 *μ*M) (**S6F Fig**). Next, we assessed the barrier of DPUD-1 to viral resistance. We passaged IAV (WSN) in MDCK cells in presence of DPUD-1 (1 *μ*M), oseltamivir acid (1 *μ*M) and DMSO for 10 passages. After the tenth passage, we assessed the DPUD-1- and oseltamivir-passaged viruses for their respective susceptibility and compared them with the DMSO-passaged viruses. While the oseltamivir-passaged viruses showed similar titre as the DMSO-passaged viruses, indicating that the viruses acquired resistance through serial passaging, the DPUD-1-passaged viruses showed >5 log reduction in viral titre (**S6G Fig**). This suggests that DPUD-1 has significantly higher barrier to resistance than the approved anti-influenza drug oseltamivir.

**Fig 5:**
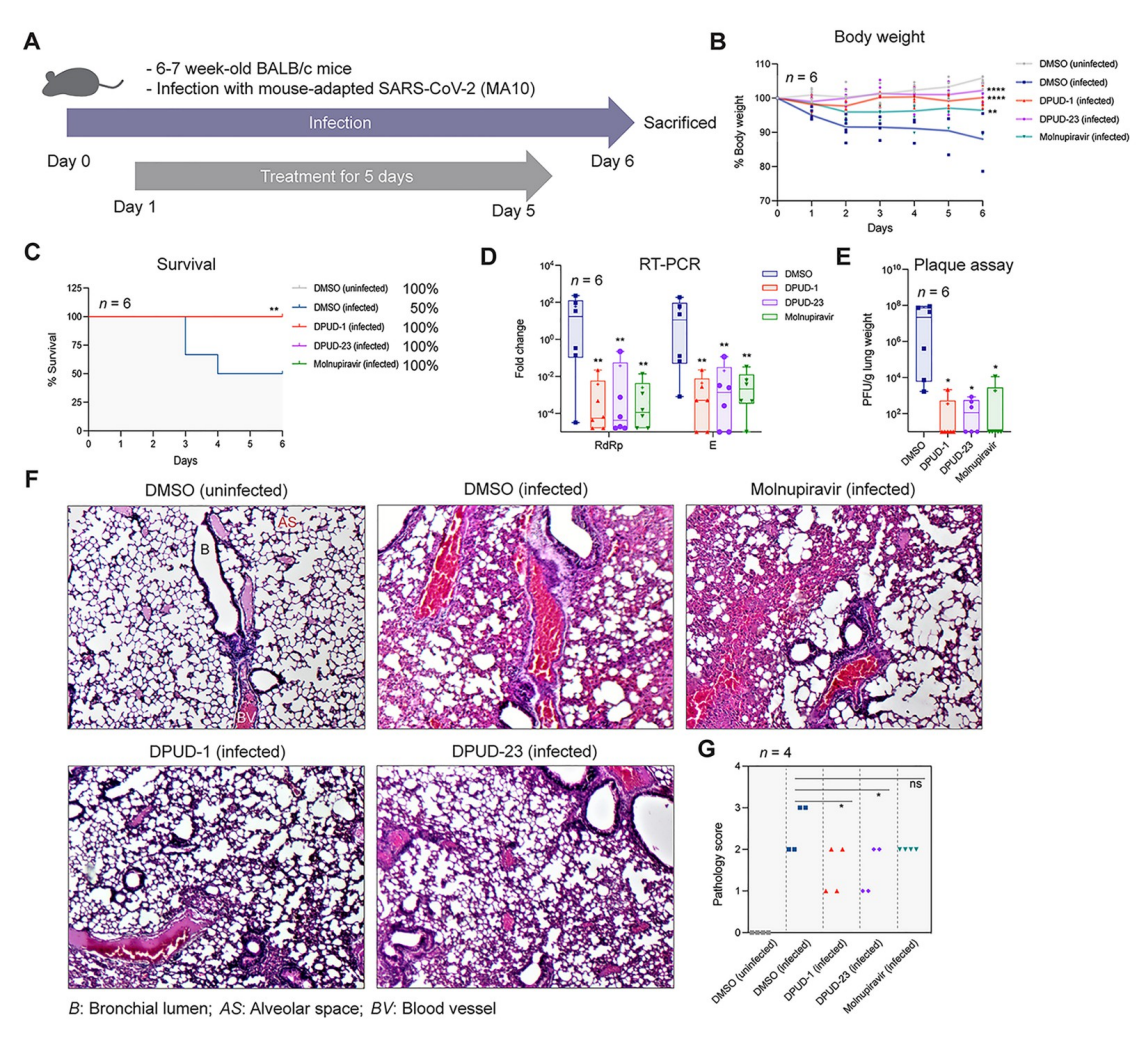
DPUD-1 and -23 protect mice against SARS-CoV-2 (MA10) infection. **(A)** Schematic representation of the *in vivo* experimental design. **(B)** Graph showing % body weights of uninfected mice, and SARS-CoV-2 (MA10)-infected mice treated with DPUD-1 (2 mg/kg/day) or -2 (2 mg/kg/day) or molnupiravir (50 mg/kg/day). **(C)** Survival curves of mice up to 6 days post-infection. **(D-E)** Quantification of lung viral titres by RT-PCR targeting SARS-CoV-2 RdRp and N genes, and by viral plaque assay. **(F)** Microscopy images of lung sections, stained with hematoxylin and eosin. **(G)** Statistical analysis of lung pathology scores. The *P*-value was determined using one-way ANOVA with multiple comparisons w.r.t. DMSO. ns: *P* >0.05, *P <0.05, **P <0.01, ***P <0.001, ****P <0.0001.

Finally, we tested the efficacy of the DPUDs against SARS-CoV-2 in mice. We performed the *in vivo* experiments including DPUD-1 and -23 as they exhibited the best inhibitory effects among the hit DPUDs against different SARS-CoV-2 variants in cell-based experiments. To test the efficacy of the DPUDs against SARS-CoV-2 infection in mice, we used a mouse-adapted SARS-CoV-2 (MA10), generated by Leist *et al*., which recapitulated the acute lung disease phenotypes commonly observed in humans infected with SARS-CoV-2 [51]. In an infection experiment with SARS-CoV-2 (MA10) in young BALB/c mice, Leist *et al*. observed that the pathogenesis of SARS-CoV-2 (MA10) depends on the infection dose of the mouse-adapted virus. The authors monitored the infectivity of 10^4^ PFU of SARS-CoV-2 (MA10) and the clinical symptoms in BALB/c mice up to 7 d.p.i. They reported that SARS-CoV-2 (MA10)-infected mice rapidly lost weight and reached maximum weight loss at day 4 post-infection, and the mice started to recover from 5 d.p.i [51]. We, therefore, carried out SARS-CoV-2 (MA10) challenge in BALB/c mice for 6 days in the present work. On day 0, we intranasally infected BALB/c mice with 10^4^ PFU of SARS-CoV-2 (MA10). Twenty four hour post-infection, we intraperitoneally administered DPUD-1 and -23 at 2 mg/kg of body weight once daily for 5 days, and sacrificed the animals on day 6 (**Fig 5A**). We included 6 mice per group and used DMSO and molnupiravir (50 mg/kg of body weight) as negative and positive controls, respectively. Infection with SARS-CoV-2 led to progressive body weight loss in the DMSO-treated mice till day 6, but DPUD-1 or -23-treated mice negligibly lost body weight during the initial two days post-infection and completely regained weight by day 3. Compared to molnupiravir, DPUD-1 or -23 treatment showed better recovery of body weight (**Fig 5B**). In contrast to 50% survival in the DMSO-treated mice, 100% survival was observed in mice treated with DPUD-1 or -23 or molnupiravir (**Fig 5C**). Next, we measured the lung viral titres by RT-PCR targeting the SARS-CoV-2 RdRp and E genes, and by viral plaque assay. Virus titres were significantly lower in mice treated with DPUD-1 or -23 or molnupiravir when compared to the DMSO-treated mice (**Fig 5D and 5E**). Lung sections were microscopically examined for pathology and evaluated for different pathological scores considering inflammation (alveolar and bronchial), haemorrhage and consolidation. The scores and parameters were graded as absent (0), minimal/mild (1), moderate (2), or severe (3). Lung pathology in the DMSO-treated mice included alveolar damage with interstitial lesions, inflammation, accumulation of immune effector cells, bronchial lining lesion, evidence of cell death and haemorrhage. Remarkable improvement was observed in the lungs of the mice treated with DPUD-1 or -23, which was in agreement with the body weight and survival data (**Fig 5F**). Lung pathology scores indicated significant improvement of the mice treated with DPUD-1 or -23 (**Fig 5G**). Taken together, the *in vivo* data suggest that DPUDs are highly effective against both IAV and SARS-CoV-2 infections and show potential for their therapeutic application.

## Discussion

SARS-CoV-2 presents the most consequential global health crisis after the 1918 influenza pandemic [52], and IAV poses consistent threat to public health by causing seasonal epidemics and occasional pandemics [53]. Protection against SARS-CoV-2 and IAV can be achieved through vaccination, but widespread vaccine-driven immunity remains a major challenge due to rapid evolution of these viruses and frequent emergence of immune-escape variants [54,55]. Although vaccination remains a cornerstone in prophylaxis, new therapeutic strategies are urgently required to broadly inhibit the existing and newly emerging SARS-CoV-2 variants, and to complement the already existing anti-influenza drugs with pharmacological agents with higher barrier to resistance. In antiviral research, the major focus has been directed in inhibiting viral proteins, because the virus-targeting inhibitors would not interfere with the host functions and would not cause major side effects. However, viral proteins, especially the proteins present on the viral surface, are under constant immune pressure and they rapidly accumulate mutations, often leading to the emergence of immune-escape variants. Host-directed antivirals targeting cellular proteins or pathways that viruses are dependent on for establishing infection have advantage over the inhibitors directly acting on viruses, as they are less likely to induce resistance through drug-mediated selective pressure. [56]. One of the obstacles of host-directed antiviral strategy is associated toxicity, as in order to inhibit viruses, host factors and processes critical for cell survival are often targeted, which may lead to undesired consequences on cell health. Therefore, clinical application of most of the host-directed interventions has been limited due to associated toxicity, and finding agents with potent antiviral activity but with minimal or tolerable toxicity has remained a major challenge in antiviral research [57].

In the life cycle of a virus, viral entry represents an attractive target for drug development as inhibition of this early infection step should prevent the subsequent infection processes [1]. To inhibit IAV entry, the major focus has so far been directed to blocking HA with small molecules or peptides that interfere with the binding of HA with the host sialic acids present on the cell surface glycoproteins and glycolipids [2]. However, the host-targeted strategies that are currently being explored to block IAV entry are rather limited, but among the few promising inhibitors, a sialidase fusion construct (DAS181), which cleaves sialic acid residues form the receptors, showed long-term effectiveness against influenza viruses. This recombinant protein with the sialidase catalytic domain and a sequence for mucosal cell surface anchoring has been shown to be nontoxic to the host cells and effective in animals both in pre- and post-exposure to the virus [58]. DAS181 administration for 5 days at 10 mg/kg/day showed significant reduction of viral load in a phase II trial, and the inhibitor is currently being tested in phase III clinical trials [2,59]. Other host-directed compounds including PitStop, chlorpromazine, Dynasore, Dyngo-4a, chloroquine, and Arbidol have been used *in vitro* to inhibit the entry of several viruses, but the *in vivo* efficacy against viral infections and toxicity assessment of majority of them have not yet been reported [2]. Recently, massive efforts were directed to identify antivirals against SARS-CoV-2 infection, but the majority of the potential antivirals are direct-acting, and only few of them have been reported to target the host with specific activity against viral entry. Among the host-directed SARS-CoV-2 inhibitors, niclosamide was identified, which blocks infection by neutralizing the endosomal pH and thereby inhibiting viral penetration [19]. However, the *in vivo* use of niclosamide against SARS-CoV-2 infection was limited due to its low aqueous solubility and poor bioavailability [60]. An oral formulation of niclosamide with magnesium oxide and hydroxy propyl methyl cellulose (NIC-MgO-HPMC) was tested in Syrian hamster model of SARS-CoV-2 infection, which showed potent activity against the virus [61]. Recently, a screen of peptidomimetic tetrapeptide compounds identified N-0385, which afforded high level of therapeutic and prophylactic benefit against SARS-CoV-2 in hACE2-expressing mice. N-0385 has been shown to provide protection against SARS-CoV-2 by blocking the activity of TMPRSS2 [62]. Another TMPRSS2 inhibitor, camostat, which is a serine protease inhibitor and used in the treatment of pancreatitis, has been repositioned as an anti-COVID-19 clinical candidate drug [63]. Compared to the large number of SARS-CoV-2- or IAV-specific entry inhibitors, only few antivirals have been identified so far that can commonly block cellular entry of both the viruses [64–68].

The DPUDs identified in this study robustly inhibited both SARS-CoV-2 and IAV infections, in tissue culture cells and in mice, without inducing any significant cytotoxicity. We found that the compounds blocked virus internalization by perturbing endocytic pathways, majorly affecting CME. Since endocytosis is critical to cell functioning and survival, it is generally believed that long-term perturbation of endocytic pathways would lead to compromised cell health and eventual death. Paradoxically, despite blocking intracellular trafficking pathways, the DPUDs did not affect the essential cellular functions to an extent that would lead to cell death. One possible explanation for this apparent lack of toxicity could be the fact that although the DPUDs interfered with the activity of some endocytic pathways, none of the pathways was completely blocked. Important ligands and cargoes essential for cell functioning could still enter the cell, albeit reduced uptake. Another possibility could be that while the endocytic pathways specifically utilized by the viruses were blocked by the DPUDs, other pathways were upregulated to compensate for the reduced nutrient uptake. However, further studies will be required to systematically study the effect of the DPUDs on all the known endocytic pathways and their regulations. Although the DPUDs inhibited SARS-CoV-2 and IAV internalization and blocked endosomal acidification, the unsubstituted compound i.e. DPU neither exhibited any antiviral effect nor showed any activity in neutralizing luminal acidity. Attachment of specific electron-withdrawing functional groups on the aromatic rings of DPU rendered the compounds highly potent in preventing virus internalization and in neutralizing endosomal pH. The calculated pKa values of the compounds indicate, the DPUDs have more acidity than DPU. Interestingly, among the DPUDs, DPUD-20 has the highest pKa value, and the same compound showed least potency compared to the others in inhibiting majority of the SARS-CoV-2 and IAV strains. Also, DPUD-20 exhibited mildest activity among the other compounds in *in vitro* Cl^-^ transport and neutralization of acidic vesicles. In contrast, DPUD-1 with the lowest pKa value robustly inhibited all the tested strains of SARS-CoV-2 and IAV, and completely neutralized the acidic pH of endosomes. DPUD-1 has the lowest pKa value compared to the other DPUDs due to the -I effect of the CF_3_ groups present at the *meta* positions, whereas DPUD-20 with the OCF_3_ groups at the *para* positions has an interesting overall effect on the pKa value, possibly due to the +M and -I effects of the OCF_3_ groups. In addition to DPUD-1, DPUD-2 and -23 also exhibited very high potency against all the tested viral strains. This indicates, although all the DPUDs have strong antiviral activity, their levels of effectiveness in infection inhibition may vary due to the differences in their chemical properties.

The time of addition assay with DPUD-1 showed marked reduction of IAV endocytosis in cells which were pre-treated with the compound. In this assay, the virus was allowed to enter the pre-treated cell, but in the absence of the inhibitor. Interestingly, we found that a pre-treatment of DPUD-1 was sufficient to block virus entry, and the inhibitory effect on virus endocytosis in the pre-treated cells persisted till 12 h post-removal of the compound. This suggests that a pre-treatment with the inhibitor is sufficient to disrupt the viral entry program to an extent that virus internalization is attenuated, even in its absence. Our experiments in the live cells with fluorescently-labelled IAV particles further demonstrated that DPUD-1 or DPUD-2 treatment prevents the viruses from reaching the cell interior, arresting most of the virus particles at the cell surface. Interestingly, we also found that in the inhibitor-treated cells, a considerable number of Rab5-positive early endosomes congregated at the plasma membrane, colocalized with the virus particles, and were relatively static compared to the other Rab5-positive vesicles. What causes these early endosomes to remain restricted at the plasma membrane without any apparent movement, needs further investigation. We also checked the cellular entry of fluorescently-labelled SARS-CoV-2, and found that DPUD-1 robustly blocked its internalization. Taken together, our results indicate that the DPUDs prevent cellular internalization of both IAV and SARS-CoV-2, which consequently leads to inhibition of viral replication.

SARS-CoV-2 enters the host cell either through the endosomal route or by fusing at the plasma membrane depending on the viral strain and the availability of TMPRSS2 at the cell surface. Several studies reported, TMPRSS2 inhibition reduces the capacity of the virus to infect the host cell, but the level of attenuation by the TMPRSS2 inhibitors is dependent on cell types and SARS-CoV-2 variants [14,69,70]. Inhibitors targeting TMPRSS2 either in TMPRSS2-deficient cells or in infection with the SARS-CoV-2 Omicron variants are ineffective in preventing infection [36]. Although the TMPRSS2 inhibitor camostat has been reported to effectively block SARS-CoV-2 infection in cultured cells [34,70], a double-blind, randomized controlled trial with hospitalized COVID-19 patients did not find the drug’s beneficial effects in reducing time to clinical improvement, progression to severe conditions, or mortality [63]. Since we identified the DPUDs in a screen against SARS-CoV-2 in Vero-E6 cells, which lack TMPRSS2, we addressed whether the compounds would still be effective in cells expressing TMPRSS2. We, therefore, infected TMPRSS2-expressing Vero-E6 cells treated with the DPUDs with SARS-CoV-2 (D614G) in presence/absence of camostat. We also included BafA1 and niclosamide as they have been reported to attenuate SARS-CoV-2 infection [19,69,71,72]. BafA1 is a vATPase inhibitor and niclosamide is a protonophore that disrupts proton gradient, and both the drugs potently inhibit endosomal acidification. Interestingly, we found all the DPUDs and also BafA1 and niclosamide almost completely neutralized infection in Vero-E6-TMPRSS2 cells, both in presence or absence of camostat. Previous reports suggested that lysosomotropic agents such as chloroquine would not be effective in TMPRSS2-expressing cells [73]. Contrary to these findings, a recent study showed that irrespective of TMPRSS2 expression, BafA1 potently inhibits SARS-CoV-2 infection and is 26-fold more potent than camostat in attenuating virus entry even in the presence of TMPRSS2 [69]. Also, the proton carrier niclosamide, which neutralizes the low-pH compartments, was found to strongly block the uptake of the receptor binding domain (RBD) of SARS-CoV-2 S glycoprotein, dextran, and transferrin [19]. Together, the above findings suggest that the agents which were previously considered to block infection by elevating endosomal pH at a post-internalization step, may have additional effects on virus internalization. However, the mechanism by which the endosomal pH-elevating compounds regulate virus internalization is currently unknown and underscores the need for further investigations. Our observation with the DPUDs, which robustly neutralize SARS-CoV-2 infection irrespective of TMPRSS2 expression, is in agreement with the inhibitory effect of BafA1 and niclosamide against the virus. We have demonstrated that like BafA1 and niclosamide, the DPUDs also neutralize endosomal acidity, but possibly through a distinct mechanism i.e. by transporting Cl^-^ into the cell and accumulating the ions into the endosomes/lysosomes. We propose that altered Cl^-^ homeostasis could be responsible for the observed dysfunction of the endocytic machinery. However, this proposed mode of action of the DPUDs is based on correlative evidence and further investigation will be required to precisely elucidate their mechanism of action in disrupting endocytic pathways.

Confirming the antiviral activity of DPUDs in tissue culture cells, we next evaluated their therapeutic potential in animal models of SARS-CoV-2 and influenza infections. Treatment with the DPUDs in mice challenged with SARS-CoV-2 or IAV led to remarkable body weight recovery, improved survival, and significantly reduced lung viral load as compared to the control mice. Taken together, our study identifies the DPUDs as extremely potent endocytosis inhibitors, which in addition to blocking SARS-CoV-2 and IAV entry, could be effective against a wide range of existing and emerging viruses that utilize common endocytic pathways. Although this study provides evidence of the inhibitory potency of the DPUDs against SARS-CoV-2 and IAV infections including the prevalent viral strains and VOC, their effectiveness against the other clinically relevant strains remains to be evaluated. In addition to blocking endocytic pathways, whether DPUDs target any cellular factor(s) critical for virus entry, remains to be investigated. Despite robust inhibition of the viruses during entry, the possibility remains that the DPUDs, in addition to blocking virus entry, also interfere with host factors or processes involved in post-entry infection events. The *in vivo* evaluation of the DPUDs against SARS-CoV-2 infection was performed using mouse model and mouse-adapted SARS-CoV-2 (MA10). However, the *in vivo* efficacy of the compounds remains to be tested and confirmed in other animal models such as Syrian hamsters. To further advance the hit DPUDs into antiviral development process, the physicochemical properties such as absorption, distribution, metabolism, excretion, pharmacokinetics, pharmacodynamics etc. should be investigated.

In conclusion, our study reports the identification of DPUDs as a new class of endocytosis inhibitors that broadly inhibit SARS-CoV-2 and IAV entry without inducing any significant cytotoxicity. The DPUDs majorly target clathrin-mediated pathway and they block endocytosis possibly by perturbing intracellular Cl^-^ homeostasis. DPUD-1 is effective against IAV infection in mice and has higher barrier to resistance than the anti-influenza drug oseltamivir. Treatment with DPUD-1 and -23 of SARS-CoV-2-infected mice leads to remarkable body weight recovery, improved survival and significant reduction in lung viral load. We believe, the identification of DPUDs as well-tolerated endocytosis inhibitors with strong antiviral properties would pave the way for developing a new class of antivirals with the potential to restrict a wide range of emerging and re-emerging viruses.

## Materials and methods

### Ethics statement

Institutional Animal Ethics Committee (IAEC) of IISc Bangalore and IISER Mohali were set up by the Committee for the Purpose of Control and Supervision of Experiments on Animals (CPCSEA), established under Chapter 4, Section 15(1) of the Prevention of Cruelty to Animals Act 1960. IAEC approved the experimental protocols for SARS-CoV-2 animal studies (CAF/Ethics/723/2019) and influenza A virus animal studies (IISERM/SAFE/PRT/2021/003). All operations were carried out in exact accordance with the authorised guidelines.

### Experimental model and subject details

#### Cells

A549, MDCK, HeLa, and HEK293T cells were obtained from American Type Culture Collection (ATCC). HEK293T-hACE-2 cells were obtained from BEI Resources, NIH (#NR-52511). Vero-E6 cells were acquired from the cell repository at National Centre for Cell Science (NCCS) Pune, India. Vero-E6-TMPRSS2 cells were acquired from the JCRB Cell Bank (#1818). All cells were maintained in DMEM (Gibco), supplemented with 10% FBS (Merck), Non-essential amino acids (Invitrogen), penicillin, streptomycin, and glutamine (Gibco) in 5% CO_2_ at 37°C.

#### Viruses

Purified IAV X-31(H3N2) was purchased from Microbiologics (previously Virapur), USA. IAV X-31 virus was propagated in pathogen-free chicken eggs at 33–37°C for 2 days, following which, the allantoic fluid was harvested. Virus was concentrated by two rounds of 10–40% sucrose gradient high-speed centrifugation, and the purified virus was harvested from the viral bands. The purified virus was resuspended in formulation buffer (40% sucrose, 0.02% BSA, 20 mM HEPES, pH 7.4, 100 mM NaCl, and 2 mM MgCl_2_) and aliquoted and stored at -80°C until use. The viral titre was determined in MDCK cells. The median tissue culture infectious dose (TCID_50_) of purified X-31 virus was determined as 1.95x10^9^. IAV WSN (A/WSN/1933) (H1N1), IAV Udorn (A/Udorn/72) (H3N2), IAV NYMC_X311 (A/Brisbane/1/2018) (H3N2) strains and Semliki Forest Virus (SFV-ZsG) were kind gifts from Yohei Yamauchi (ETH Zurich). IAV WSN, Udorn and NYMC strains were propagated in MDCK cells, and viral supernatant was clarified using centrifugation, and aliquots were stored at -80°C until use. SFV was propagated as described earlier [74], and stored at -80°C until use. SARS-CoV-2 virus was cultured by inoculating Vero-E6 cells with a fresh COVID-19 nasal swab sample. Viruses were confirmed to be SARS-CoV-2 D614G, Delta (B.1.617.2) and Omicron (B.1.1.529) strains by sequencing. Omicron BA.5 was obtained from BEI resources (Cat# NR-58616). The mouse-adapted SARS-CoV-2 (MA10) was obtained from BEI resources, NIH (#NR-55329). Experiments with IAV and SFV were carried out in BSL-2 laboratory, and all SARS-CoV-2-related experiments were performed in the BSL-3 laboratory with all the relevant ethical and biological safety clearances by institutional committees. Virus stock was titrated by using quantitative real time PCR (qRT-PCR) and plaque assay, and stored at -80°C until use.

#### Animal models

All the SARS-CoV-2 animal tests were performed with BALB/C mice, which were obtained from Adita Biosys, Tumkur. For IAV animal experiments, C57BL/6 mice were obtained from the Jackson Laboratory, USA, and bred in an individual ventilated caging system at the BSL-2 laboratory of Small Animal Facility for Experimentation (SAFE), IISER Mohali. Mice were housed in individually ventilated cages, maintained at 23±1°C temperature and 50±5% relative humidity, given access to standard pellet feed and water *ad libitum*, and maintained on a 12-hour day/night light cycle. After one week of acclimatization, mice were randomly grouped (n = 6).

## Method details

### Synthesis of 1,3-diphenylurea derivatives (DPUDs)

All reactions were carried out in oven-dried glassware. All solvents and starting materials were obtained from commercial sources. The developed chromatogram was analysed by UV lamp (254 nm) or p-anisaldehyde solution. Products were purified by silica gel (mesh size 230–400) column chromatography. The 1H NMR and 13C NMR spectra were recorded in deuterated chloroform (CDCl_3_) and deuterated methanol (MeOD) as per requirement. Chemical shifts of 1H and 13C NMR spectra are expressed in parts per million (ppm). All coupling constants are absolute values and are expressed in hertz. The description of the signals includes the following: *s* = singlet, *d* = doublet, *dd* = doublet of doublet, *t* = triplet, *dt* = doublet of triplet, *q* = quartet, *dq* = doublet of quartet, *br* = broad, and *m* = multiplet. Purity of the synthesized molecules was determined by HPLC chromatograms using SunFire (C8, 5 *μ*m, 4.6×250 mm) Waters HPLC column with acetonitrile and water as solvents. The HPLC chromatogram of DPUD-1 was obtained by using SunFire (Prep Silica, 5 *μ*m, 4.6×250 mm) Waters HPLC column with isopropyl alcohol and n-hexane as solvents. DPUD-1 synthesis was carried out as previously described [75]. The isocyanate was dissolved in tetrahydrofuran (THF), and the corresponding amine was added gravimetrically. After completion of the reaction (monitored by thin-layer chromatography, TLC), THF was removed under vacuum. The crude DPUD-1 was purified by washing with dichloromethane using vacuum filtration. DPUD-1 is also commercially available (Merck #803855). DPUD-2 to DPUD-23 were synthesized as previously described: (DPUD-2, -4, -6, -8, -11, -13, -17, -20) [76], (DPUD-3) [77], (DPUD-5) [78], (DPUD-7, -9, -21) [79], (DPUD-12) [80], (DPUD-14, -19) [81], (DPUD-15) [82], and (DPUD-22) [83]. 1,3-diphenyl urea (DPU) was procured from commercial source (Merck). To a solution of aromatic amine (1.0 equiv.) in CH_2_Cl_2_, DABCO (0.1 mmol, 0.1 equiv.) and (Boc)_2_O (0.5 equiv.) were successively added. After completion of the reaction as detected by TLC, the reaction mixture was cooled to 0°C and n-hexane was then added. The resulting solid was collected and further washed with cold water and diethyl ether to afford the corresponding DPUDs.

### High-content screening of DPUDs

IAV and SARS-CoV-2 infection assays were performed in IAV infection medium (DMEM with 0.2% BSA and 50mM HEPES pH 6.8) and SARS-CoV-2 infection medium (DMEM, serum-free), respectively. DPUDs, control inhibitors (BafA1, regorafenib and sorafenib for IAV infection; favipiravir, hydroxychloroquine (HCQ), and niclosamide for SARS-CoV-2 infection), and DPU were dissolved in DMSO to prepare stock solutions. Ten thousand A549 or Vero-E6 cells were seeded in each well of a 96-well optical-bottom plate (Greiner). Next day, cells were washed with infection medium. A549 cells were infected with IAV (X-31 and WSN) and Vero-E6 cells were infected with SARS-CoV-2 (D614G) at MOI: 0.2–0.6 in presence of DPUDs (10 *μ*M), DPU (10 *μ*M), BafA1 (50 nM), regorafenib (10 *μ*M), sorafenib (10 *μ*M), favipiravir (10 *μ*M), HCQ (10 *μ*M), and niclosamide (10 *μ*M) Ten h.p.i. (IAV) or 24 h.p.i. (SARS-CoV-2), cells were washed with phosphate buffered saline (PBS, pH 7.4) and fixed with 4% formaldehyde in PBS for 20 min at room temperature (RT). Indirect immunofluorescence (IIF) was performed with anti-NP (IAV) and anti-N (SARS-CoV-2) antibodies using previously-described protocols [9,29]. Nuclei were stained with Hoechst. The infection assays were performed in triplicate. Nine image fields were randomly selected in each well, and automatically imaged using a 20x objective with Yokogawa CQ1 high-content, dual-spinning disk, confocal microscope with maximum intensity projection of five Z-stacked images. Percentage infection was quantified by image analysis pipeline setup in CellProfiler (version 2.2.0) and KNIME (version 3.7.2). Data was plotted in GraphPad Prism 9 and statistical analysis was carried out using a one-way ANOVA with multiple comparisons.

### Determination of IC_50_ and CC_50_

To determine the IC_50_ values of the DPUDs, first, A549/Vero-E6 cells were seeded in optical bottom 96-well plates. Next day, DPUDs were serially diluted (10 *μ*M to 10 nM), and cells were pre-treated with respective dilutions of the compounds in infection medium at 37°C for 1 h. Following pre-treatment, the cells were infected with the viruses (IAV in A549 cells and SARS-CoV-2 in Vero-E6 cells) in presence of different concentrations of DPUDs, and incubated at 37°C for 10 h (IAV) or 24 h (SARS-CoV-2) in CO_2_ incubator. After fixing the cells, IIF was performed as described above to detect infected cells. IC_50_ was determined using non-linear regression function and plotted [inhibitor] vs. normalised response-variable slope. To determine the CC_50_ values of the DPUDs in A549 and Vero-E6 cells, colorimetric cytotoxicity assay was performed using LDH cytotoxicity assay kit (CytoTox 96 Non-Radioactive Cytotoxicity Assay, Promega) as per the manufacturer’s protocol. Briefly, ten thousand A549 or Vero-E6 cells were seeded in 96-well plates. Next day, the cells were treated with serially diluted (500 *μ*M to 1 *μ*M) DPUDs in cell culture medium. LDH was measured 24 h post-treatment from the supernatant at 490 nm using Multiskan GO plate reader (Thermo Scientific). CC_50_ was calculated using non-linear regression function and plotted [inhibitor] vs. normalised response-variable slope using GraphPad Prism 9.

### IAV infection assay in various cell lines

Hela or MDCK cells (1x10^4^ per well) were seeded in an optical bottom 96-well plate. Next day, cells were pre-treated with DPUD-1, -2, -16, -20, and -23 (10 *μ*M), DPU (10 *μ*M), BafA1 (50 nM) and equal volume of DMSO in infection medium at 37°C for 1 h. Following pre-treatment, the cells were infected with the IAV (X-31 (H3N2)) (MOI ~ 0.2) in presence of 10 *μ*M compounds, and incubated at 37°C for 10 h in CO_2_ incubator. After fixing the cells, IIF against IAV-NP was performed as described above to detect the infected cells.

### IAV (NYMC/ UDORN) infection assay

A549 cells (1x10^4^ per well) were seeded in an optical bottom 96-well plate. Next day, cells were pre-treated with DPUD-1, -2, -16, -20, and -23 (10 *μ*M), DPU (10 *μ*M), BafA1 (50 nM) and equal volume of DMSO in infection medium at 37°C for 1 h. Following pre-treatment, the cells were infected with the IAV (NYMC (H3N2) or UDORN (H3N2) (MOI ~1) in presence of 10 *μ*M compounds, and incubated at 37°C for 10 h in CO_2_ incubator. After fixing the cells, IIF for IAV-NP was performed as described above to detect infected cells.

### SARS-CoV-2 (D614G/Delta/Omicron) infection assay

Vero-E6 cells (1x10^4^ per well) were seeded in an optical bottom 96-well plate. The next day, cells were pre-treated with DPUD-1, -2, -16, -20, and -23 (10 *μ*M), DPU (10 *μ*M), BafA1 (50 nM) Niclosamide (10 *μ*M) and an equal volume of DMSO in SARS-CoV-2 infection medium at 37°C for 1 h. Following pre-treatment, the cells were infected with the SARS-CoV-2 (D614G or Delta B.1.617.2 or Omicron B.1.1.529 or Omicron BA.5) at MOI ~0.5 in presence of 10 *μ*M compounds, and incubated at 37°C for 24 h in CO_2_ incubator. After fixing the cells, IIF for SARS-CoV-2 N or Spike protein was performed as described above to detect the infected cells.

### SARS-CoV-2 infection assay in Vero-E6-TMPRSS2 cell line

To examine the effect of DPUDs in TMPRSS2 expressing cells, Vero-E6 stably expressing TMPRSS2 or Caco-2 cells (1x10^4^ per well) were seeded in an optical bottom 96-well plate. Next day, cells were pre-treated with 10 *μ*M DPUDs (DPUD1, DPUD-2, DPUD-16, DPUD-20 and DPUD23), DPU, niclosamide and equal volume of DMSO in presence and absence of camostat mesylate (50 *μ*M) (Merck) in infection medium at 37°C for 1 h. Following pre-treatment, the cells were infected with the SARS-CoV-2 (D614G) (MOI ~1) in presence of compounds in presence and absence of camostat mesylate, and incubated at 37°C for 24 h in CO_2_ incubator. After fixing the cells, IIF for SARS-CoV-2 N protein was performed as described above to detect infected cells.

### Semliki forest virus infection assay

BHK-21 cells (1x10^4^ per well) were seeded in optical bottom 96-well plate. Next day, cells were pre-treated with 10 *μ*M DPUDs (DPUD1, DPUD-2, DPUD-16, DPUD-20 and DPUD23), DPU, BafA1 and equal volume of DMSO in infection medium at 37°C for 2 h. Following pre-treatment, the cells were infected with the Semiliki forest virus (SFV-ZsG) at MOI ~0.5 in presence of 10 *μ*M compounds. Two h.p.i., the virus infection medium with the compounds was replaced with complete medium and the cells were incubated at 37°C for 8–10 h in a CO_2_ incubator. After fixing the cells, nuclei were stained with Hoechst and the SFV-GFP infected cells were counted as described above to detect the infected cells.

### qRT-PCR for measuring SARS-CoV-2 gene expression

To detect SARS-CoV-2 genes, Vero-E6 cells (1x10^4^) were seeded in a 96-well plate. Next day, the cells were pre-treated with 1 *μ*M of respective DPUDs in serum-free DMEM for one hour at 37°C, followed by infection with SARS-CoV-2 (MOI: 0.5) in presence DPUDs for 1 h. Medium was replaced with virus-free complete medium with DPUDs, and supernatant was collected at 24 h.p.i. SARS-CoV-2 E and N gene expression was detected and quantified using Quantiplus Multiplex (Huwel Lifesciences) or DiAGSure (GCC Biotech) COVID-19 detection kit. The analysis of the virus inactivation was based on the quantification of viral RNA (cycle threshold [Ct] profile) present in the culture supernatant.

### Western blotting and viral plaque assay

A549 or Vero-E6 cells were seeded in a 6 well plate (2x10^5^ cells/well) 24 h prior to infection. Cells were pre-treated with DPUDs (10 *μ*M) or DMSO at 37°C for 1 h. Following pre-treatment, the cells were infected with IAV X-31 or WSN or SARS-CoV-2 (D614G) (MOI: 0.1) and incubated for 24 h at 37°C in a humidified chamber with an atmosphere of 5% CO_2_. The supernatant was collected and centrifuged to remove cellular debris. For plaque assay, MDCK cells were seeded at a density of 2x10^5^ in a 12-well plate. Next day, the supernatant containing viruses was serially diluted till 10^−6^ dilution, and MDCK cells were inoculated with supernatants for 1 h. Cells were washed with PBS, and they were overlaid with infection medium with 0.8% low melting agarose (Genaxy) and 1 *μ*g/mL TPCK trypsin (Merck). The cells were fixed at 48 h.p.i. with 4% formaldehyde for 1 h. Agarose plug was carefully removed and the cells were stained with crystal violet solution (Himedia) for 15 min. Excess stain was removed by holding the plate under controlled flowing water. Viral plaques were counted, and PFU/mL was calculated. For western blotting, cells infected with IAV X-31 or WSN or SARS-CoV-2 (D614G) were lysed using RIPA buffer (CSH protocols) in presence of a protease inhibitor cocktail (Merck). The whole-cell lysate was vortexed at 4°C for 15 min, followed by centrifugation at 12000 rpm at 4°C for 30 min. The clarified lysate was mixed with 6x SDS loading dye and treated at 95°C for 10 min. SDS-PAGE was performed, and the proteins were transferred on nitrocellulose membrane (Bio-Rad). IAV hemagglutinin (HA) was stained with rabbit anti-HA (Pinda) antibody, SARS-CoV-2 Spike was stained with mouse anti-spike S309, and the loading control, GAPDH, was stained rabbit anti-GAPDH antibody (CST). HRP-labelled secondary antibodies (CST) were illuminated with ECL substrate mix (Bio-Rad), and the blot was developed using ImageQuant LAS 4000 system.

### IAV cellular entry assays

IAV cellular entry assays (virus binding, endocytosis, HA acidification, M1 uncoating, and vRNP nuclear import) were performed as described earlier [29]. Briefly, A549 cells were seeded in 96-well plate and after they reached 70–80% confluency, IAV entry assays were performed. In every assay, the cells were pre-treated with DPUDs (10 *μ*M) or DMSO at 37°C for 1 h. In endocytosis assay, chlorpromazine (CPZ) (70 *μ*M) was added as positive control. BafA1 (50 nM) was included as positive control in the HA acidification, M1 uncoating, and vRNP nuclear import assays. In the virus binding assay, 0.25 *μ*L of purified X-31 (TCID_50_: 1.95x10^9^) viruses were added in each well of a 96-well plate, and the viruses were allowed to bind to cells at 4°C for 1 h. Unbound viruses were removed by multiple washes with ice-cold PBS. Cells with the bound viruses were fixed with 4% formaldehyde on ice for 10 minutes, and they were further incubated with the fixative at RT for 10 min. In the endocytosis and HA acidification assays, 0.25 *μ*L of purified X-31 was added in each well, whereas, 0.50 *μ*L of the virus was added in each well for the M1 uncoating and vRNP nuclear import assays. Viruses were allowed to enter cells in presence of DPUDs (10 *μ*M) at 37°C for different time durations: 30 min (endocytosis), 1 h (HA acidification), 2.5 h (uncoating), and 4 h (vRNP nuclear import). After virus entry, the cells were fixed with 4% formaldehyde at RT for 20 min. All the virus entry assays were performed in presence of cycloheximide (1 mM) to prevent synthesis of new viral proteins. In the endocytosis assay, first, a saturating concentration (1:1000) of a rabbit polyclonal anti-HA antibody (Pinda), diluted in a non-permeabilizing solution (1% BSA and 5% FBS in PBS) was used to block all the HA epitopes present on the surface-bound virus. Upon permeabilization with saponin-containing buffer (0.1% saponin, 1% BSA, and 5% FBS in PBS), a mouse monoclonal anti-HA1 antibody (H3SKE) (1:100) was used to detect the internalized virus. The acid conformation of HA was detected using an acid conformation-specific mouse monoclonal antibody (A1) (1:1000). Viral M1 was stained with HB64 antibody (1:250), and NP was stained with HB65 antibody (1:30). Appropriate AF-conjugated secondary antibodies (Invitrogen) were used to detect the IAV proteins, and the nucleus was stained with Hoechst (Invitrogen). Imaging was done with 40x objective using Yokogawa CQ1 high-content, dual-spinning disk confocal microscope with maximum intensity projection of five Z-stacked images.

### Plasma membrane bypass infection assay

Plasma membrane (PM) bypass infection assay was carried out as described previously [42]. Briefly, A549 cells (1x10^4^) were seeded in optical bottom 96-well plates. Next day, cells were pre-treated with DPUDs (10 μM) or DMSO at 37°C. Following pre-treatment, viruses (0.5 *μ*l of purified X-31 in 50 *μ*L infection medium/well) were allowed to bind to the PM at 4°C for 1 h in presence of DPUDs (10 *μ*M). Pre-warmed fusion medium (DMEM with citrate buffer, pH 5.0) or STOP medium (DMEM with 50 mM HEPES, pH 7.4 and 20 mM NH_4_Cl) was added to cells and quickly shifted to 37°C for 3 min. Cells were rapidly washed with stop medium and incubated in the STOP medium for additional 10 h. Cells were then washed with PBS, fixed with 4% formaldehyde, and processed for IIF staining against IAV NP and imaging as described above.

### Live-cell imaging to monitor IAV internalization

To fluorescently label IAV (X-31) particles for live-cell imaging, the lipophilic dye SP-DiOC18(3) (Invitrogen) was used. First, the dye was dissolved in ethanol to prepare 1.8 mM stock solution. Next, 100 *μ*L purified IAV (X-31, TCID_50_: 1.95x10^9^) was diluted in 1400 *μ*L PBS. A final concentration of 2 μM SP-DiOC18(3) from the stock of 1.8 mM was used to label the virus diluted in a total volume of 1500 *μ*L (100 *μ*L virus+1400 *μ*L PBS). The dye was slowly added to the virus while vortexing at low speed. Following dye addition, the virus was incubated at RT for 1 h on a rotary mixer. The labelled virus was passed through a 0.2 *μ*m filter and used fresh for live-cell imaging. For live-cell imaging, A549 cells (5x10^4^) were seeded on a 35 mm glass bottom μ-dish (Ibidi). The cells were transfected with Rab5-RFP (Addgene #14437) using lipofectamine LTX (Invitrogen), 16–18 h prior to live-cell imaging. One hour before live-cell imaging, the cells were incubated with DPUD-1, DPUD-2 (10 *μ*M) or DMSO. After pre-treatment, the medium was replaced with serum-free medium (100 *μ*L) containing SP-DIOC18(3)-labelled virus particles and incubated at 37°C for 15 min in presence of DPUD-1, DPUD-2 or DMSO, allowing virus entry. Next, the cells were washed with serum-free medium to remove unbound viruses and replaced with fresh serum-free medium. Imaging was done using alpha plan apochromat 100x/1.46 N.A. oil objective in Zeiss LSM 980 with Airyscan 2 system.

### Pseudotyped SARS-CoV-2 preparation and entry inhibition assay

Viral entry inhibition assay was performed with pseudotyped SARS-CoV-2 as previously described [43]. Briefly, HEK293T cells were transfected with plasmid DNA pHIV-1 NL4·3Δenv-Luc and Spike-expressing construct. The spike plasmid expresses spike protein of D614G or Delta or Omicron variants. In all the three spike constructs, D614G mutation was present, which enhances the infectivity of the pseudovirus. The transfection was done by using Profection mammalian transfection kit (Promega Inc.) following the instructions of the kit manual. Pseudovirus decorated with SARS-CoV-2 spike proteins were harvested 48 h after transfection, clarified by filtration using 0.45 *μ*m filters, and stored at -80°C until use. 293T-hACE-2 (BEI resources, NIH, #NR-52511) cells expressing the ACE2 receptor were cultured in complete DMEM supplemented with 5% FBS. Entry-inhibition assays with the inhibitors were done in three replicates. The pseudovirus was incubated with serially diluted DPU/DPUDs/niclosamide and an equal volume of DMSO in a total volume of 100 *μ*L for 15 min at 37°C. The 293T-hACE2 cells were then trypsinised, and ten thousand cells/well were added to make up the final volume of 200 *μ*L/well. The plates were further incubated for 48 hrs in a humidified incubator at 37°C with 5% CO_2_. After incubation of cells, 140 *μ*L cell culture media was removed, and 50 *μ*L nano-Glo luciferase substrate (Promega Inc.) along with the lysis buffer was added. After lysing cells for 2–3 minutes, 80 *μ*L lysate was transferred to white plates, and luminescence was measured by using Cytation-5 multi-mode reader (BioTech Inc.) DMSO normalised RLU were plotted using GraphPad Prism.

### SARS-CoV-2 cellular entry assay

SARS CoV-2 (D614G) was labelled with the lipophilic dye SP-DiOC18(3) (Invitrogen). A final concentration of 2 *μ*M SP-DiOC18(3) from the stock of 1.8 mM was used to label the virus (SARS-CoV-2, PFU/mL: 1x10^7^) in a total volume of 2 mL viral supernatant. The dye was slowly added to the virus while vortexing at low speed. Following dye addition, the virus was incubated at RT for 1 h on a rotary mixer. The labelled virus was passed through a 0.2 *μ*m filter and used fresh for virus tracking. Vero-E6 cells (5x10^4^) were seeded on coverslips in 24 well plate. Cells were incubated with DPUD-1 (10 *μ*M) or DMSO for one hour. After pre-treatment, the labelled viruses were added to cells in presence of DPUD-1 or DMSO. Next, the cells were washed with 1x PBS to remove unbound viruses and replaced with DPUD-1 or DMSO containing media for 2 and 4 h. Cells were fixed with 4% formaldehyde and co-stained for nucleus and actin. Imaging was done using 63x oil objective using Leica TCS SP8 confocal microscope.

### EGF, transferrin, cholera toxin B and dextran uptake assays

To examine the effect of DPUDs in clathrin- or caveolin-mediated endocytosis, EGF, transferrin (Tfn) and cholera toxin B (CTxB) uptake assays were performed. Dextran (Dex) uptake assay was performed to check fluid-phase macropinocytosis. A549 cells (1x10^4^) were seeded in a 96-well optical-bottom plate. Next day, the cells were incubated with serum-free medium 2 h prior to the start of the experiments. Following incubation with serum-free medium, the cells were pre-treated with DPUDs (10 *μ*M) or DPU (10 *μ*M) or MβCD (10 mM) or imipramine (10 *μ*M) or DMSO in serum-free medium for 1h, or with CPZ (70 *μ*M) for 10 min at 37°C. Cells were pulsed with EGF-AF488 (30 ng/mL) (Invitrogen) for 30 min, Tfn-AF488 (30 *μ*g/mL) (Invitrogen) for 10 min, CTxB-AF488 (1 *μ*g/mL) (Invitrogen) for 15 min, and with Dex-AF488 (100 *μ*g/mL) (Invitrogen) 60 min in presence of the compounds in complete medium (with serum) at 37°C. In the Dex uptake assay, macropinocytosis was stimulated by the addition of phorbol myristate acetate (1 *μ*g/mL) (Merck). After respective time durations, cells were washed twice with PBS, followed by treatment with acidic buffer (150 mM NaCl and 50 mM Glycine, pH 3.0) for 2 min to remove the surface-bound ligands. Cells were again washed with PBS and fixed with 4% formaldehyde for 20 min at RT. Nucleus was stained with Hoechst diluted in PBS (1:10000). High-content microscopy and image analysis were performed as described above.

### Vesicular acidification assay

A549 cells (1x10^4^) were seeded in an optical-bottom, 96-well plate. Next day, the cells were pre-treated with DPUDs (10 *μ*M) or DPU (10 *μ*M) or BafA1 (50 nM) or DMSO at 37°C for 1 h. Next, the cells were incubated with LysoTracker Red DND-99 (1 *μ*M) (Invitrogen) in presence of the compounds at 37°C for 1 h. The cells were washed with 1xPBS and fixed with 4% formaldehyde. The nucleus was stained with Hoechst. High-content microscopy and image analysis were performed as described above.

### *In vitro* Cl^-^ transport assay

Lucigenin-based Cl^-^ transport assay was performed as previously described [48]. Briefly, a mixture of phospholipids (30 mg/mL) with 90% asolectin (Merck) and 10% cholesterol (Merck) in chloroform was prepared and transferred in a small round bottom flask. Lipids were dried on the internal glass surface via rotary evaporation. The lipid film was further dried overnight under a high vacuum. The lipid film was hydrated with internal buffer (1 mM lucigenin (N,N′-Dimethyl-9,9′-biacridinium dinitrate (Merck)), 222 mM KNO_3_, 5 mM HEPES, pH 7.4), and vortexed for 10 min. The lipid suspension was extruded eleven times through a 100 nm polycarbonate membrane (Nucleopore) using mini extruder set (Avanti Polar Lipids, Inc.). The resulting large unilamellar vesicles (LUVs) were separated from unencapsulated lucigenin by size exclusion chromatography using a Sephadex column (G-50 medium). The concentrated stock liposome solution was used freshly. To quantify Cl^-^ transport across LUV, a reaction mix (2 mL) was prepared with lucigenin-containing LUV in external buffer (222 mM KNO_3_, 5 mM HEPES pH 7.4, 25 mM NaCl). The mix was added into a 2 mL quartz cuvette with a small magnetic bead stirrer. Lucigenin fluorescence was monitored in a spectrofluorometer (FluoroMax-4, Horiba Scientific), equipped with a Peltier-based temperature controller set at 25°C, and using the excitation and emission wavelengths set at 430 nm and 505 nm, respectively, for 5 minutes. DPUDs (10 *μ*M) or DPU (10 *μ*M) or DMSO were added 30 sec post-acquisition start time. Percentage fluorescence vs. time was plotted using GraphPad Prism 9. For intracellular chloride sensing experiment, A549 cells (5x10^4^) were seeded in a 35mm glass-bottom *μ*-dish (Ibidi). Next day cells were transfected with mClY (Addgene #90457) [50] and LAMP1-mCherry (Addgene#55073), 16–18 h prior to live cell imaging. Two hours before live-cell imaging, the cells were incubated with DPUDs (10 *μ*M) or DMSO. After pre-treatment, imaging was performed using plan apochromat 63x/1.40 N.A. oil objective in Zeiss LSM 980 with Airyscan 2 system.

### *In vitro* calcein release assay

Calcein release assay was performed as previously described [49]. Briefly, 100 mM calcein (Merck) was prepared with 1 mg/mL phospholipid mix (Asolectin (90): cholesterol (10)) in an internal buffer (20 mM Tris-HCl, 150 NaCl, pH 8.0). Purified calcein-encapsulated LUV was prepared in 2 ml reaction mix with an external buffer (20 mM Tris-HCl, 150 NaCl, pH 8.0). DPUDs (10 *μ*M) or DPU (10 *μ*M) or DMSO were added 30 sec post-acquisition start time. Six mM Sodium deoxycholate (a detergent) was used as a positive control for calcein release. Percentage calcein release vs. time was plotted using GraphPad Prism 9.

### SARS-CoV-2 animal experiments

For SARS-CoV-2 (MA10) infection, 6–7-week-old female BALB/c mice were anesthetized with xylazine (4.5 mg/kg/body weight) and ketamine (90 mg/kg/ body weight) cocktail intraperitoneally. The test level of anesthesia was examined by pinch reflex. Mice were intranasally infected with 10^4^ PFU of mouse adapted SARS-CoV-2 strain MA-10. The investigator adequately monitored anesthetic recovery. After 24 h.p.i., the mice were intraperitoneally administered with DPUD-1 and DPUD-23 (2 mg/kg of mouse body weight) or Molnupiravir (50 mg/kg of mouse body weight) in DMSO/PBS or an equal volume of DMSO dissolved in PBS once a day for the next 5 days. The health and clinical signs of mice were daily monitored by an expert veterinarian. On day 6 post-infection, the mice were humanely sacrificed by cervical dislocation and the whole lung was taken out and weighed. The whole left lobe of the lungs was fixed in 4% PFA for histopathological analysis and the remaining upper right lobes were homogenized in DMEM media for the titration of the virus by plaque assays. SARS-CoV-2 plaque assays were performed in six-well plates by infecting the Vero-E6 cells, similarly to the IAV plaque assay described above. The remaining right lower lobes were homogenized in RNAiso Plus (Takara) for the enumeration of post-treatment virus copy in the lung of mice through RT-PCR. The viral RNA copy number was quantified by estimation of the CT value of the E and RdRP genes of mouse-adapted SARS-COV-2, using Q-line-ER, (nCoV-19) RT-PCR detection kit.

### Histopathology

For histopathological examination, after necropsy, the left lobe of the SARS-CoV-2 infected lung of each mouse was fixed in 4% of paraformaldehyde. Fixed lungs were embedded in paraffin and cut into a 4 *μ*m section by microtome and mounted on glass slides. The lung sections were stained with hematoxylin and eosin. A veterinary immunologist inspected and scored the lung sections microscopically for distinct pathological scores, taking into account the lung inflammation (alveolar and bronchial) and consolidation. The scores and parameters were graded as absent (0), minimal/mild (1), moderate (2), or severe (3).

### IAV animal experiments

For IAV infection, 6–10-week-old C57BL/6 mice were anesthetized as described above. Mice were intranasally infected with 1000 PFU of IAV (WSN). The investigator adequately monitored anesthetic recovery. After 24 h.p.i., the mice were intraperitoneally administered with DPUDs or oseltamivir acid or an equal volume of DMSO dissolved in PBS (1 mg/kg of mouse body weight) twice a day for the next 5 days. Bodyweight was monitored every day. After ten days, animals were euthanized using CO_2_ asphyxiation. Lungs and spleens were excised and homogenized in DMEM. RNA was isolated from tissue homogenate using Tri reagent (Merck) and qRT-PCR was performed for detection of IAV NP expression using the primers: 5’GGAGTCTTCGAGCTCT3’ (fwd) and 5’TTTTTTTTTTTTTTTTTTTAATTGTCGTACTCCT3’ (rev). Plaque assay was performed using the lung homogenate supernatant in MDCK cells using the method described above.

### IAV resistance assay

Twenty-four hours prior to infection, MDCK cells (1x10^5^) were seeded in a 24-well plate. Cells were pre-treated with 1 *μ*M DPUD-1/oseltamivir acid and an equal volume of DMSO in the infection media for 1 h at 37°C. Cells were infected with IAV WSN (MOI: 0.1) for 1 h in presence of the compounds. Cells were washed with 1xPBS and replaced with infection medium with TPCK trypsin (1 *μ*g/mL) and the compounds. After 48–72 h.p.i., the supernatant was collected and clarified for cell debris by centrifugation, and 1:10 dilution of the supernatant was used for the next round of infection. Similarly, viruses were passaged nine more times, and after the 10^th^ passage, viral load was quantified by plaque assay.

### Image acquisition and analysis

Automated high-content image acquisition was performed using a 20x or 40x objective using the Yokogawa CQ1 dual-spinning disk confocal microscope. Nine (3x3) images were acquired from each well of an optical-bottom 96-well plate for each fluorescence channel, typically resulting in the counting of 3,000–7,000 cells in the samples. High-resolution images were acquired using Leica TCS SP8 confocal microscope or Zeiss LSM 980 with Airyscan 2 system. Images were processed in ImageJ (2.0.0-rc-69/1.52p) and Adobe Photoshop (21.2.12) for better visualization. Confocal images from multiple planes were Z-stacked by maximum intensity projection. Percentage infection was quantified by detection of cell nuclei and NP (IAV)- or N (SARS-CoV-2)-expressing cells by an image analysis pipeline created in Cell Profiler (version 2.2.0) and KNIME (version 3.7.2). IAV uncoating and vRNP nuclear import analysis were carried out using the same image analysis module. Integrated density (product of mean fluorescence intensity and area) was calculated for the other assays.

### Statistical analysis

Data are represented as the mean±SD. For all analyses, multiple independent experiments (*n* ≥ 3) were carried out. Statistical analysis was performed using Graphpad Prism 9, and the P value was calculated by one-way ANOVA with multiple comparisons or unpaired *t* test.

## Supporting information

S1 Fig Effect of DPUDs against IAV and SARS-CoV-2 infections in different cell lines. **(A)** The workflow of the high-content infection screens performed in A549 and Vero-E6 cells using IAV (X-31, H3N2 and WSN/33, H1N1) and SARS-CoV-2 (D614G) strains, respectively. **(B)** High-content microscopy images of IAV- and SARS-CoV-2-infected cells, treated with DMSO or DPU (10 *μ*M), DPU-1, -2, -16, -20, and -23 (10 *μ*M), or BafA1 (50 nM) or niclosamide (10 *μ*M). Nuclei were stained with Hoechst (magenta), and the viral NP/N proteins (green) were detected by IIF. Scare bars, 50 *μ*m. **(C)** Effect of DPU (10 *μ*M) and DPUDs (10 *μ*M) against IAV X-31 infection in MDCK and HeLa cells. **(D)** Effect of DPUDs (10 *μ*M) against SARS-CoV-2 (D614G) infection in Vero-E6-TMPRSS2 cells in presence/absence of camostat. *N* = 3 biologically independent experiments. All data are represented as mean ± SD. The *P*-value was determined using one-way ANOVA with multiple comparisons w.r.t. DMSO. ns: *P* >0.05, *P <0.05, **P <0.01, ***P <0.001, ****P <0.0001.
(TIF)

S2 Fig Effect of DPUDs against different strains of IAV and SARS-CoV-2. **(A)** Effect of DPUDs (10 *μ*M) against IAV NYMC (H3N2) infection in A549 cells. **(B)** Effect of DPUDs (10 *μ*M) against IAV Udorn (H3N2) infection in A549 cells. **(C)** Effect of DPUDs (10 *μ*M) against SARS-CoV-2 B.1.617.2 (Delta) infection in Vero-E6 cells. **(D)** Effect of DPUDs (10 *μ*M) against SARS-CoV-2 B.1.1.529 (Omicron) infection in Vero-E6 cells. **(E)** Effect of DPUDs (10 *μ*M) against SARS-CoV-2 BA.5(Omicron) infection in Vero-E6 cells. *N* = 3 biologically independent experiments. All data are represented as mean ± SD. The *P*-value was determined using one-way ANOVA with multiple comparisons w.r.t. DMSO. ns: *P* >0.05, *P <0.05, **P <0.01, ***P <0.001, ****P <0.0001.
(TIF)

S3 Fig DPUDs block cellular entry of IAV and SARS-CoV-2. **(A)** High-content images of IAV (X-31) cellular entry assays performed in DPUD-treated A549 cells. IAV entry was monitored for virus binding, endocytosis, HA acidification, nucleocapsid uncoating, and vRNP import. Nuclei were stained with Hoechst (magenta) and viral proteins (green), HA, HA (acid), M1, and NP were detected by IIF. Actin filaments (grey) were stained with Phalloidin-AF647. Cropped images of the high-content microscopy images are shown for better visualization. Scare bars, 50 *μ*m. **(B-D)** Graphs showing concentration-dependent effect of DPUDs on HIV-based pseudotyped SARS-CoV-2 (D614G, Delta and Omicron) infections in HEK 293T-hACE2 cells. The half-maximal inhibitory concentration (IC_50_) values corresponding to each compound and strain-specific pseudotyped SARS-CoV-2 are shown. **(E)** Confocal images of cellular entry of SP-DiOC18(3)-labelled SARS-CoV-2 (green) in Vero-E6 cells treated with DMSO or DPUD-1 (10 *μ*M) at 4 h.p.i. Nuclei were stained with Hoechst (grey). Scale bars, 20 *μ*m.
(TIF)

S4 Fig DPUDs block cellular uptake of epidermal growth factor (EGF), transferrin (Tfn), cholera toxin B (CTxB), dextran (Dex), and prevent vesicular acidification. High-content images of AF488-conjugated EGF (green), Tfn (green) and CTxB (green) uptake, and LysoTracker Red DND-99 (green) accumulation in DPUD-treated A549 cells. Nuclei were stained with Hoechst (magenta). Cropped images of the high-content microscopy images are shown for better visualization. Scare bars, 50 *μ*m.
(TIF)

S5 Fig DPUDs target endocytic processes and transport chloride ions across lipid membrane. **(A-F)** Confocal images of A549 cells treated with DPU, DPUD-2, -16, -20, -23 (10 *μ*M), and DMSO for 1 h. Antibodies were used to stain EEA1 (cyan) and LAMP1 (red). Phalloidin-AF647 and Hoechst were used to stain actin filaments (yellow) and nuclei (magenta), respectively. Scare bars, 20 *μ*m. **(G)** Lucigenin assay. Large unilamellar vesicles (LUVs) containing lucigenin were generated. In presence of chloride ions, DMSO, DPUD-2, -16, -20, -23 were added and the fluorescent intensity was measured. **(H)** Calcein release assay. Effect of DPUDs was examined by the release of calcein from LUVs.
(TIF)

S6 Fig DPUD-1 shows *in vivo* efficacy and high barrier to resistance against IAV infection. **(A)** Schematic representation of the experimental design of *in vivo* efficacy assessment of DPUDs against IAV infection. Six to ten week-old C57/BL6 mice (*n* = 6/group) were infected with 1000 PFU IAV (WSN) on day 0. The infected mice were intraperitoneally administered DPUDs/DMSO/Oseltamivir acid with 1 mg/kg of body weight twice daily from day 1 post-infection till day 6. Body weights of the mice were daily measured from day 0 till day 10, following which, the mice were sacrificed. **(B)** Graph showing % body weights of the uninfected mice, and IAV-infected mice that were treated with DPUD-1, Oseltamivir acid, and DMSO. **(C)** Survival of the mice up to 21 days post-infection. The mice (*n* = 5/group) were infected with 3000 PFU IAV (WSN) on day 0, following which they were intraperitoneally administered DPUDs/DMSO/Oseltamivir acid with 1 mg/kg of body weight twice daily for 5 days. **(D)** RT-PCR of IAV (WSN) NP gene from the lungs of infected mice (*n* = 3), sacrificed on day 6 post-infection. **(E)** Virus titres from the lungs of infected mice (*n* = 3), sacrificed on day 6 post-infection. **(F)** Images and results of virus plaque assays in MDCK cells from the supernatants of A549 cells infected with IAV (WSN). A549 cells were infected with the virus (MOI = 0.1) in presence of DPUD-1 (10 *μ*M) or oseltamivir acid (10 *μ*M) or DMSO. Supernatants were collected after 24 h post-infection, and virus plaque assays were performed. **(G)** Brightfield images and results of virus plaque assays from serial viral passage experiments. MDCK cells were infected with IAV (WSN) (MOI = 0.1) in presence of DPUD-1 (1 *μ*M) or oseltamivir acid (1 *μ*M) or DMSO. Supernatants were collected every 2/3 days and new cells were infected with the viruses present in the supernatants. Serial passaging of the virus was carried out for 10 passages after which, virus titres for each compound treatment were determined. The *P*-value was determined using one-way ANOVA with multiple comparisons w.r.t. DMSO. ns: *P* >0.05, *P <0.05, **P <0.01, ***P <0.001, ****P <0.0001.
(TIF)

S1 Data Excel spreadsheet containing numerical values used to generate graphs and statistical analysis for Figs 1A IAV-X31, 1A IAV WSN, 1A SARS-CoV-2, 1E, 1F, 2A, 2B, 2C, 2D, 2E, 2F, 3B IAV Binding, 3B IAV Endocytosis, 3B IAV Acidification, 3B IAV Uncoating, 3B IAV Nuclear Import, 3D, 3E, 4A EGF, 4A Tfn, 4A CTxB, 4A Dex, 4C, 4D, 4E, 4F, 4G, 5B, 5C, 5D, 5E, 5G, S1C, S1D, S2A, S2B, S2C, S2D, S2E, S3B, S3C, S3D, S5G, S5H, S6B, S6C, S6D, S6E, S6F, and S6G. (XLSX)

S1 Table Initial high-content screening of small molecules against IAV infection and identification of DPUD-1 as a potent inhibitor. (PDF)

S2 Table Structures of DPU and the DPUDs included in the high-content screenings against IAV and SARS-CoV-2 infection. (PDF)

S3 Table NMR data of the DPUDs. (PDF)

S4 Table HPLC data of the DPUDs. (PDF)
