# Supplementary figures and images for: Identification of diphenylurea derivatives as novel endocytosis inhibitors that demonstrate broad-spectrum activity against SARS-CoV-2 and influenza A virus both in vitro and in vivo
Source: PLoS Pathog. 2023 May 1;19(5):e1011358. doi: 10.1371/journal.ppat.1011358 (PMC10174524; doi:10.1371/journal.ppat.1011358)

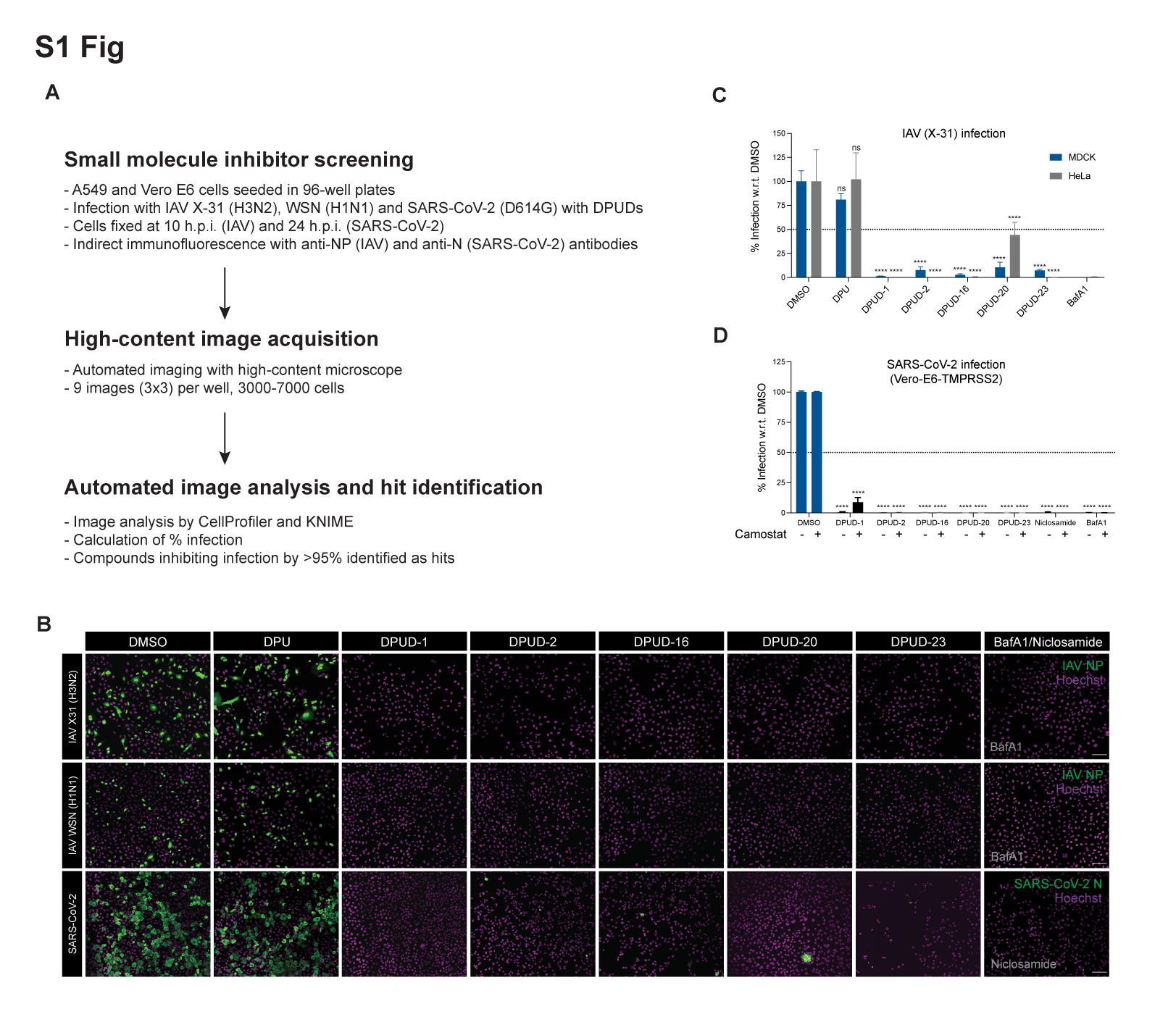

Supplement: S1 Fig — (A) The workflow of the high-content infection screens performed in A549 and Vero-E6 cells using IAV (X-31, H3N2 and WSN/33, H1N1) and SARS-CoV-2 (D614G) strains, respectively. (B) High-content microscopy images of IAV- and SARS-CoV-2-infected cells, treated with DMSO or DPU (10 μM), DPU-1, -2, -16, -20, and -23 (10 μM), or BafA1 (50 nM) or niclosamide (10 μM). Nuclei were stained with Hoechst (magenta), and the viral NP/N proteins (green) were detected by IIF. Scare bars, 50 μm. (C) Effect of DPU (10 μM) and DPUDs (10 μM) against IAV X-31 infection in MDCK and HeLa cells. (D) Effect of DPUDs (10 μM) against SARS-CoV-2 (D614G) infection in Vero-E6-TMPRSS2 cells in presence/absence of camostat. N = 3 biologically independent experiments. All data are represented as mean ± SD. The P-value was determined using one-way ANOVA with multiple comparisons w.r.t. DMSO. ns: P >0.05, *P <0.05, **P <0.01, ***P <0.001, ****P <0.0001. (TIF) [file ppat.1011358.s001.tif]

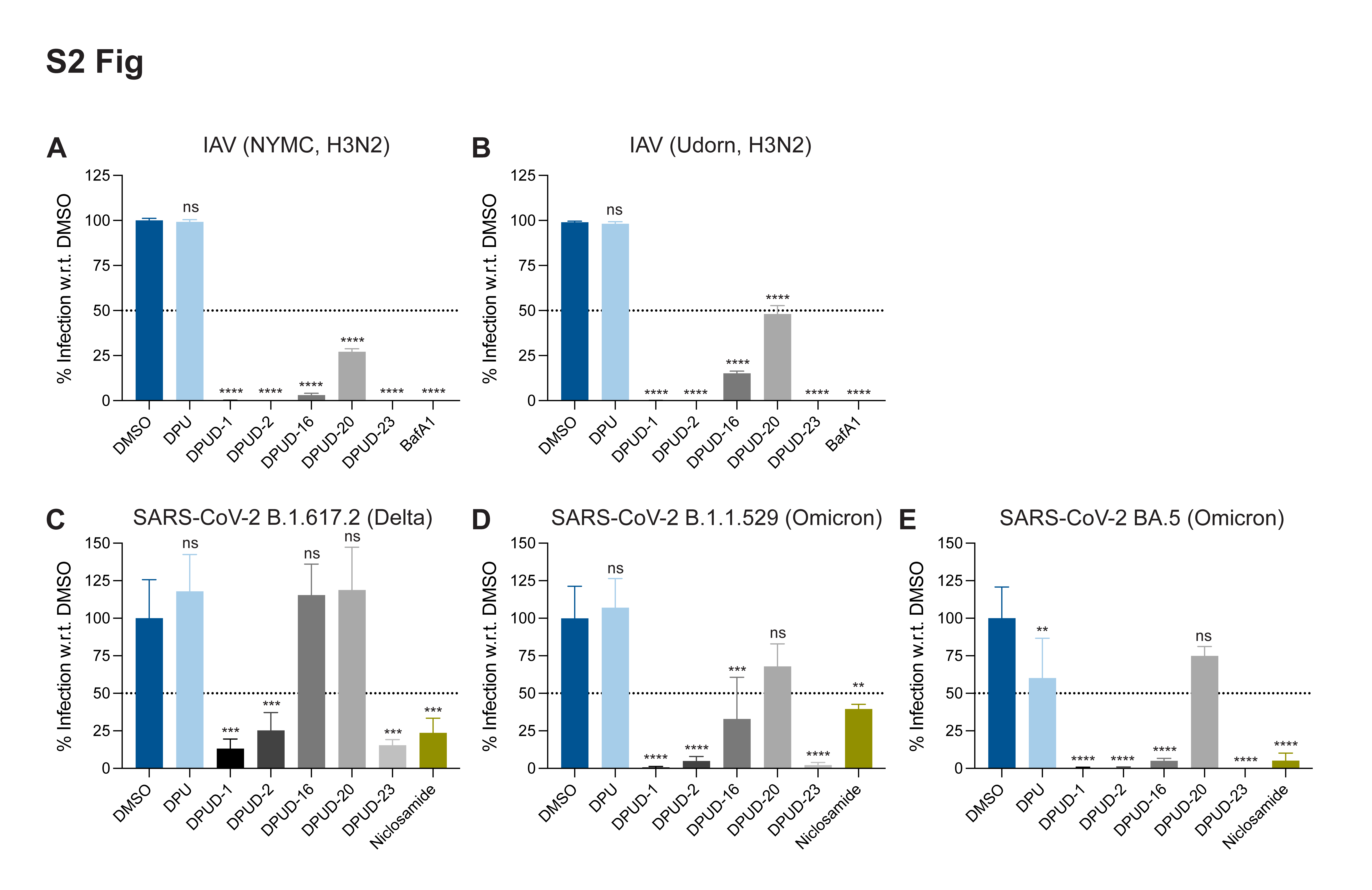

Supplement: S2 Fig — (A) Effect of DPUDs (10 μM) against IAV NYMC (H3N2) infection in A549 cells. (B) Effect of DPUDs (10 μM) against IAV Udorn (H3N2) infection in A549 cells. (C) Effect of DPUDs (10 μM) against SARS-CoV-2 B.1.617.2 (Delta) infection in Vero-E6 cells. (D) Effect of DPUDs (10 μM) against SARS-CoV-2 B.1.1.529 (Omicron) infection in Vero-E6 cells. (E) Effect of DPUDs (10 μM) against SARS-CoV-2 BA.5(Omicron) infection in Vero-E6 cells. N = 3 biologically independent experiments. All data are represented as mean ± SD. The P-value was determined using one-way ANOVA with multiple comparisons w.r.t. DMSO. ns: P >0.05, *P <0.05, **P <0.01, ***P <0.001, ****P <0.0001. (TIF) [file ppat.1011358.s002.tif]

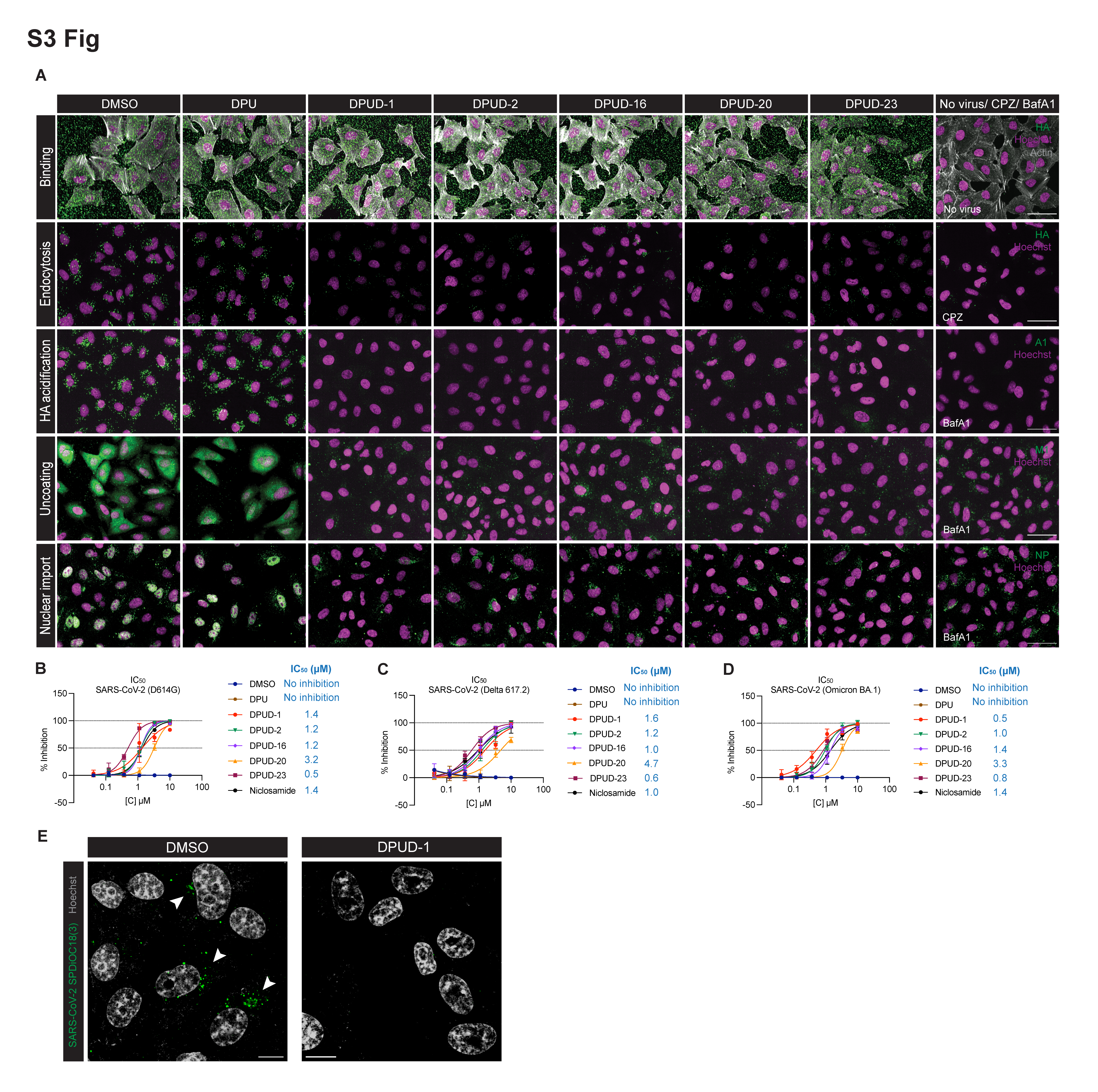

Supplement: S3 Fig — (A) High-content images of IAV (X-31) cellular entry assays performed in DPUD-treated A549 cells. IAV entry was monitored for virus binding, endocytosis, HA acidification, nucleocapsid uncoating, and vRNP import. Nuclei were stained with Hoechst (magenta) and viral proteins (green), HA, HA (acid), M1, and NP were detected by IIF. Actin filaments (grey) were stained with Phalloidin-AF647. Cropped images of the high-content microscopy images are shown for better visualization. Scare bars, 50 μm. (B-D) Graphs showing concentration-dependent effect of DPUDs on HIV-based pseudotyped SARS-CoV-2 (D614G, Delta and Omicron) infections in HEK 293T-hACE2 cells. The half-maximal inhibitory concentration (IC50) values corresponding to each compound and strain-specific pseudotyped SARS-CoV-2 are shown. (E) Confocal images of cellular entry of SP-DiOC18(3)-labelled SARS-CoV-2 (green) in Vero-E6 cells treated with DMSO or DPUD-1 (10 μM) at 4 h.p.i. Nuclei were stained with Hoechst (grey). Scale bars, 20 μm. (TIF) [file ppat.1011358.s003.tif]

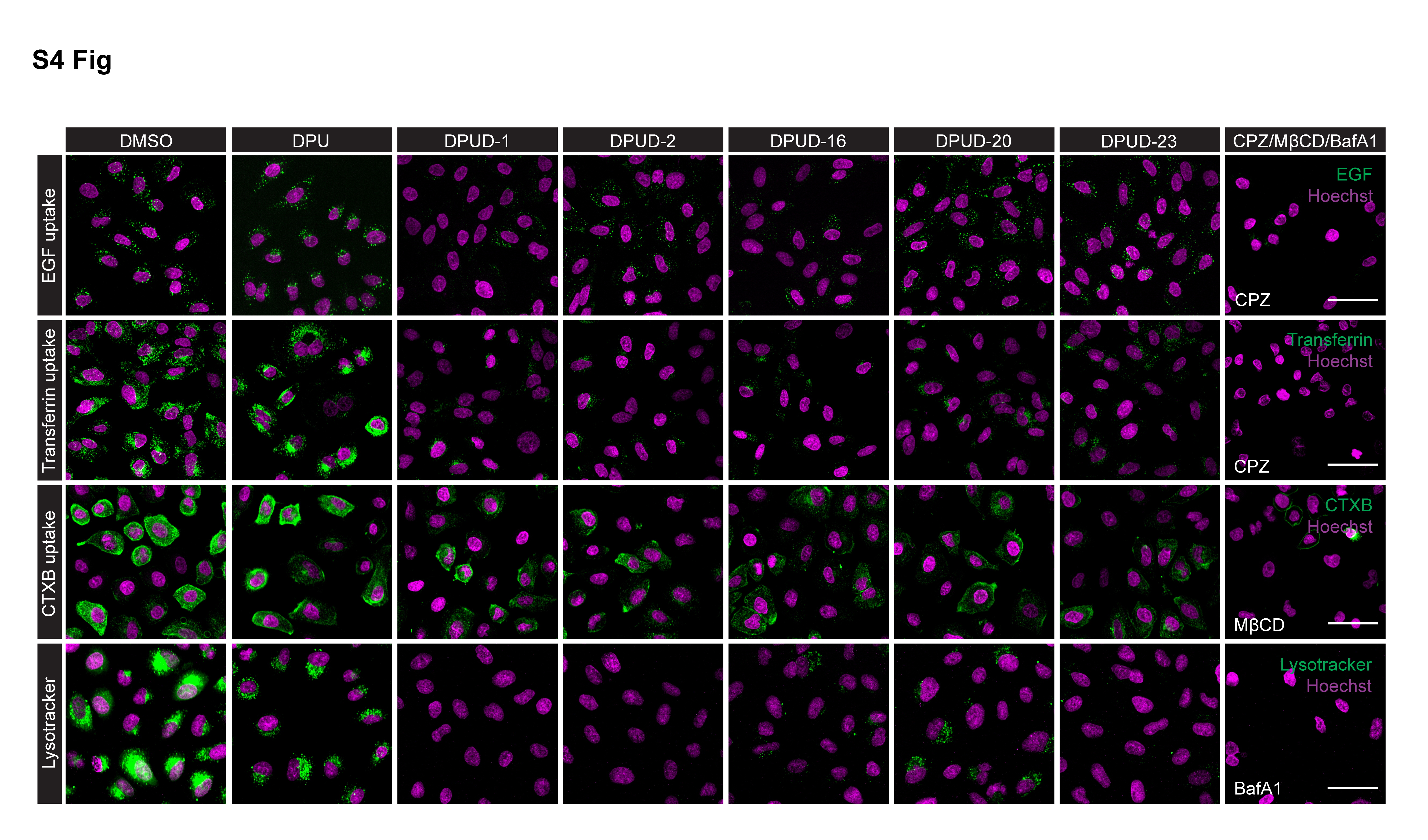

Supplement: S4 Fig — High-content images of AF488-conjugated EGF (green), Tfn (green) and CTxB (green) uptake, and LysoTracker Red DND-99 (green) accumulation in DPUD-treated A549 cells. Nuclei were stained with Hoechst (magenta). Cropped images of the high-content microscopy images are shown for better visualization. Scare bars, 50 μm. (TIF) [file ppat.1011358.s004.tif]

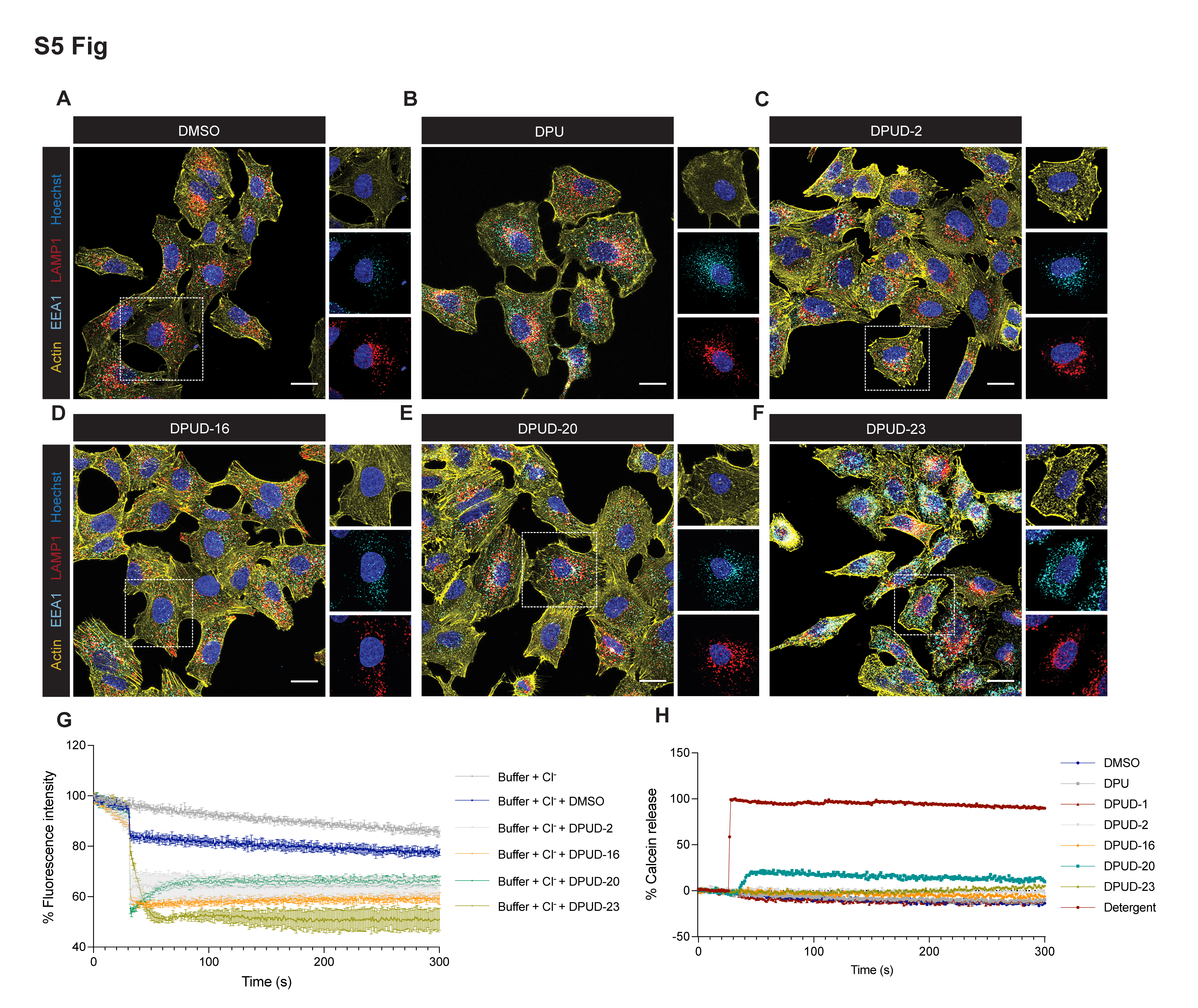

Supplement: S5 Fig — (A-F) Confocal images of A549 cells treated with DPU, DPUD-2, -16, -20, -23 (10 μM), and DMSO for 1 h. Antibodies were used to stain EEA1 (cyan) and LAMP1 (red). Phalloidin-AF647 and Hoechst were used to stain actin filaments (yellow) and nuclei (magenta), respectively. Scare bars, 20 μm. (G) Lucigenin assay. Large unilamellar vesicles (LUVs) containing lucigenin were generated. In presence of chloride ions, DMSO, DPUD-2, -16, -20, -23 were added and the fluorescent intensity was measured. (H) Calcein release assay. Effect of DPUDs was examined by the release of calcein from LUVs. (TIF) [file ppat.1011358.s005.tif]

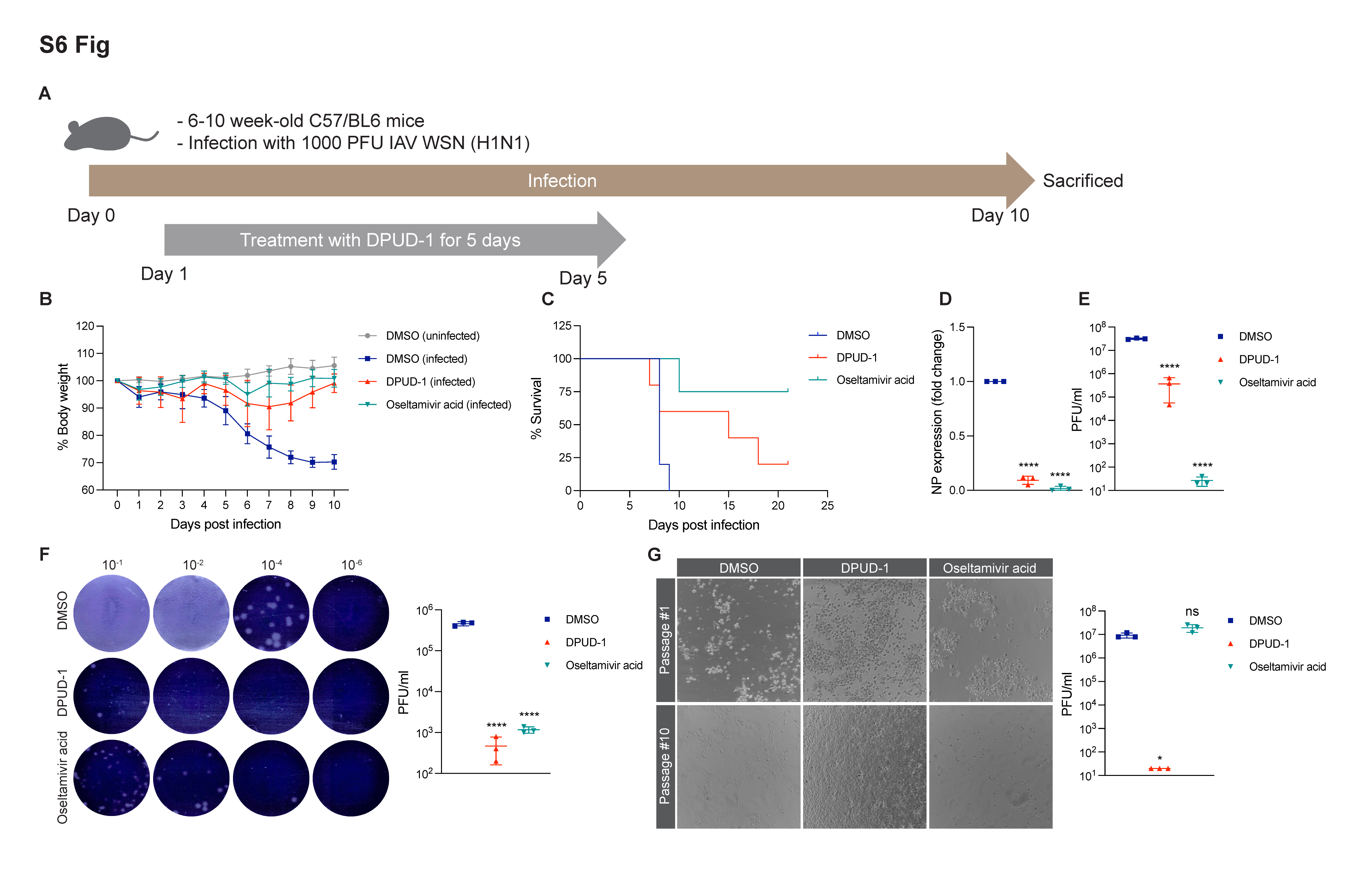

Supplement: S6 Fig — (A) Schematic representation of the experimental design of in vivo efficacy assessment of DPUDs against IAV infection. Six to ten week-old C57/BL6 mice (n = 6/group) were infected with 1000 PFU IAV (WSN) on day 0. The infected mice were intraperitoneally administered DPUDs/DMSO/Oseltamivir acid with 1 mg/kg of body weight twice daily from day 1 post-infection till day 6. Body weights of the mice were daily measured from day 0 till day 10, following which, the mice were sacrificed. (B) Graph showing % body weights of the uninfected mice, and IAV-infected mice that were treated with DPUD-1, Oseltamivir acid, and DMSO. (C) Survival of the mice up to 21 days post-infection. The mice (n = 5/group) were infected with 3000 PFU IAV (WSN) on day 0, following which they were intraperitoneally administered DPUDs/DMSO/Oseltamivir acid with 1 mg/kg of body weight twice daily for 5 days. (D) RT-PCR of IAV (WSN) NP gene from the lungs of infected mice (n = 3), sacrificed on day 6 post-infection. (E) Virus titres from the lungs of infected mice (n = 3), sacrificed on day 6 post-infection. (F) Images and results of virus plaque assays in MDCK cells from the supernatants of A549 cells infected with IAV (WSN). A549 cells were infected with the virus (MOI = 0.1) in presence of DPUD-1 (10 μM) or oseltamivir acid (10 μM) or DMSO. Supernatants were collected after 24 h post-infection, and virus plaque assays were performed. (G) Brightfield images and results of virus plaque assays from serial viral passage experiments. MDCK cells were infected with IAV (WSN) (MOI = 0.1) in presence of DPUD-1 (1 μM) or oseltamivir acid (1 μM) or DMSO. Supernatants were collected every 2/3 days and new cells were infected with the viruses present in the supernatants. Serial passaging of the virus was carried out for 10 passages after which, virus titres for each compound treatment were determined. The P-value was determined using one-way ANOVA with multiple comparisons w.r.t. DMSO. ns: P >0.05, *P <0.05, ** [file ppat.1011358.s006.tif]
