# Supplementary material for: Identification of diphenylurea derivatives as novel endocytosis inhibitors that demonstrate broad-spectrum activity against SARS-CoV-2 and influenza A virus both in vitro and in vivo
Source: PLoS Pathog. 2023 May 1;19(5):e1011358. doi: 10.1371/journal.ppat.1011358 (PMC10174524; doi:10.1371/journal.ppat.1011358)
Supplement: S1 Table — (PDF) [file ppat.1011358.s008.pdf]

S1 Table

Sl. No.	Compounds	Structure	Replicate #	Cell number (total)	Cell number (IAV-infected)	% IAV infection	Average infection	% IAV infection normalized to DMSO	% IAV infection w.r.t. DMSO	% Inhibition
1	DMSO		1	2677	623	23.27232	26.1841	88.88	100.0	0.0
			2	3051	867	28.41691		108.53		
			3	3019	811	26.8632		102.59		
2	D1	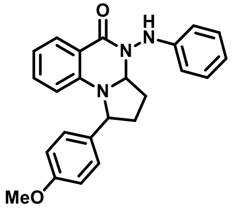	1	2539	526	20.71682	21.4158	79.12	81.8	18.2
			2	3112	702	22.55784		86.15		
			3	2961	621	20.97264		80.10		
3	D2	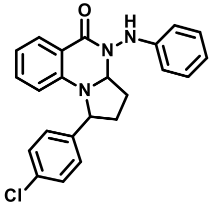	1	2930	429	14.64164	13.1458	55.92	50.2	49.8
			2	2933	354	12.06955		46.09		
			3	3426	436	12.72621		48.60		
4	D3	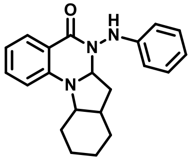	1	2961	650	21.95204	20.1788	83.84	77.1	22.9
			2	3089	505	16.34833		62.44		
			3	3220	716	22.23602		84.92		
5	D4	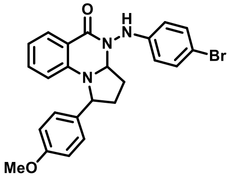	1	2809	553	19.68672	17.8444	75.19	68.1	31.9
			2	3334	592	17.75645		67.81		
			3	3381	544	16.08991		61.45		
6	D5	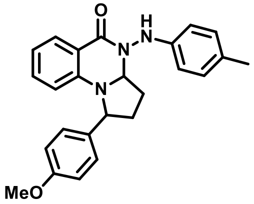	1	2729	561	20.55698	16.1643	78.51	61.7	38.3
			2	2373	258	10.87231		41.52		
			3	3024	516	17.06349		65.17		

7	D6	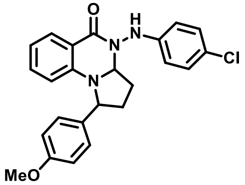	1	1528	150	9.816754	13.7294	37.49	52.4	47.6
			2	2263	343	15.15687		57.89		
			3	3133	508	16.21449		61.92		
8	D7	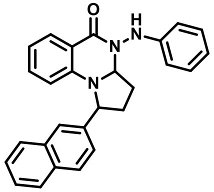	1	1106	77	6.962025	11.5961	26.59	44.3	55.7
			2	2204	341	15.47187		59.09		
			3	1975	244	12.35443		47.18		
9	**D8 (DPUD-1)**	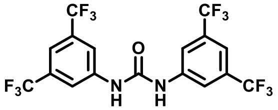	1	9038	9	0.09958	0.05655	0.38	**0.2**	**99.8**
			2	2854	2	0.070077		0.27		
			3	2772	0	0		0.00		
10	D9	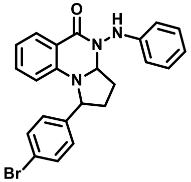	1	1292	259	20.04644	14.4757	76.56	55.3	44.7
			2	1660	190	11.44578		43.71		
			3	1902	227	11.93481		45.58		
11	D10	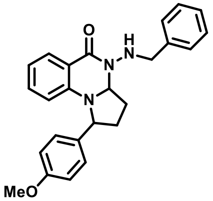	1	1603	198	12.35184	10.7604	47.17	41.1	58.9
			2	1685	175	10.38576		39.66		
			3	1446	138	9.543568		36.45		
12	D11	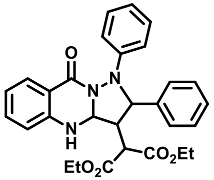	1	1689	200	11.84133	15.1781	45.22	58.0	42.0
			2	2163	362	16.73601		63.92		
			3	1952	331	16.95697		64.76		
13	D12	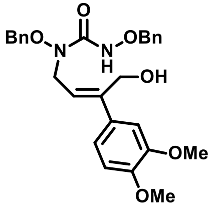	1	1696	124	7.311321	8.52454	27.92	32.6	67.4
			2	1668	156	9.352518		35.72		
			3	1807	161	8.909795		34.03		

14	D13	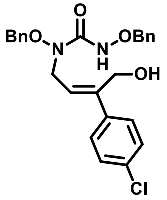	1	1201	57	4.746045	5.95927	18.13	22.8	77.2
			2	949	25	2.634352		10.06		
			3	3277	344	10.49741		40.09		
15	D14	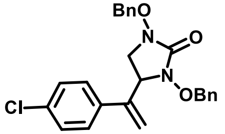	1	2223	241	10.84121	12.437	41.40	47.5	52.5
			2	2027	221	10.90281		41.64		
			3	1728	269	15.56713		59.45		
16	D15	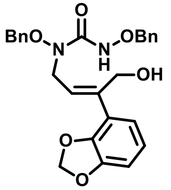	1	2710	461	17.01107	18.1088	64.97	69.2	30.8
			2	2637	495	18.77133		71.69		
			3	3214	596	18.54387		70.82		
17	D16	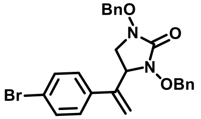	1	2312	330	14.27336	16.4182	54.51	62.7	37.3
			2	1978	380	19.21132		73.37		
			3	1877	296	15.76985		60.23		
18	D17	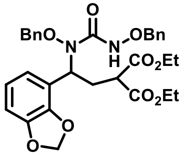	1	1766	221	12.51416	18.6137	47.79	71.1	28.9
			2	2253	489	21.70439		82.89		
			3	3057	661	21.62251		82.58		
19	D18	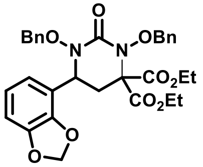	1	1421	137	9.641098	15.9253	36.82	60.8	39.2
			2	1738	231	13.29114		50.76		
			3	3357	834	24.84361		94.88		
20	D19	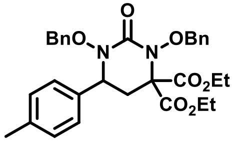	1	2010	435	21.64179	23.5086	82.65	89.8	10.2
			2	2370	565	23.83966		91.05		
			3	2252	564	25.0444		95.65		
21	D20	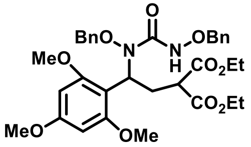	1	4483	414	9.234887	11.2607	35.27	43.0	57.0
			2	4014	500	12.4564		47.57		
			3	4185	506	12.0908		46.18		

22	D21	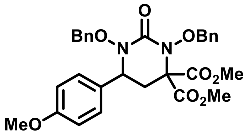	1	4860	558	11.48148	16.9499	43.85	64.7	35.3
			2	3861	653	16.91272		64.59		
			3	3714	834	22.45557		85.76		
23	D22	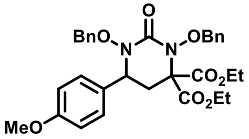	1	4251	582	13.6909	18.7821	52.29	71.7	28.3
			2	3720	760	20.43011		78.02		
			3	3703	823	22.22522		84.88		
24	D23	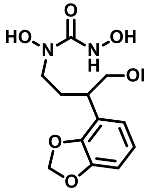	1	2729	466	17.07585	18.7963	65.21	71.8	28.2
			2	2932	491	16.74625		63.96		
			3	3483	786	22.56675		86.18		
25	D24	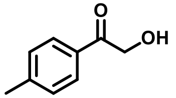	1	2584	456	17.64706	17.9987	67.40	68.7	31.3
			2	3071	523	17.03028		65.04		
			3	3406	658	19.31885		73.78		
26	D25	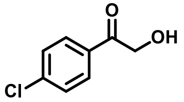	1	5841	337	5.76956	13.6647	22.03	52.2	47.8
			2	3576	543	15.18456		57.99		
			3	3508	703	20.03991		76.53		
27	D26	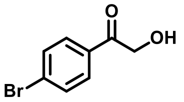	1	6452	322	4.990701	13.7362	19.06	52.5	47.5
			2	3051	553	18.1252		69.22		
			3	3366	609	18.09269		69.10		
29	D27	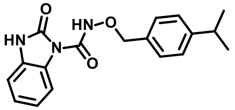	1	9392	186	1.980409	9.78049	7.56	37.4	62.6
			2	2592	419	16.16512		61.74		
			3	3144	352	11.19593		42.76		
30	D28	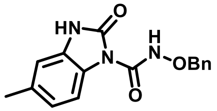	1	9212	208	2.257924	7.78541	8.62	29.7	70.3
			2	2866	366	12.77041		48.77		
			3	3062	255	8.32789		31.81		
31	NH_4Cl		1	3043	0	0	0.01314	0.00	0.1	99.9
			2	2980	0	0		0.00		
			3	2537	1	0.039417		0.15		
