# Supplementary material for: Identification of diphenylurea derivatives as novel endocytosis inhibitors that demonstrate broad-spectrum activity against SARS-CoV-2 and influenza A virus both in vitro and in vivo
Source: PLoS Pathog. 2023 May 1;19(5):e1011358. doi: 10.1371/journal.ppat.1011358 (PMC10174524; doi:10.1371/journal.ppat.1011358)
Supplement: S2 Table — (PDF) [file ppat.1011358.s009.pdf]

S2 Table

Compounds	Structure	Mol. formula (Mol. Weight)	IUPAC
**DPU**	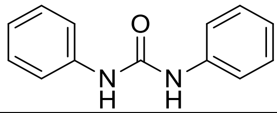	$C_{13}H_{12}N_2O$ (212.0950)	1,3-diphenylurea
**DPUD-1**	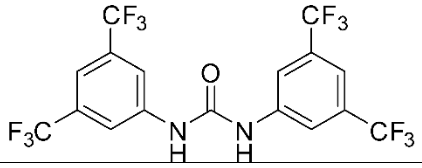	$C_{17}H_8F_{12}N_2O$ (484.0445)	1,3-bis(3,5-bis(trifluoromethyl)phenyl)urea
**DPUD-2**	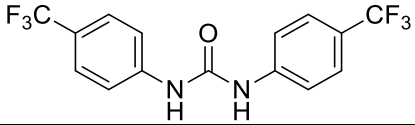	$C_{15}H_{10}F_6N_2O$ (348.0697)	1,3-bis(4-(trifluoromethyl)phenyl)urea
**DPUD-3**	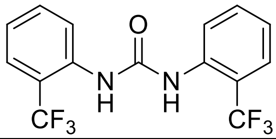	$C_{15}H_{10}F_6N_2O$ (348.0697)	1,3-bis(2-(trifluoromethyl)phenyl)urea
**DPUD-4**	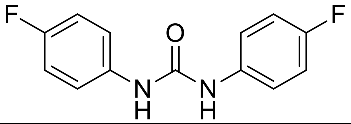	$C_{13}H_{10}F_2N_2O$ (248.0761)	1,3-bis(4-fluorophenyl)urea
**DPUD-5**	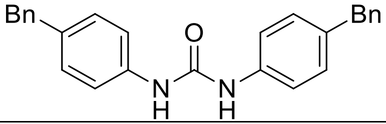	$C_{27}H_{24}N_2O$ (392.1889)	1,3-bis(4-benzylphenyl)urea
**DPUD-6**	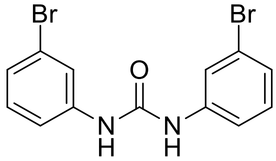	$C_{13}H_{10}Br_2N_2O$ (367.9160)	1,3-bis(3-bromophenyl)urea
**DPUD-7**	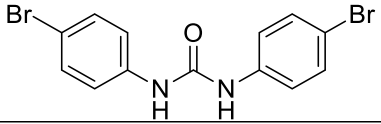	$C_{13}H_{10}Br_2N_2O$ (367.9160)	1,3-bis(4-bromophenyl)urea
**DPUD-8**	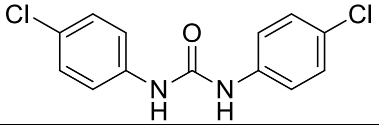	$C_{13}H_{10}Cl_2N_2O$ (280.0170)	1,3-bis(4-chlorophenyl)urea
**DPUD-9**	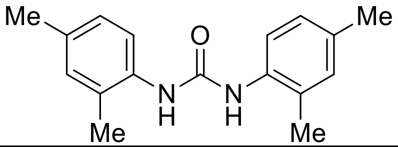	$C_{17}H_{20}N_2O$ (268.1576)	1,3-bis(2,4-dimethylphenyl)urea
**DPUD-10**	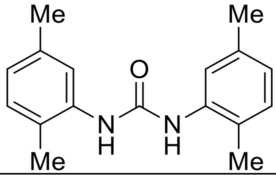	$C_{17}H_{20}N_2O$ (268.1576)	1,3-bis(2,5-dimethylphenyl)urea
**DPUD-11**	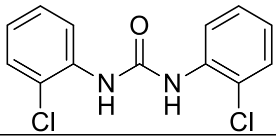	$C_{13}H_{10}Cl_2N_2O$ (280.0170)	1,3-bis(2-chlorophenyl)urea

**DPUD-12**	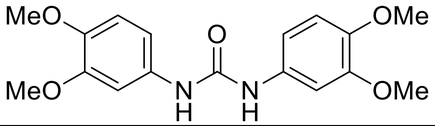	$C_{17}H_{20}N_2O_5$ (332.1372)	1,3-bis(3,4-dimethoxyphenyl)urea
**DPUD-13**	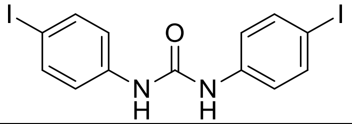	$C_{13}H_{10}I_2N_2O$ (463.8882)	1,3-bis(4-iodophenyl)urea
**DPUD-14**	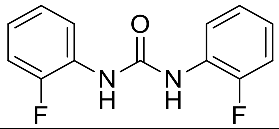	$C_{13}H_{10}F_2N_2O$ (248.0761)	1,3-bis(2-fluorophenyl)urea
**DPUD-15**	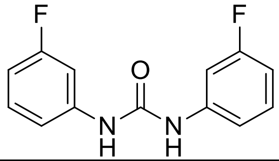	$C_{13}H_{10}F_2N_2O$ (248.0761)	1,3-bis(3-fluorophenyl)urea
**DPUD-16**	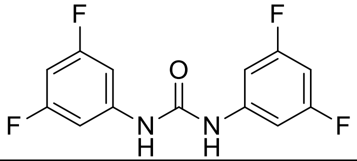	$C_{13}H_8F_4N_2O$ (284.0573)	1,3-bis(3,5-difluorophenyl)urea
**DPUD-17**	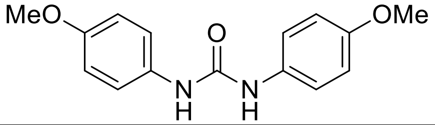	$C_{15}H_{16}N_2O_3$ (272.1161)	1,3-bis(4-methoxyphenyl)urea
**DPUD-18**	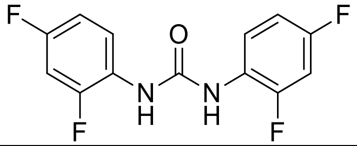	$C_{13}H_8F_4N_2O$ (284.0573)	1,3-bis(2,4-difluorophenyl)urea
**DPUD-19**	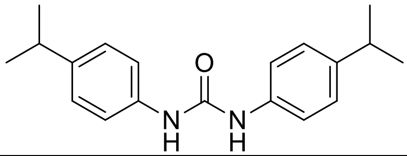	$C_{19}H_{24}N_2O$ (296.1889)	1,3-bis(4-isopropylphenyl)urea
**DPUD-20**	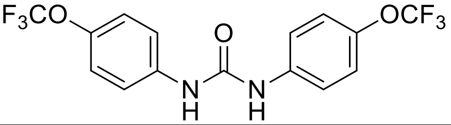	$C_{15}H_{10}F_6N_2O_3$ (380.0596)	1,3-bis(4-(trifluoromethoxy)phenyl)urea
**DPUD-21**	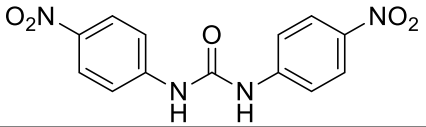	$C_{13}H_{10}N_4O_5$ (302.0651)	1,3-bis(4-nitrophenyl)urea
**DPUD-22**	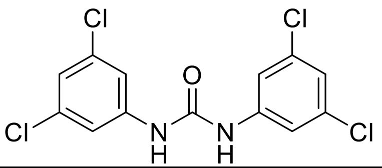	$C_{13}H_8Cl_4N_2O$ (347.9391)	1,3-bis(3,5-dichlorophenyl)urea
**DPUD-23**	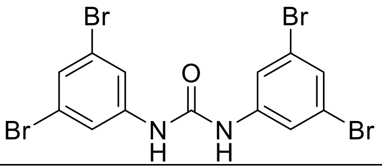	$C_{13}H_8Br_4N_2O$ (523.7370)	1,3-bis(3,5-dibromophenyl)urea
