# Supplementary material for: Identification of diphenylurea derivatives as novel endocytosis inhibitors that demonstrate broad-spectrum activity against SARS-CoV-2 and influenza A virus both in vitro and in vivo
Source: PLoS Pathog. 2023 May 1;19(5):e1011358. doi: 10.1371/journal.ppat.1011358 (PMC10174524; doi:10.1371/journal.ppat.1011358)

S3 Table

1,3-bis(3,5-bis(trifluoromethyl)phenyl)urea (DPUD-1)

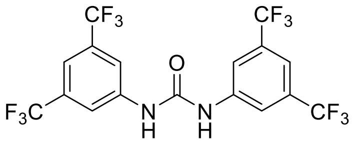

DPUD-1

^{1}H NMR (400 MHz, DMSO): δ 9.75 (s, 2H), 8.19 (s, 4H), 7.72 (s, 2H).

1,3-bis(4-(trifluoromethyl)phenyl)urea (DPUD-2)

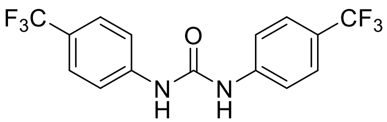

DPUD-2

^{1}H NMR (400 MHz, CHLOROFORM-D): δ 7.59-7.51 (m, 8H), 6.91 (s, 2H).

1,3-bis(2-(trifluoromethyl)phenyl)urea (DPUD-3)

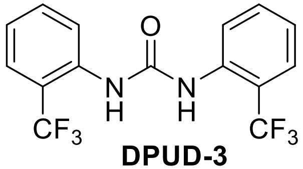

DPUD-3

^{1}H NMR (400 MHz, CHLOROFORM-D): δ 8.68 (s, 2H), 7.77 (d, *J* = 8.2 Hz, 2H), 7.66 (d, *J* = 7.9 Hz, 2H), 7.60 (t, *J* = 7.8 Hz, 2H), 7.28 (t, *J* = 7.6 Hz, 2H).

1,3-bis(4-fluorophenyl)urea (DPUD-4)

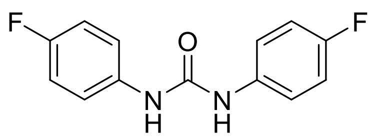

DPUD-4

^{1}H NMR (400 MHz, DMSO-d^6): δ 8.71 (s, 2H), 7.42-7.38 (m, 4H), 7.17-7.04 (m, 4H).

1,3-bis(4-benzylphenyl)urea (DPUD-5)

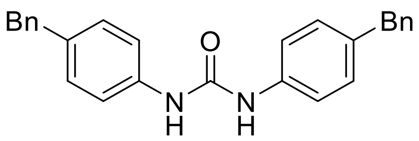

DPUD-5

^{1}H NMR (400 MHz, DMSO-D6) δ 8.51 (s, 2H), 7.30 (d, *J* = 8.5 Hz, 4H), 7.23 (t, *J* = 7.4 Hz, 4H), 7.17-7.11 (m, 6H), 7.07 (d, *J* = 8.5 Hz, 4H), 3.82 (s, 4H).

1,3-bis(3-bromophenyl)urea (DPUD-6)

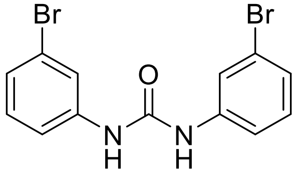

DPUD-6

^{1}H NMR (400 MHz, DMSO-D6): δ 8.91 (s, 2H), 7.80 (t, *J* = 1.9 Hz, 2H), 7.29-7.25 (m, 2H), 7.20 (t, *J* = 8.0 Hz, 2H), 7.12 (d, *J* = 7.9 Hz, 2H).

1,3-bis(4-bromophenyl)urea (DPUD-7)

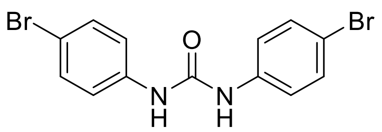

DPUD-7

^{1}H NMR (400 MHz, DMSO-D6): δ 8.85 (s, 2H), 7.42-7.36 (m, 8H).

1,3-bis(4-chlorophenyl)urea (DPUD-8)

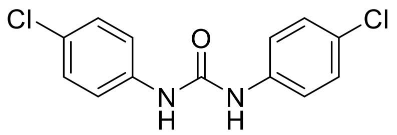

DPUD-8

^{1}H NMR (400 MHz, DMSO-D6): δ 8.81 (s, 2H), 7.45-7.41 (m, 4H), 7.30-7.26 (m, 4H).

1,3-bis(2,4-dimethylphenyl)urea)urea (DPUD-9)

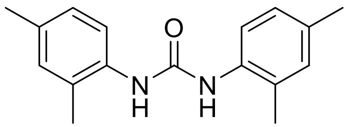

DPUD-9

^{1}H NMR (400 MHz, DMSO-D6): δ 8.13 (s, 2H), 7.59 (s, 2H), 6.97-6.83 (m, 4H), 2.17 (s, 12H).

1,3-bis(2,5-dimethylphenyl)urea (DPUD-10)

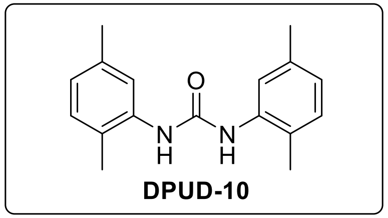

^{1}H NMR (400 MHz, DMSO-D6): δ 8.13 (s, 2H), 7.62 (s, 2H), 7.00 (d, *J* = 7.4 Hz, 2H), 6.71 (d, *J* = 7.9 Hz, 2H), 2.20 (s, 3H), 2.16 (s, 3H).

1,3-bis(2-chlorophenyl)urea (DPUD-11)

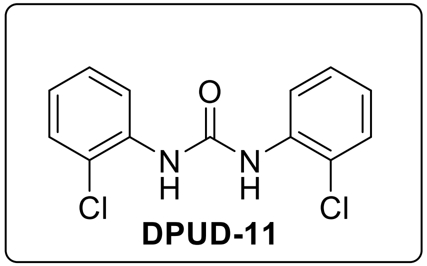

^{1}H NMR (400 MHz, DMSO-D6): δ 9.01 (s, 2H), 8.03 (dd, *J* = 8.2, 1.0 Hz, 2H), 7.43 (dd, *J* = 8.0, 1.1 Hz, 2H), 7.28-7.24 (m, 2H), 7.04-7.00 (m, 2H).

1,3-bis(3,4-dimethoxyphenyl)urea (DPUD-12)

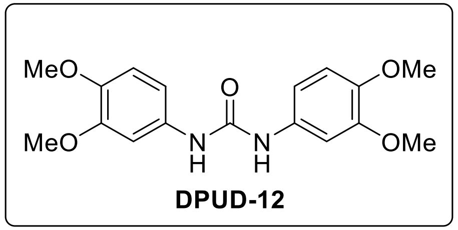

^{1}H NMR (400 MHz, DMSO-D6): δ 8.41 (s, 2H), 7.14 (s, 2H), 6.81 (s, 2H), 3.69 (s, 6H), 3.66 (s, 6H).

1,3-bis(4-iodophenyl)urea (DPUD-13)

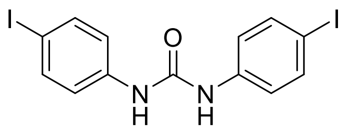

DPUD-13

^{1}H NMR (400 MHz, DMSO-D6): δ 8.82 (s, 2H), 7.55 (d, *J* = 8.6 Hz, 4H), 7.25 (d, *J* = 8.6 Hz, 4H).

1,3-bis(2-fluorophenyl)urea (DPUD-14)

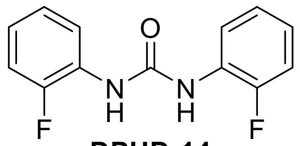

DPUD-14

^{1}H NMR (400 MHz, DMSO-D6): δ 9.01 (s, 2H), 8.14 (t, *J* = 8.2 Hz, 2H), 7.23-7.18 (m, 2H), 7.10 (t, *J* = 7.6 Hz, 2H), 7.00-6.95(m, 2H).

1,3-bis(3-fluorophenyl)urea (DPUD-15)

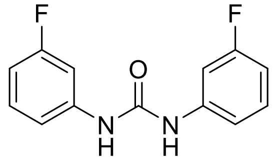

DPUD-15

^{1}H NMR (400 MHz, DMSO-D6): δ 8.94 (s, 2H), 7.44 (dd, *J* = 12.0, 1.8 Hz, 2H), 7.31-7.23 (m, 2H), 7.11-7.06 (m, 2H), 6.79-6.72 (m, 2H).

1,3-bis(3,5-difluorophenyl)urea (DPUD-16)

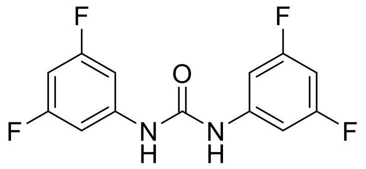

DPUD-16

^{1}H NMR (400 MHz, DMSO-D6): δ 9.29 (s, 1H), 7.18-7.11 (m, 4H), 6.83-6.77 (m, 2H).

1,3-bis(4-methoxyphenyl)urea (DPUD-17)

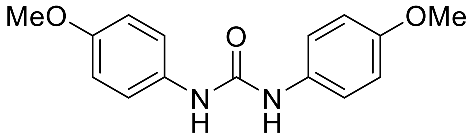

DPUD-17

^{1}H NMR (400 MHz, DMSO-D6): δ 8.45 (s, 2H), 7.32–7.27 (m, 4H), 6.84–6.79 (m, 4H), 3.66 (s, 6H).

1,3-bis(2,4-difluorophenyl)urea (DPUD-18)

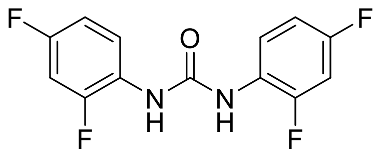

DPUD-18

^{1}H NMR (400 MHz, DMSO-D6): δ 8.96 (s, 2H), 8.10–7.98 (m, 2H), 7.31–7.20 (m, 2H), 7.00-6.97 (m, 2H).

1,3-bis(4-isopropylphenyl)urea (DPUD-19)

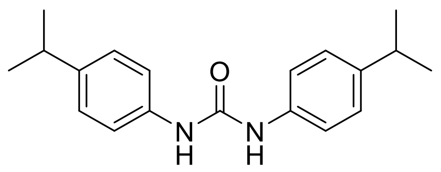

DPUD-19

^{1}H NMR (400 MHz, DMSO-D6): δ 8.49 (s, 2H), 7.34–7.27 (m, 44H), 7.09 (d, *J* = 8.4 Hz, 2H), 2.83-2.72 (m, 2H), 1.13 (d, *J* = 6.9 Hz, 12H).

1,3-bis(4-(trifluoromethoxy)phenyl)urea (DPUD-20)

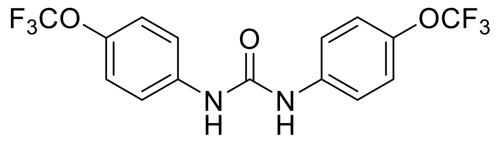

DPUD-20

^{1}H NMR (400 MHz, DMSO-D6) δ 8.99 (s, 2H), 7.5 –7.47 (m, 4H), 7.24 (d, *J* = 7.4 Hz, 4H).

1,3-bis(4-nitrophenyl)urea (DPUD-21)

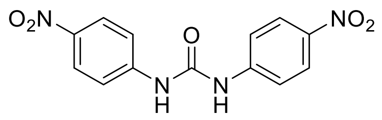

DPUD-21

^{1}H NMR (400 MHz, DMSO-D6): δ 9.62 (s, 2H), 8.17 (d, *J* = 8.2 Hz, 4H), 7.67 (d, *J* = 7.9 Hz, 4H).

1,3-bis(3,5-dichlorophenyl)urea (DPUD-22)

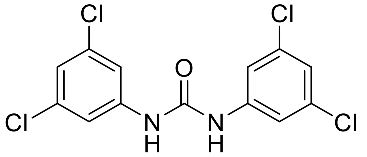

DPUD-22

^{1}H NMR (400 MHz, DMSO-D6): δ 9.23 (s, 2H), 7.50 (d, *J* = 1.8 Hz, 4H), 7.16 (dd, *J* = 2.2, 1.3 Hz, 2H).

1,3-bis(3,5-dibromophenyl)urea (DPUD-23)

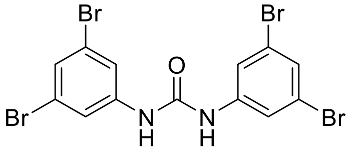

DPUD-23

^{1}H NMR (400 MHz, DMSO-D6): δ 9.29 (s, 2H), 7.19–7.11 (m, 4H), 6.79 (ddd, *J* = 9.2, 7.1, 2.3 Hz, 2H).

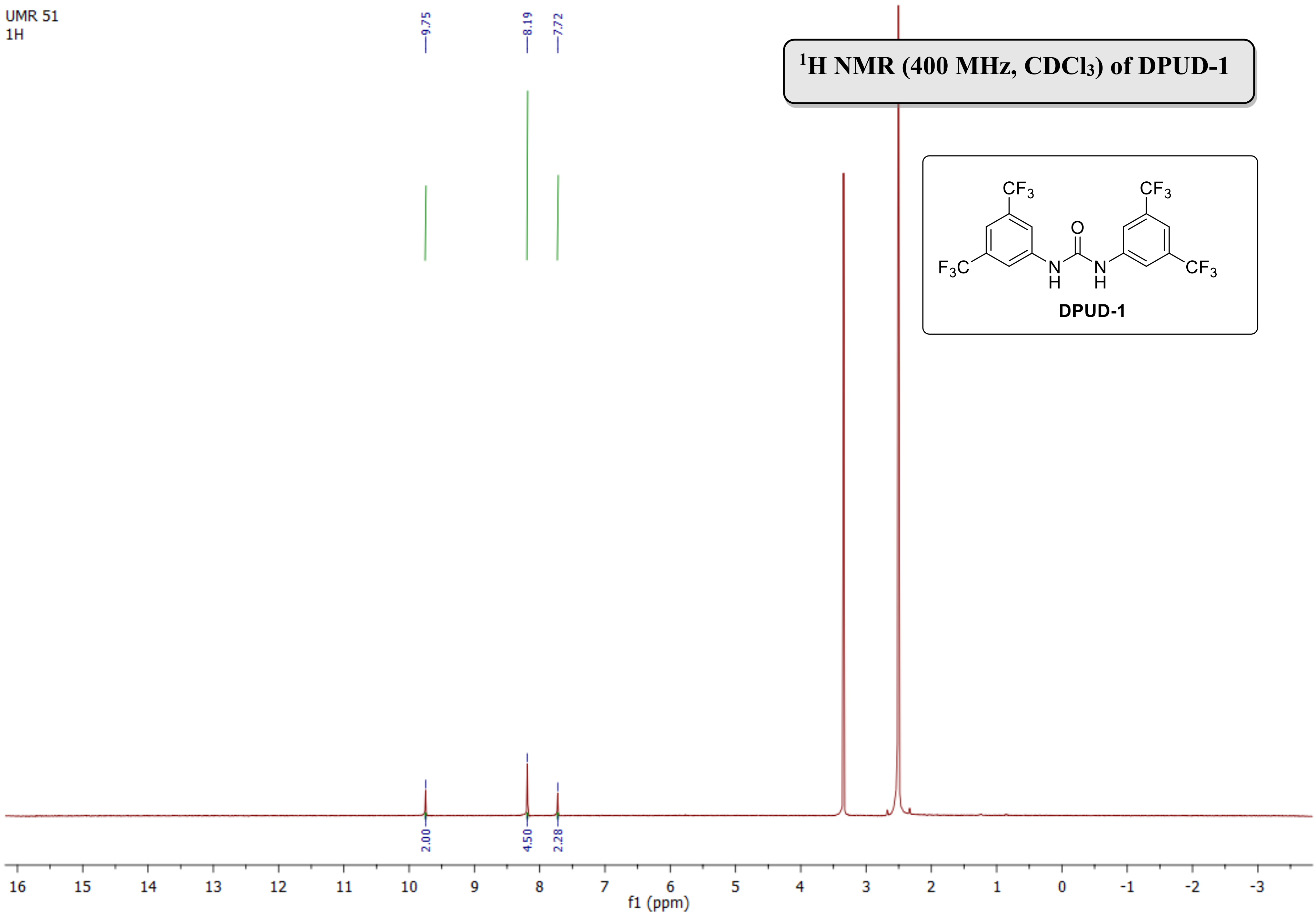
UMR 51
1H
9.75
8.19
7.72
¹H NMR (400 MHz, CDCl₃) of DPUD-1
CF3
CF3
O
F3C
N
H
N
H
CF3
DPUD-1
2.00
4.50
2.28
16
15
14
13
12
11
10
9
8
7
6
5
4
3
2
1
0
-1
-2
-3
f1 (ppm)

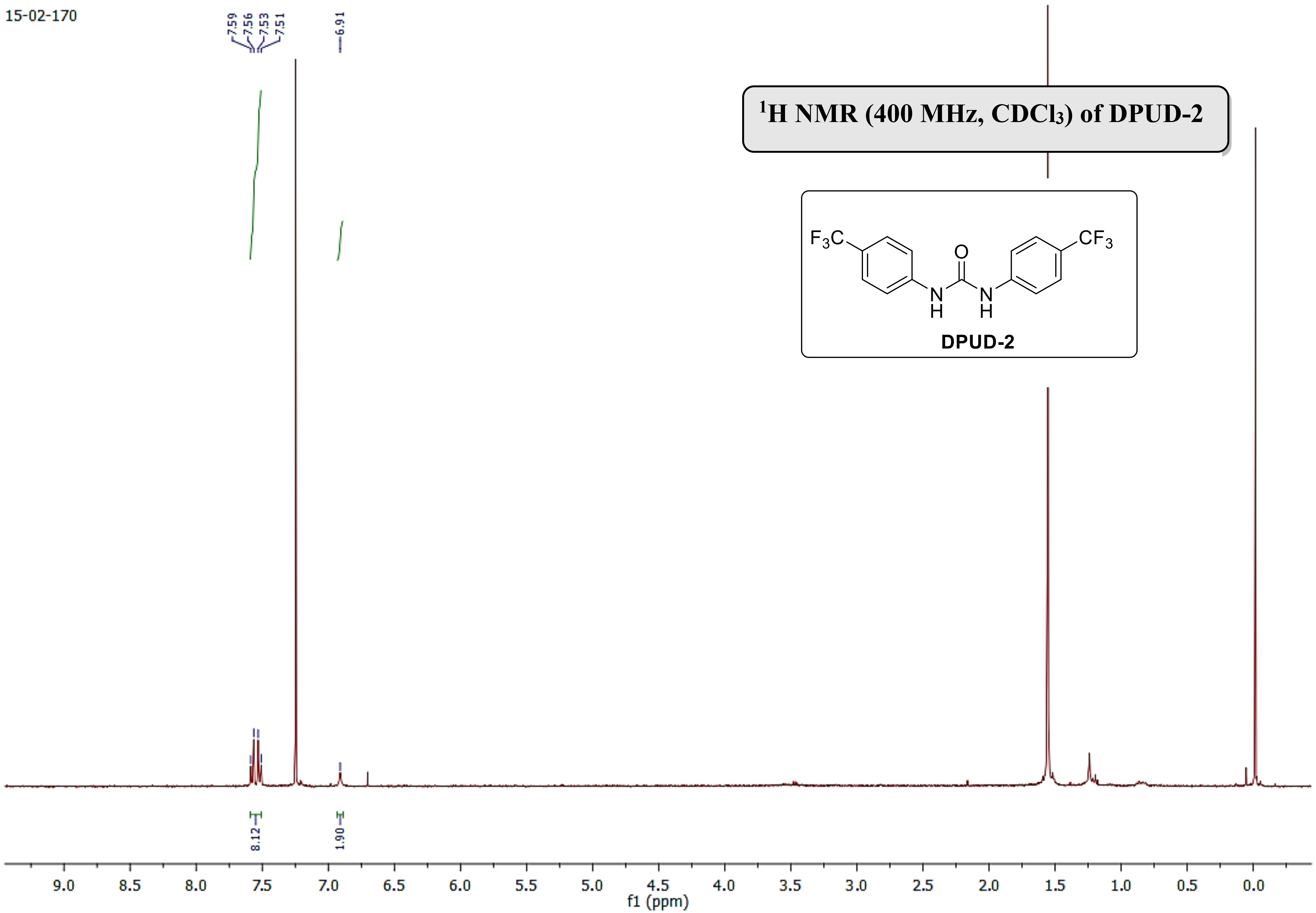
15-02-170
7.59
7.56
7.53
7.51
6.91
^{1}H NMR (400 MHz, CDCl$_3$) of DPUD-2
F3C
CF3
O
N
H
N
H
DPUD-2
8.12
1.90
9.0
8.5
8.0
7.5
7.0
6.5
6.0
5.5
5.0
4.5
4.0
3.5
3.0
2.5
2.0
1.5
1.0
0.5
0.0
f1 (ppm)

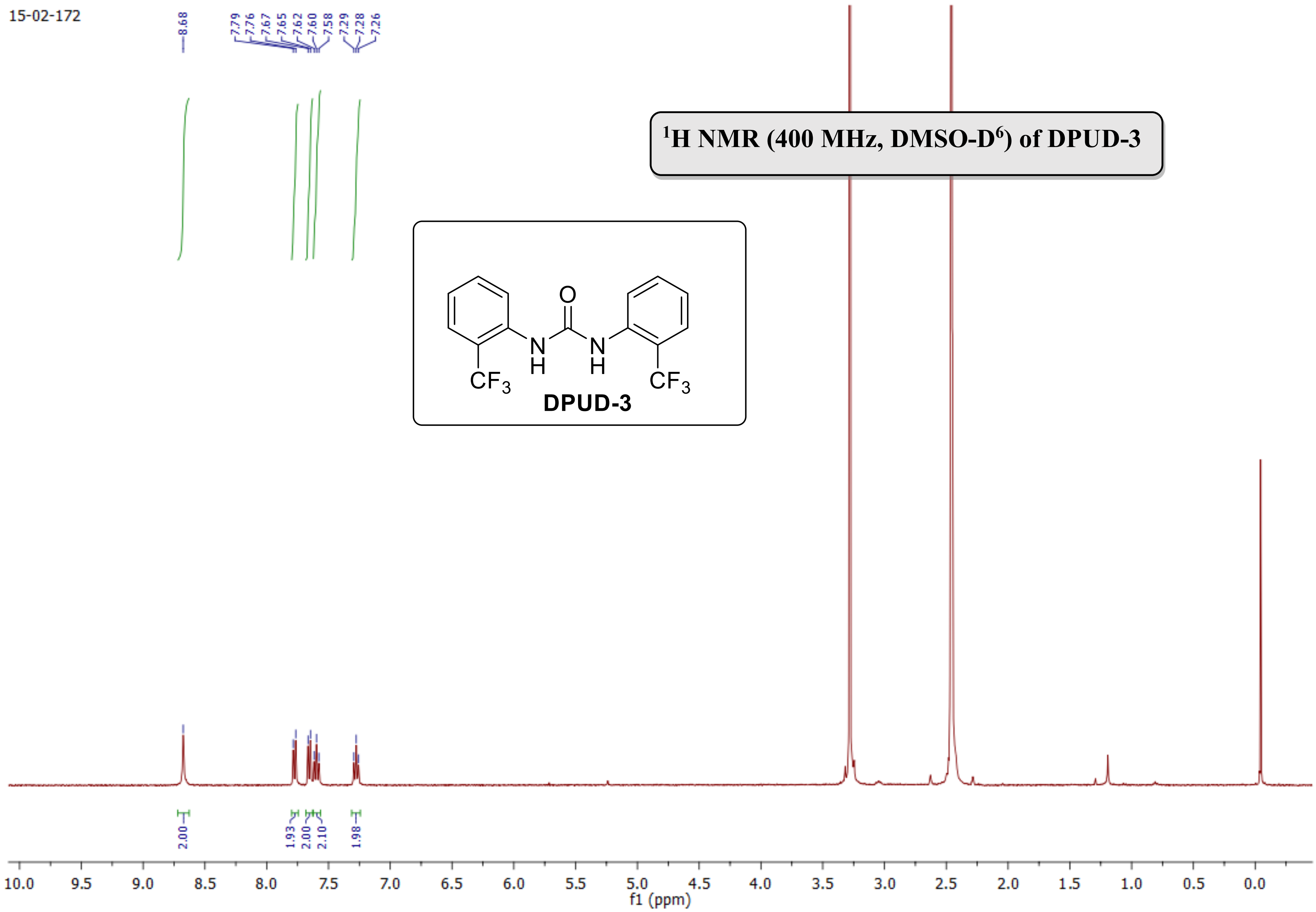
15-02-172
8.68
7.79
7.76
7.67
7.65
7.62
7.60
7.58
7.29
7.28
7.26
1H NMR (400 MHz, DMSO-D6) of DPUD-3
O
N
H
N
H
CF3
CF3
DPUD-3
2.00
1.93
2.00
2.10
1.98
10.0
9.5
9.0
8.5
8.0
7.5
7.0
6.5
6.0
5.5
5.0
4.5
4.0
3.5
3.0
2.5
2.0
1.5
1.0
0.5
0.0
f1 (ppm)

^1H NMR (400 MHz, DMSO-D^6) of DPUD-4

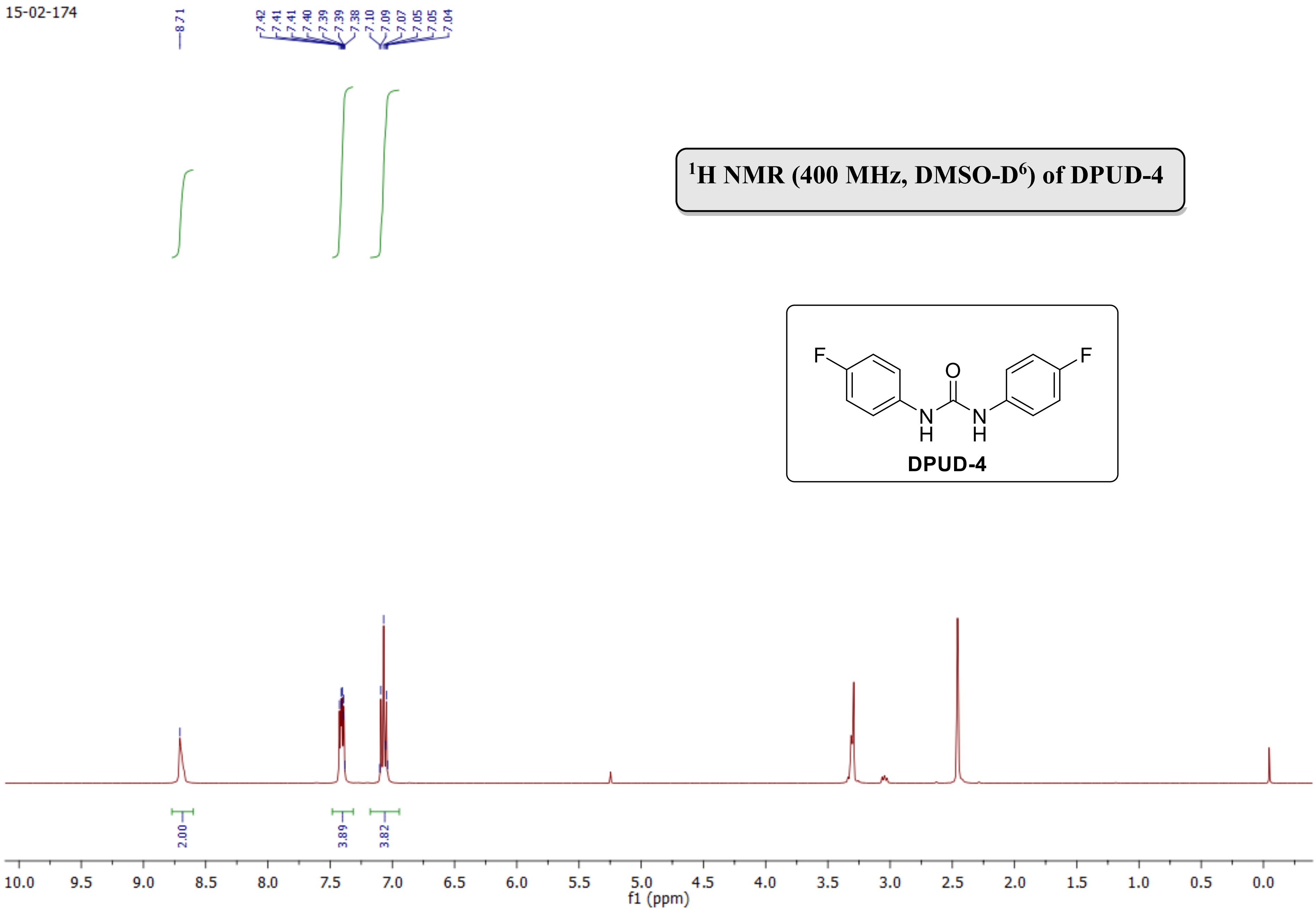

DPUD-4

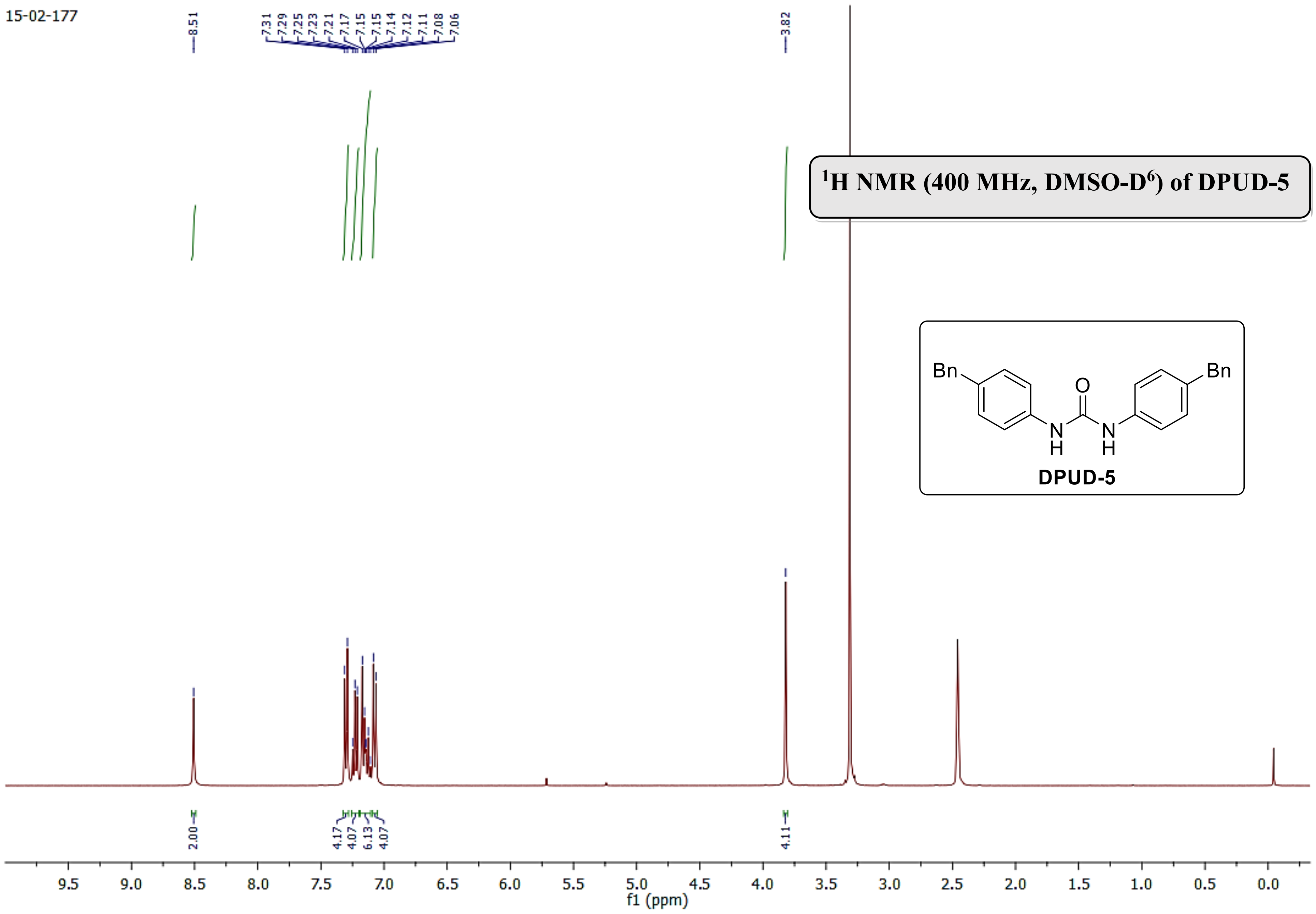

15-02-177
8.51
7.31 7.29 7.25 7.23 7.21 7.17 7.15 7.15 7.14 7.12 7.11 7.08 7.06
3.82
1H NMR (400 MHz, DMSO-D6) of DPUD-5
Bn
O
N H
DPUD-5
2.00
4.17 4.07 6.13 4.07
4.11
9.5 9.0 8.5 8.0 7.5 7.0 6.5 6.0 5.5 5.0 4.5 4.0 3.5 3.0 2.5 2.0 1.5 1.0 0.5 0.0
f1 (ppm)

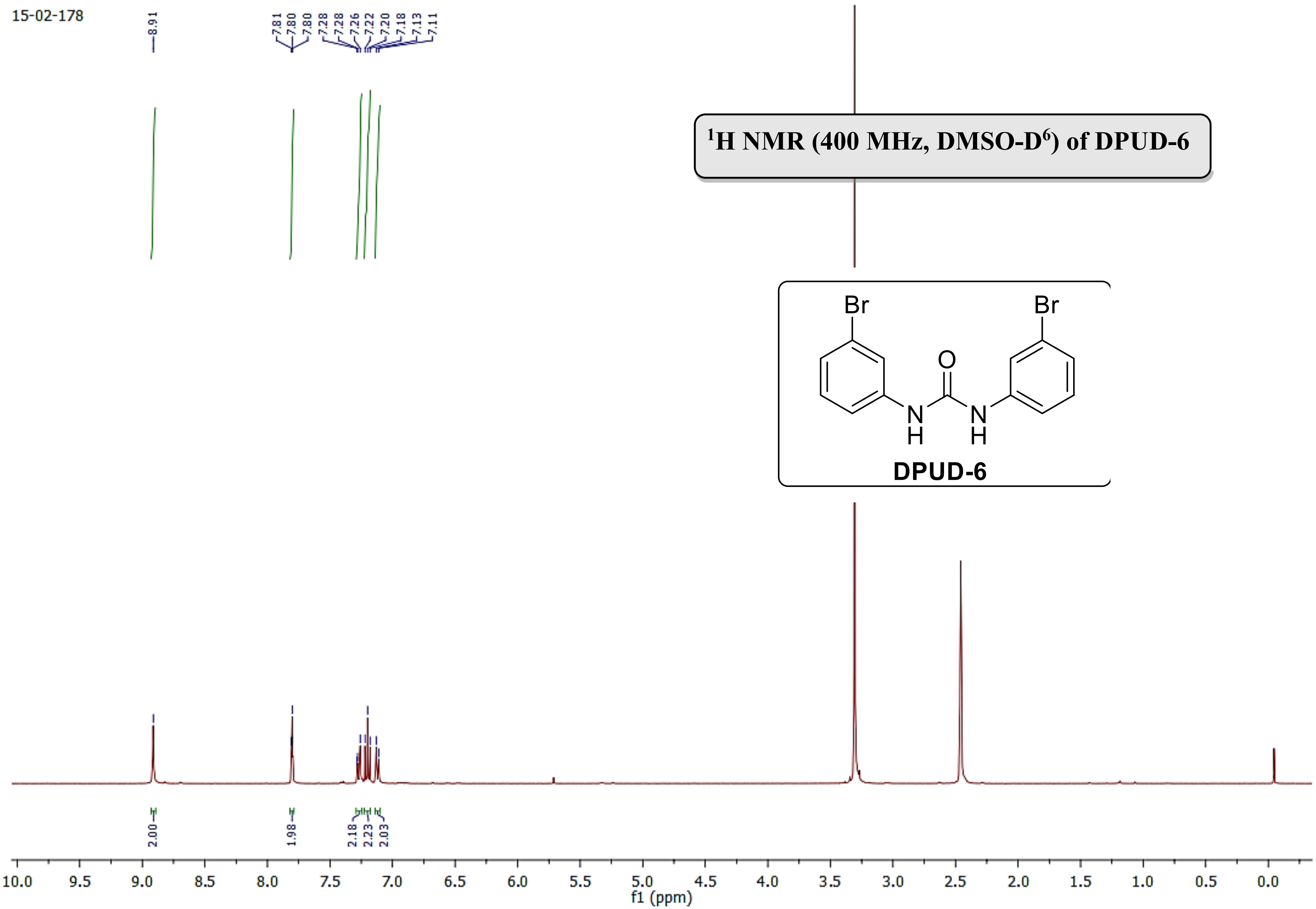
15-02-178
8.91
7.81
7.80
7.80
7.28
7.28
7.26
7.22
7.20
7.18
7.13
7.11
^{1}H NMR (400 MHz, DMSO-D^{6}) of DPUD-6
Br
Br
O
N
H
N
H
DPUD-6
2.00
1.98
2.18
2.23
2.03
10.0
9.5
9.0
8.5
8.0
7.5
7.0
6.5
6.0
5.5
5.0
4.5
4.0
3.5
3.0
2.5
2.0
1.5
1.0
0.5
0.0
f1 (ppm)

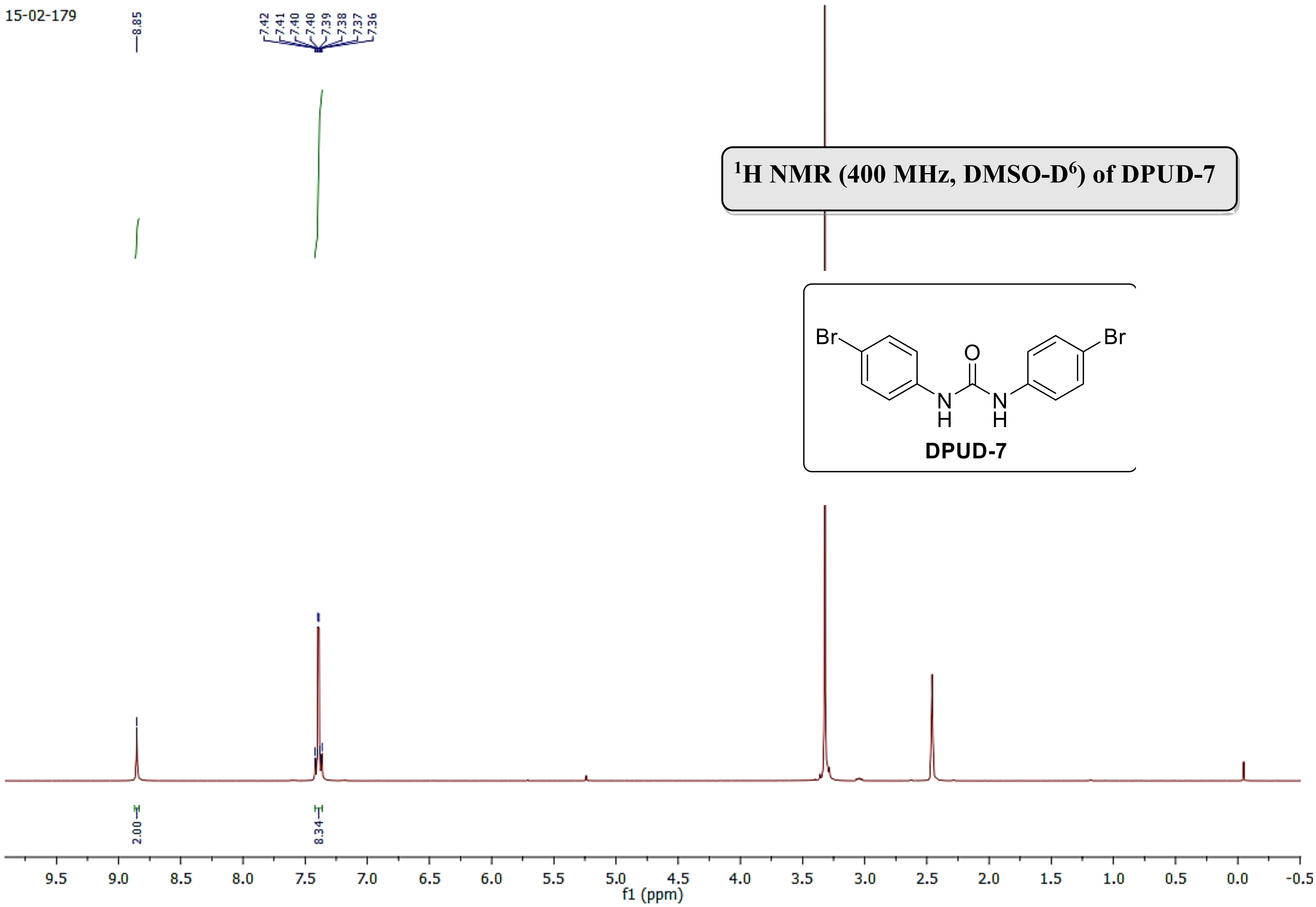
15-02-179
8.85
7.42
7.41
7.40
7.40
7.39
7.38
7.37
7.36
¹H NMR (400 MHz, DMSO-D⁶) of DPUD-7
Br
O
Br
N
H
N
H
DPUD-7
2.00
8.34
9.5
9.0
8.5
8.0
7.5
7.0
6.5
6.0
5.5
5.0
4.5
4.0
3.5
3.0
2.5
2.0
1.5
1.0
0.5
0.0
-0.5
f1 (ppm)

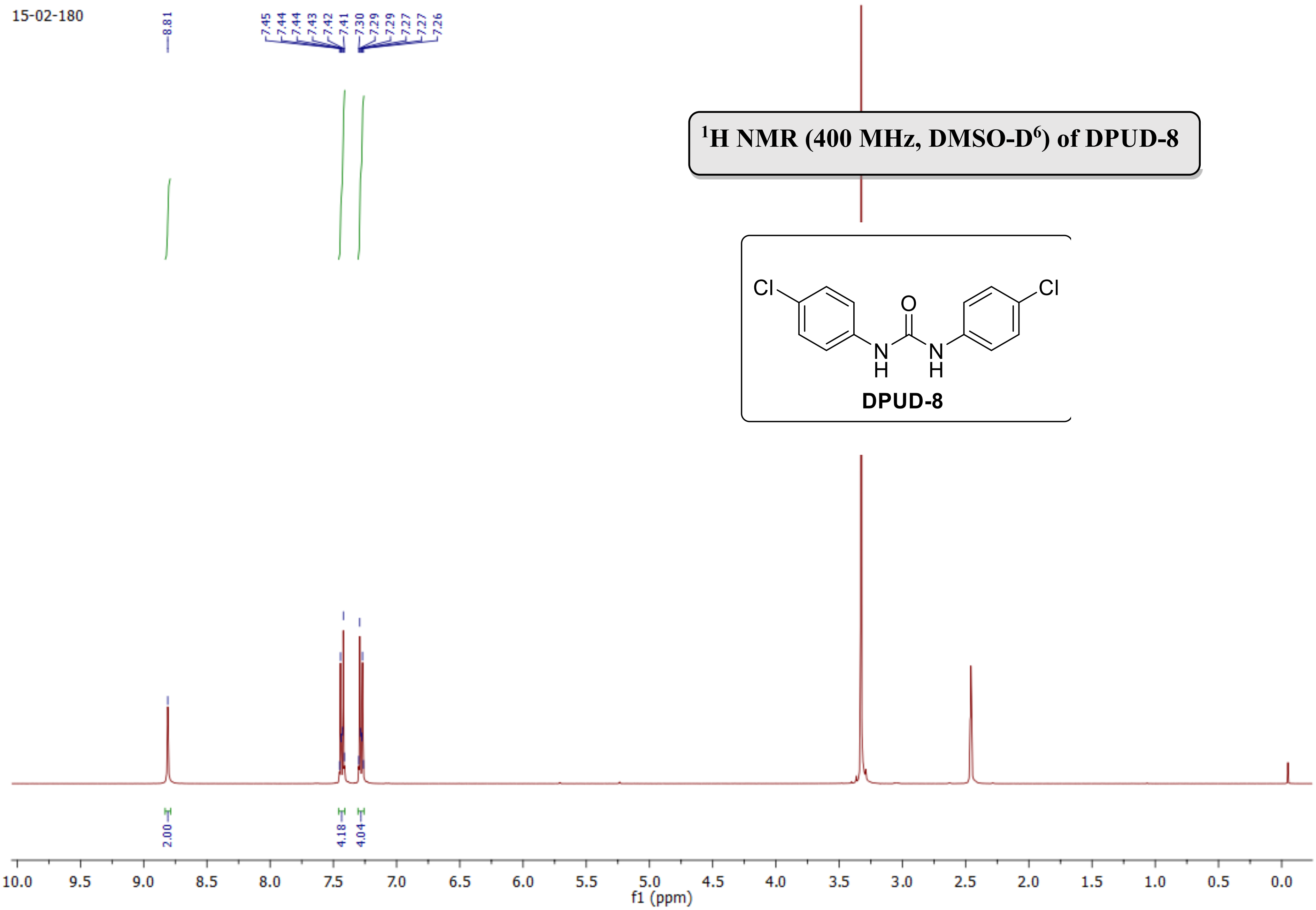
15-02-180
8.81
7.45
7.44
7.44
7.43
7.42
7.41
7.30
7.29
7.29
7.27
7.27
7.26
1H NMR (400 MHz, DMSO-D6) of DPUD-8
Cl
O
N
H
N
H
Cl
DPUD-8
2.00
4.18
4.04
10.0
9.5
9.0
8.5
8.0
7.5
7.0
6.5
6.0
5.5
5.0
4.5
4.0
3.5
3.0
2.5
2.0
1.5
1.0
0.5
0.0
f1 (ppm)

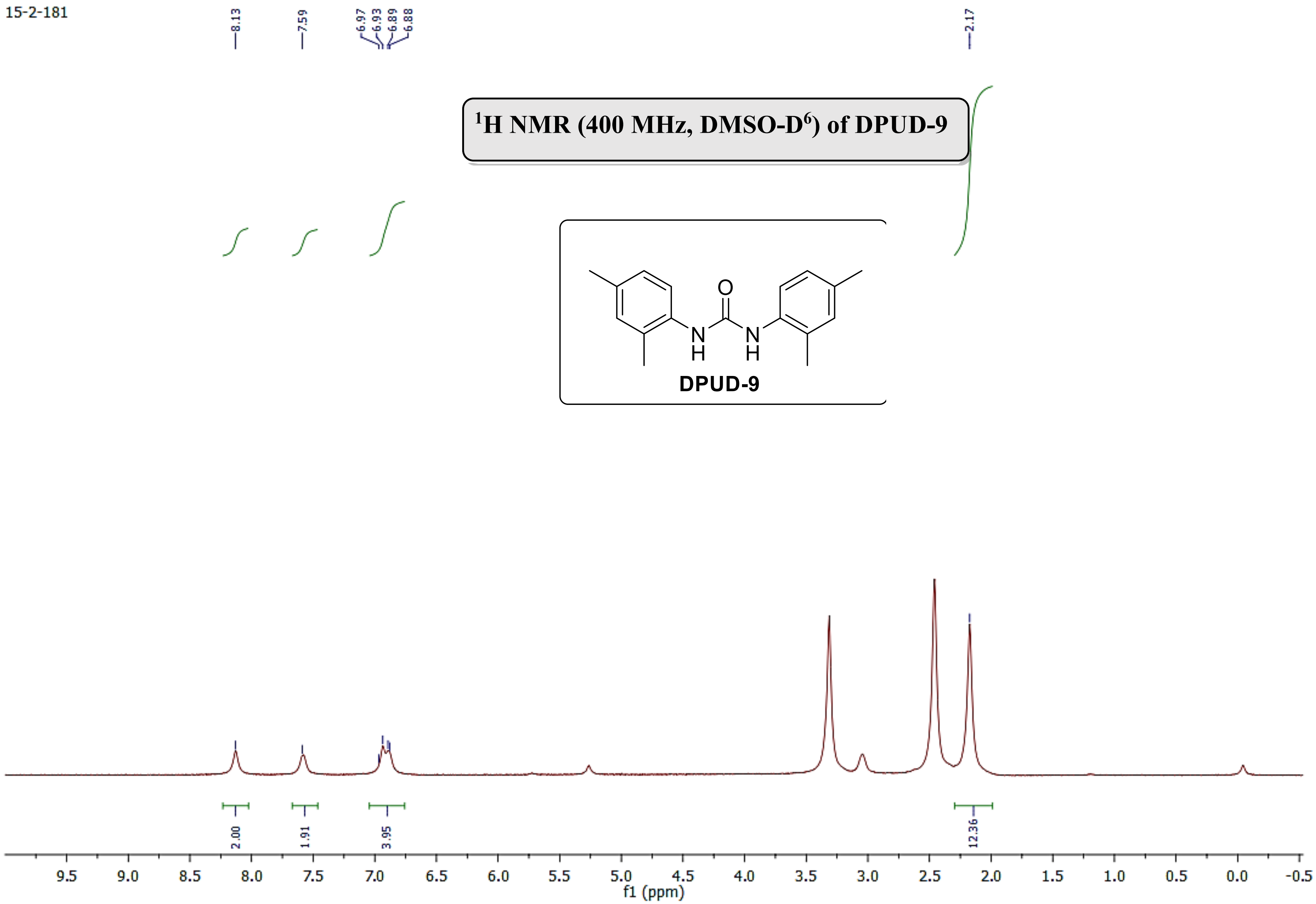
15-2-181
8.13
7.59
6.97
6.93
6.89
6.88
2.17
¹H NMR (400 MHz, DMSO-D⁶) of DPUD-9
O
N
H
N
H
DPUD-9
2.00
1.91
3.95
12.36
9.5 9.0 8.5 8.0 7.5 7.0 6.5 6.0 5.5 5.0 4.5 4.0 3.5 3.0 2.5 2.0 1.5 1.0 0.5 0.0 -0.5
f1 (ppm)

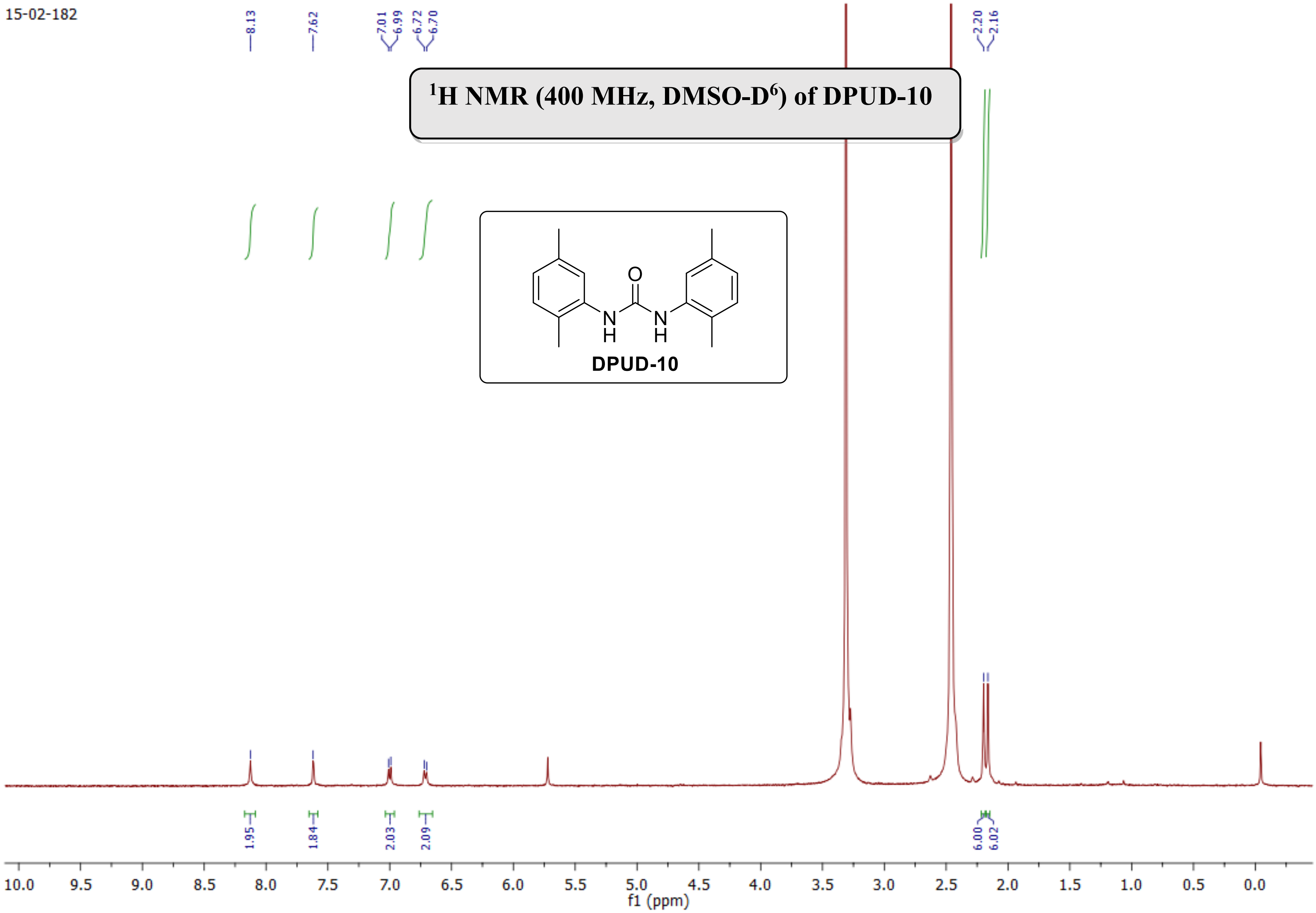
15-02-182
8.13
7.62
7.01
6.99
6.72
6.70
2.20
2.16
¹H NMR (400 MHz, DMSO-D⁶) of DPUD-10
O
N
H
N
H
DPUD-10
1.95
1.84
2.03
2.09
6.00
6.02
10.0
9.5
9.0
8.5
8.0
7.5
7.0
6.5
6.0
5.5
5.0
4.5
4.0
3.5
3.0
2.5
2.0
1.5
1.0
0.5
0.0
f1 (ppm)

^{1}H NMR (400 MHz, DMSO-D^{6}) of DPUD-11

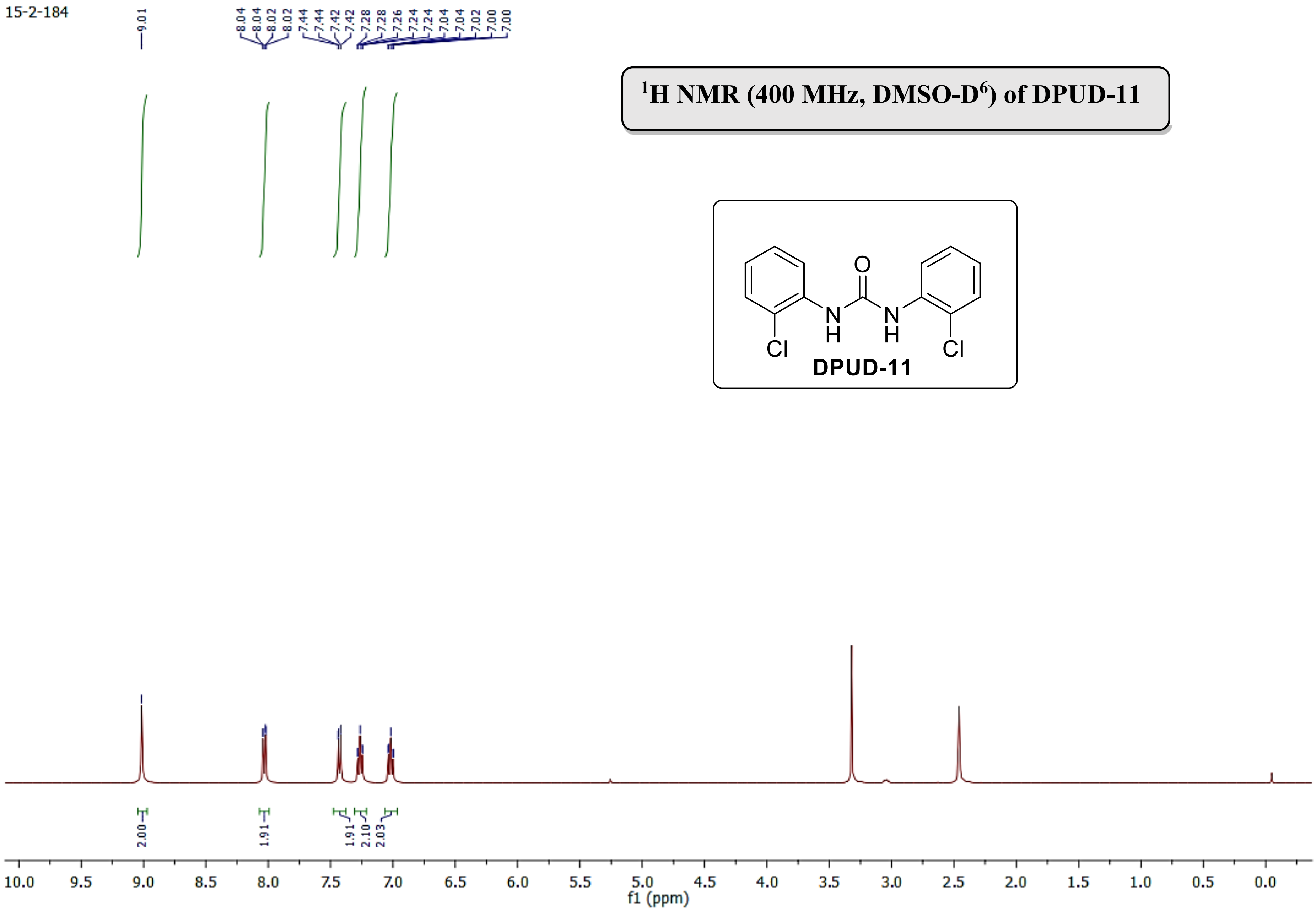
DPUD-11

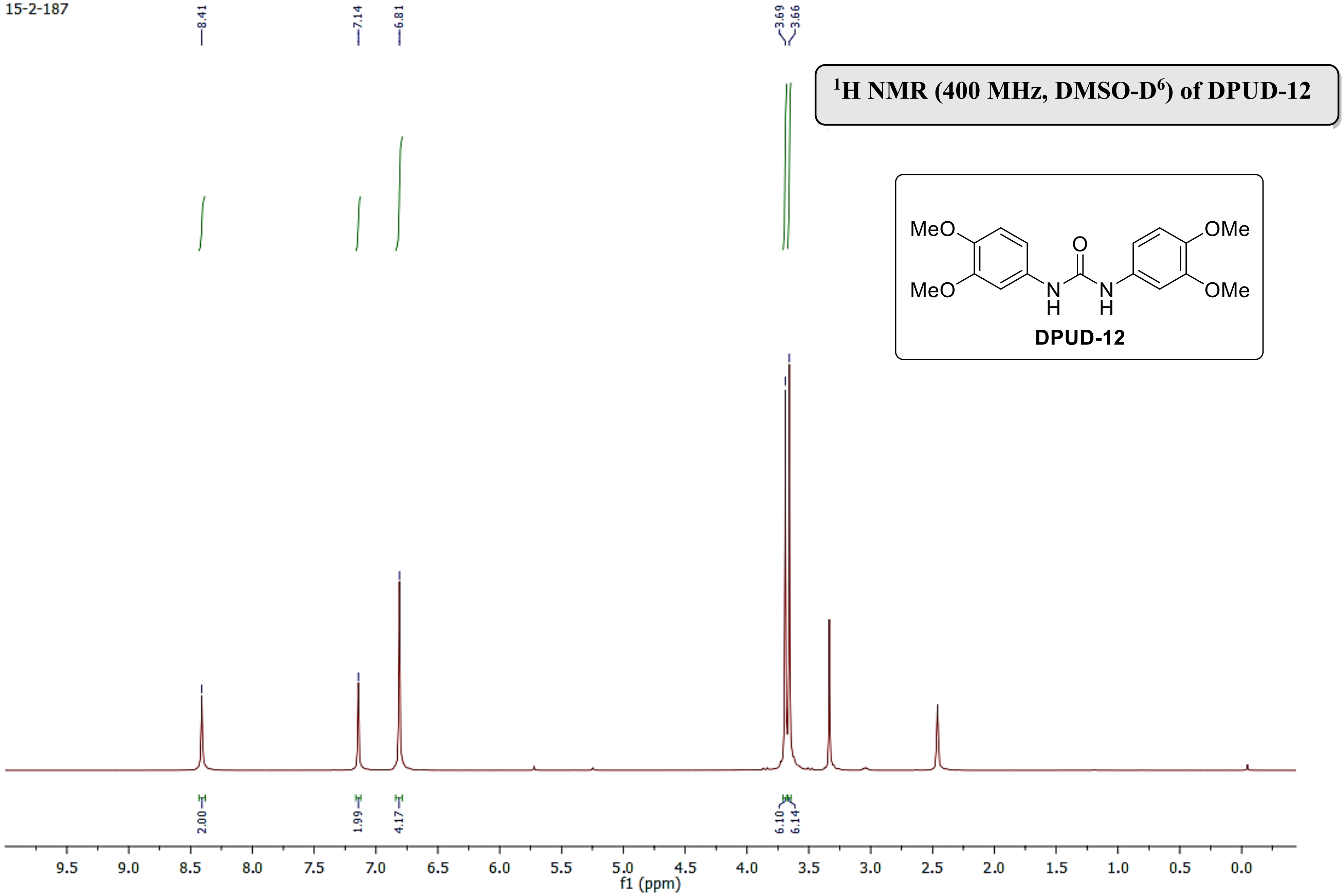
15-2-187
8.41
7.14
6.81
3.69
3.66
1H NMR (400 MHz, DMSO-D6) of DPUD-12
MeO
MeO
O
N
H
N
H
OMe
OMe
DPUD-12
2.00
1.99
4.17
6.10
6.14
9.5
9.0
8.5
8.0
7.5
7.0
6.5
6.0
5.5
5.0
4.5
4.0
3.5
3.0
2.5
2.0
1.5
1.0
0.5
0.0
f1 (ppm)

^{1}H NMR (400 MHz, DMSO-D^6) of DPUD-13

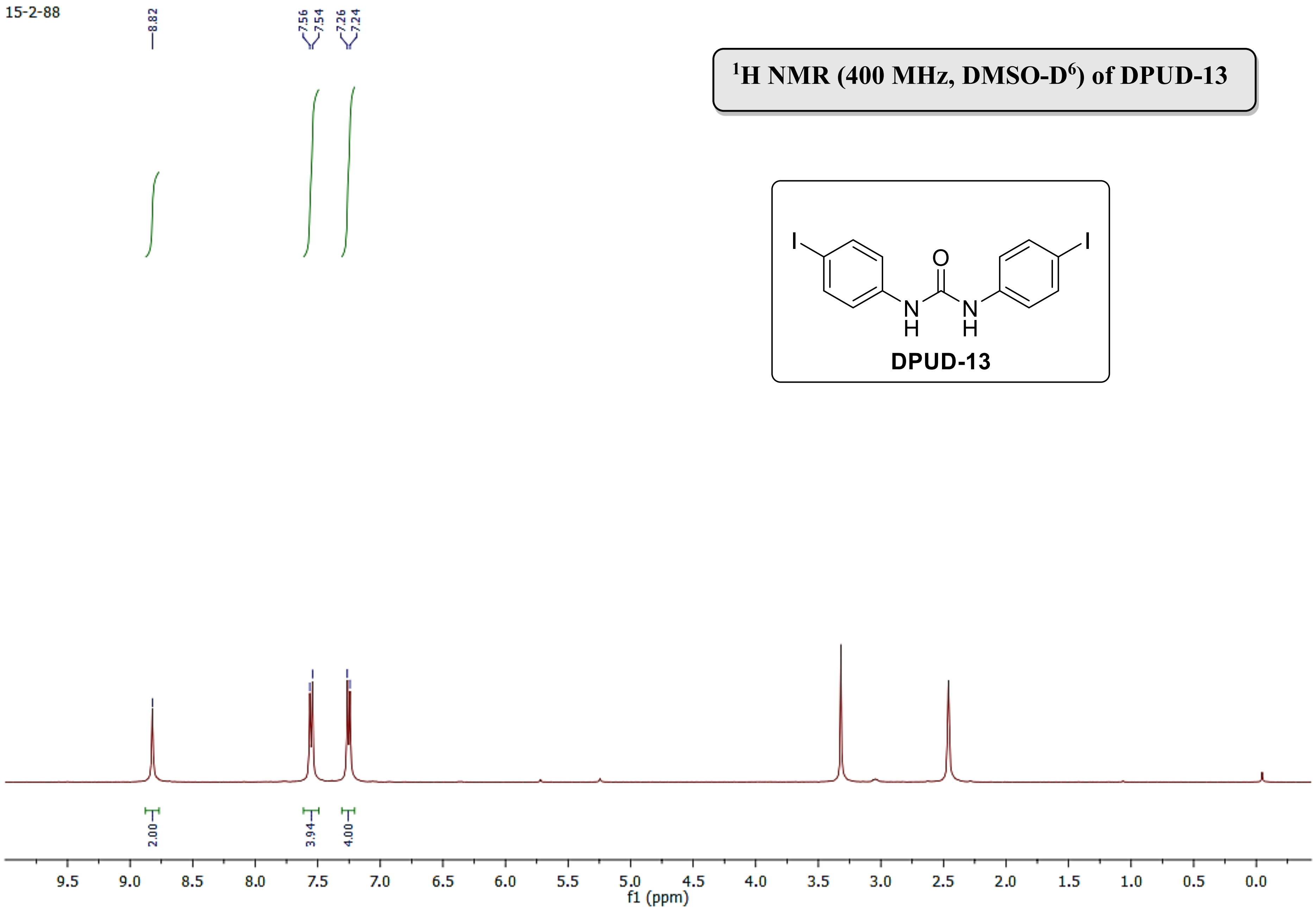

^{1}H NMR (400 MHz, DMSO-D[6]) of DPUD-14

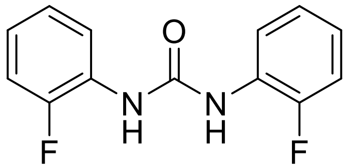

DPUD-14

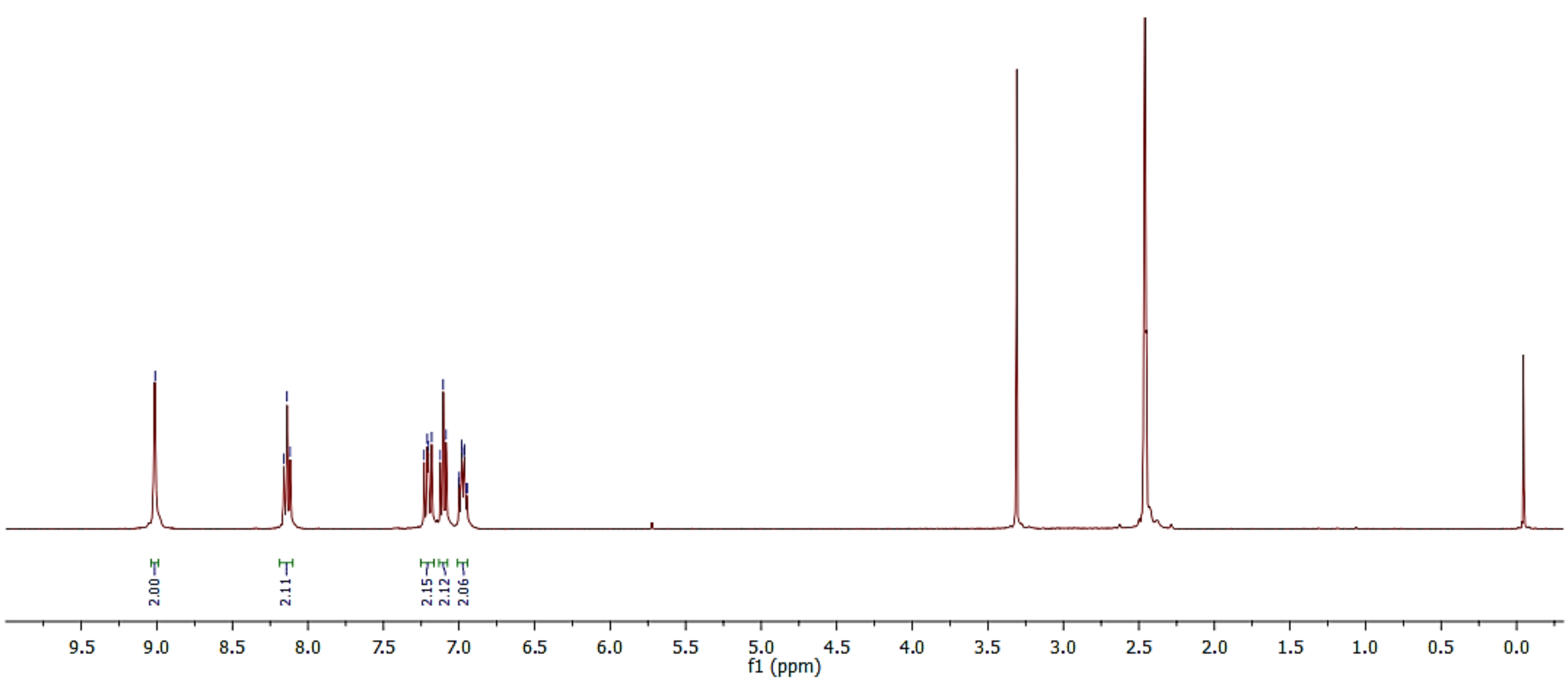

^{1}H NMR (400 MHz, DMSO-D^6) of DPUD-15

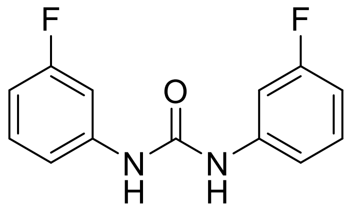

DPUD-15

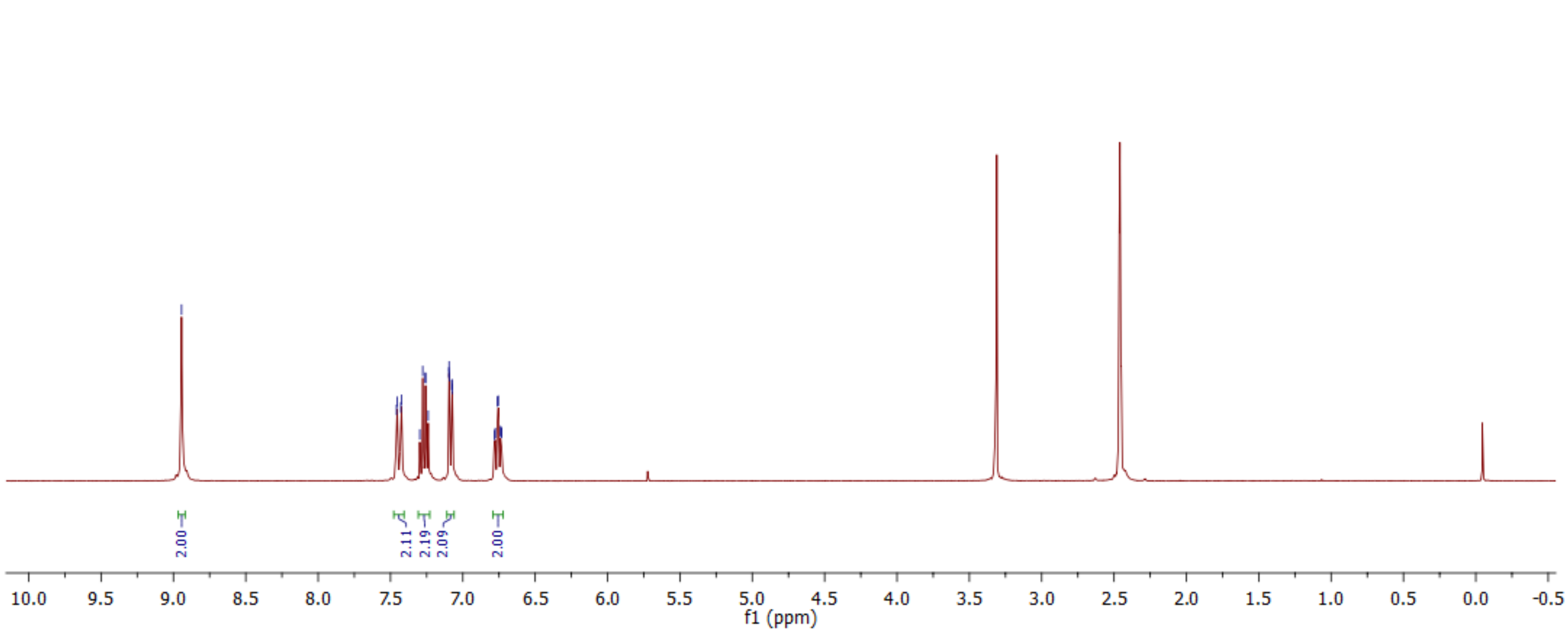

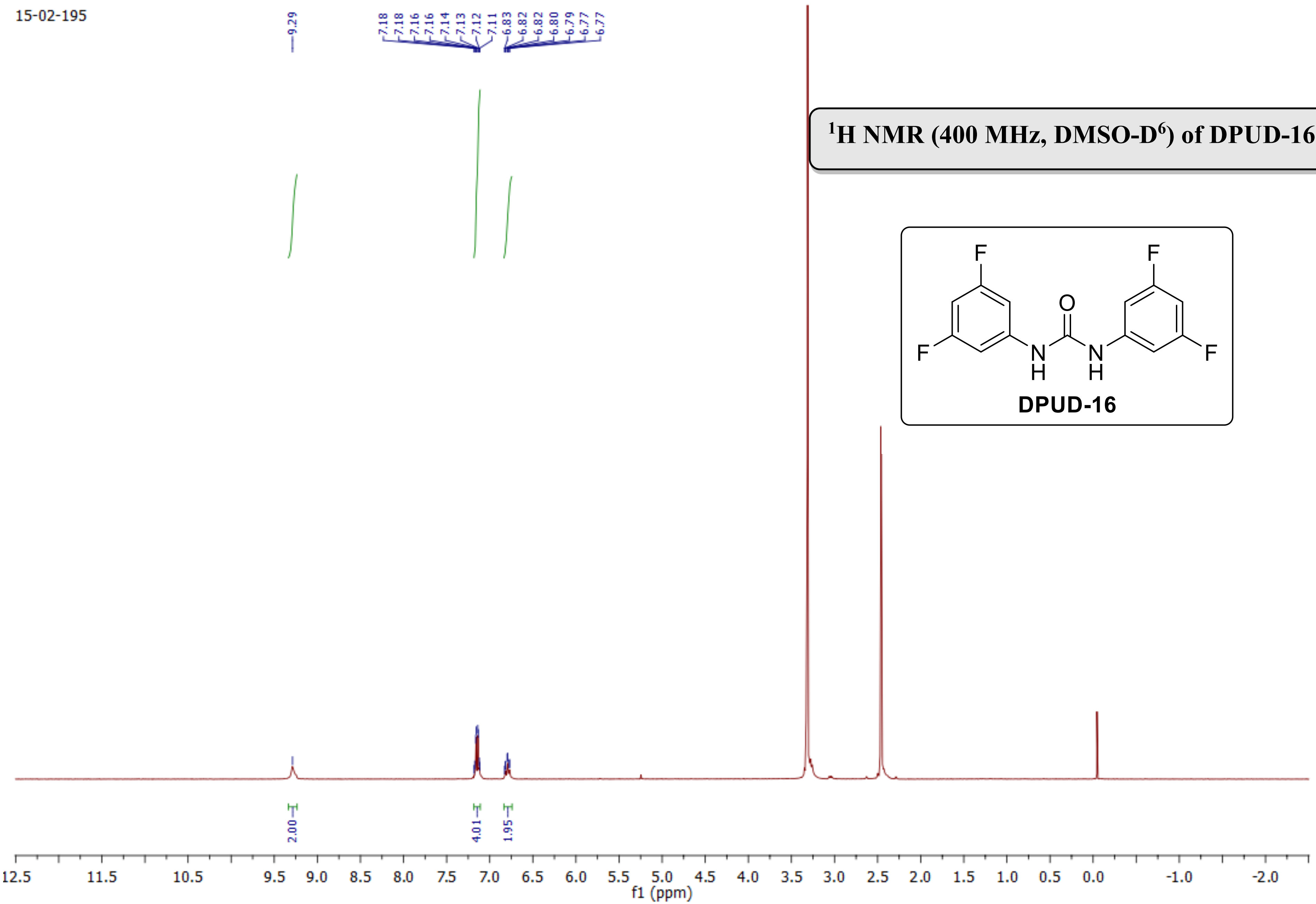

^{1}H NMR (400 MHz, DMSO-D^{6}) of DPUD-16

^{1}H NMR (400 MHz, DMSO-D[6]) of DPUD-17

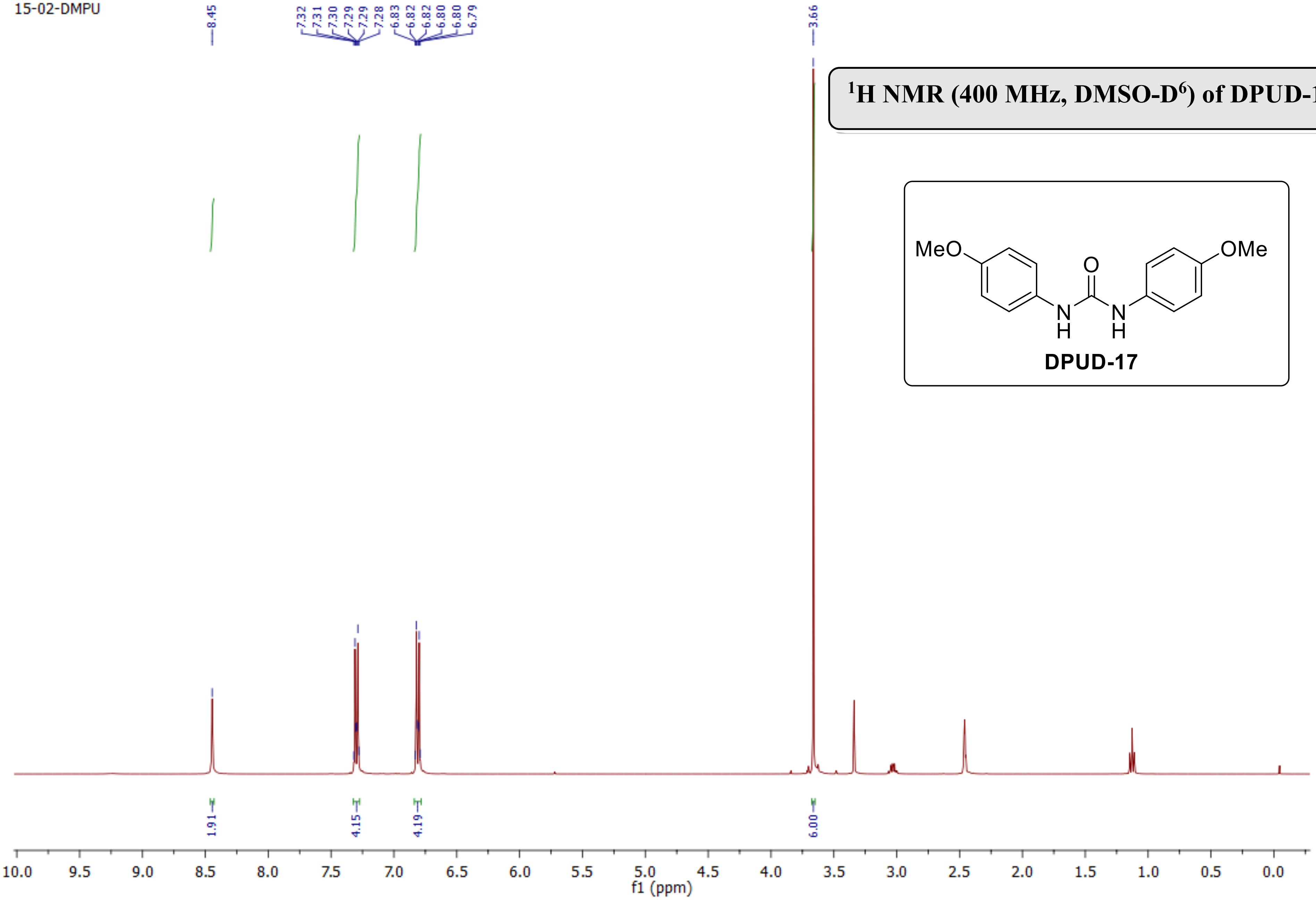

DPUD-17

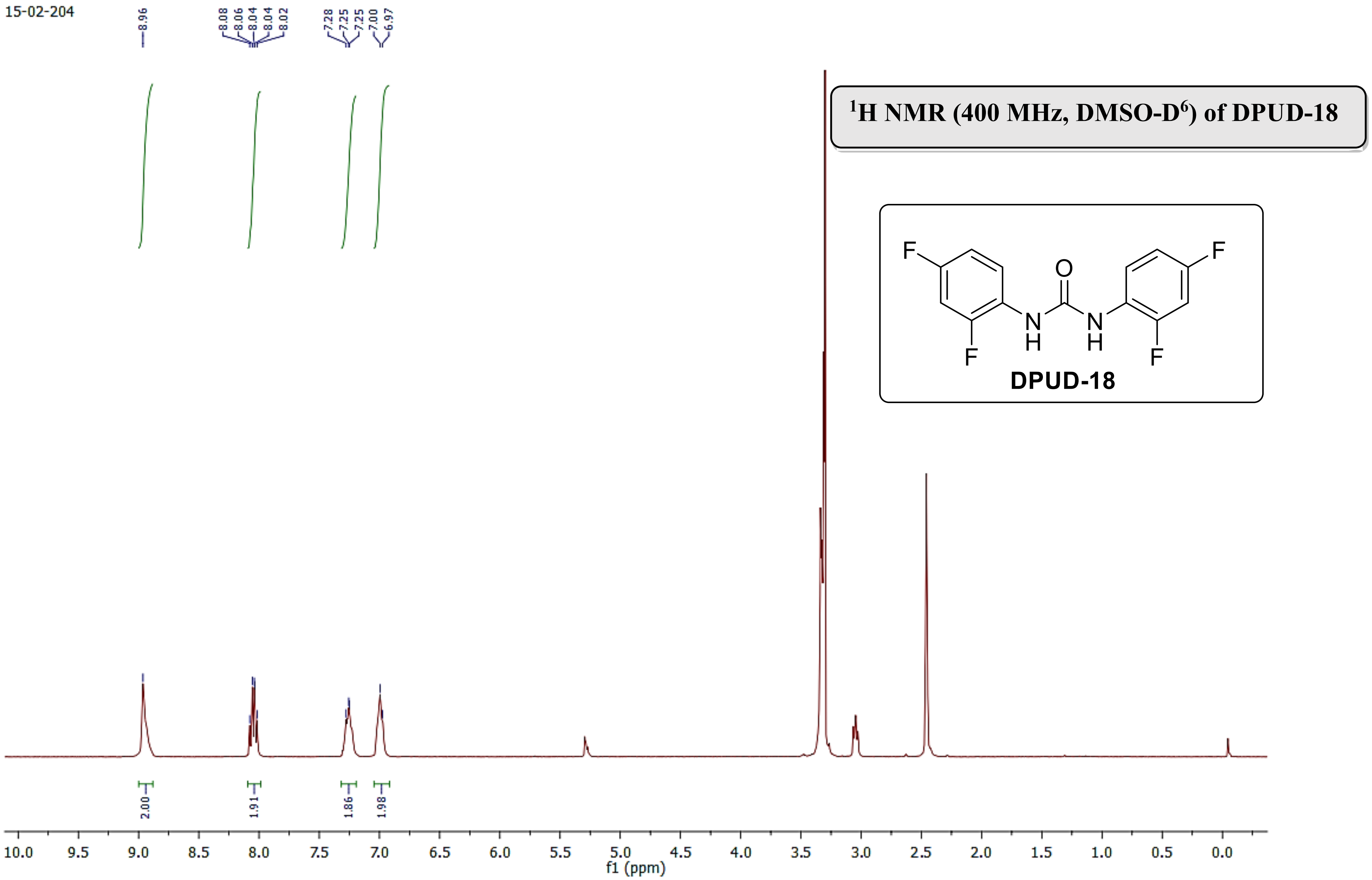
15-02-204
8.96
8.08
8.06
8.04
8.04
8.02
7.28
7.25
7.25
7.00
6.97
^{1}H NMR (400 MHz, DMSO-D^{6}) of DPUD-18
F
F
O
N
H
N
H
F
F
DPUD-18
2.00
1.91
1.86
1.98
10.0 9.5 9.0 8.5 8.0 7.5 7.0 6.5 6.0 5.5 5.0 4.5 4.0 3.5 3.0 2.5 2.0 1.5 1.0 0.5 0.0
f1 (ppm)

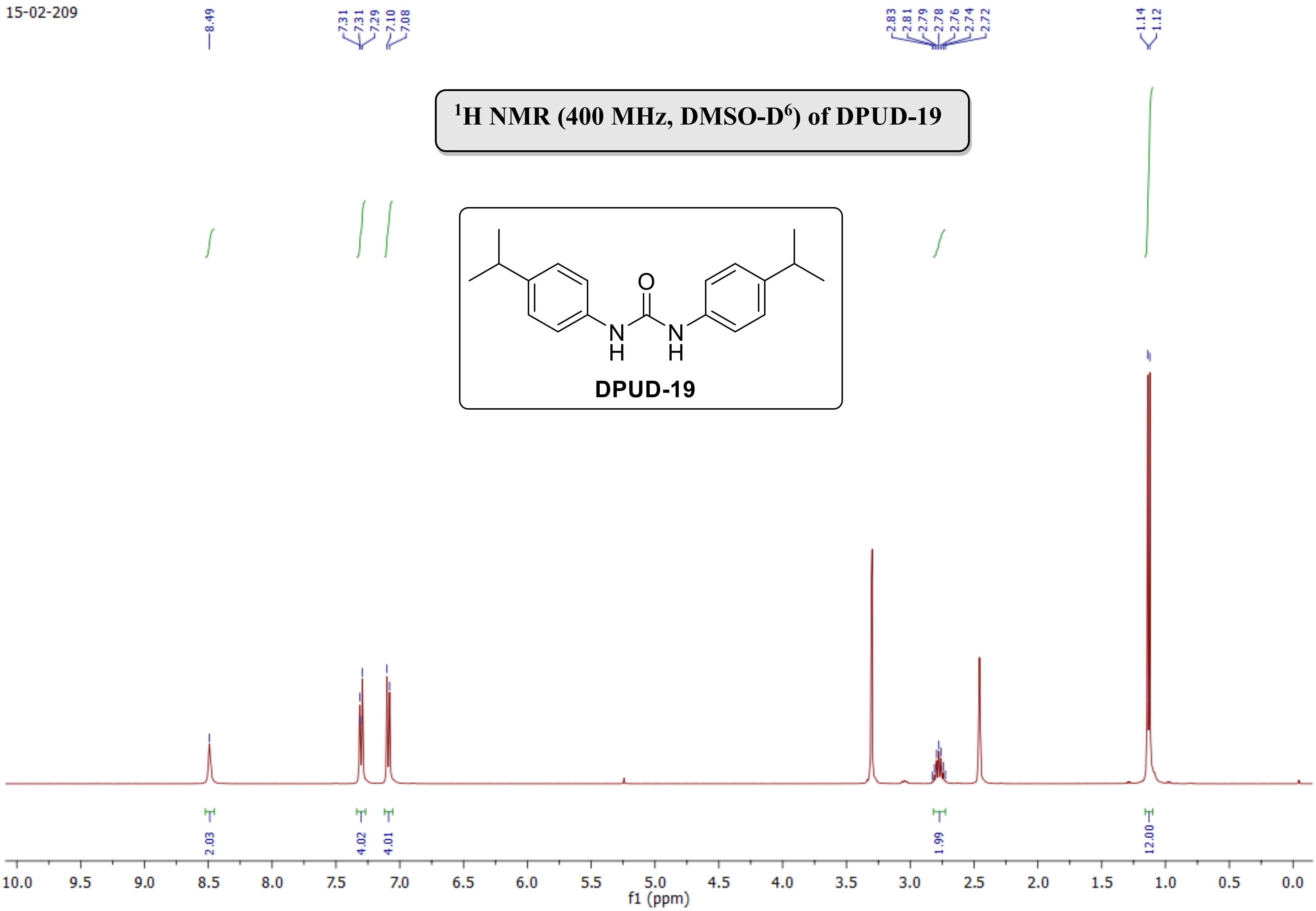
15-02-209
8.49
7.31
7.31
7.29
7.10
7.08
2.83
2.81
2.79
2.78
2.76
2.74
2.72
1.14
1.12
1H NMR (400 MHz, DMSO-D6) of DPUD-19
O
N
H
N
H
DPUD-19
2.03
4.02
4.01
1.99
12.00
10.0
9.5
9.0
8.5
8.0
7.5
7.0
6.5
6.0
5.5
5.0
4.5
4.0
3.5
3.0
2.5
2.0
1.5
1.0
0.5
0.0
f1 (ppm)

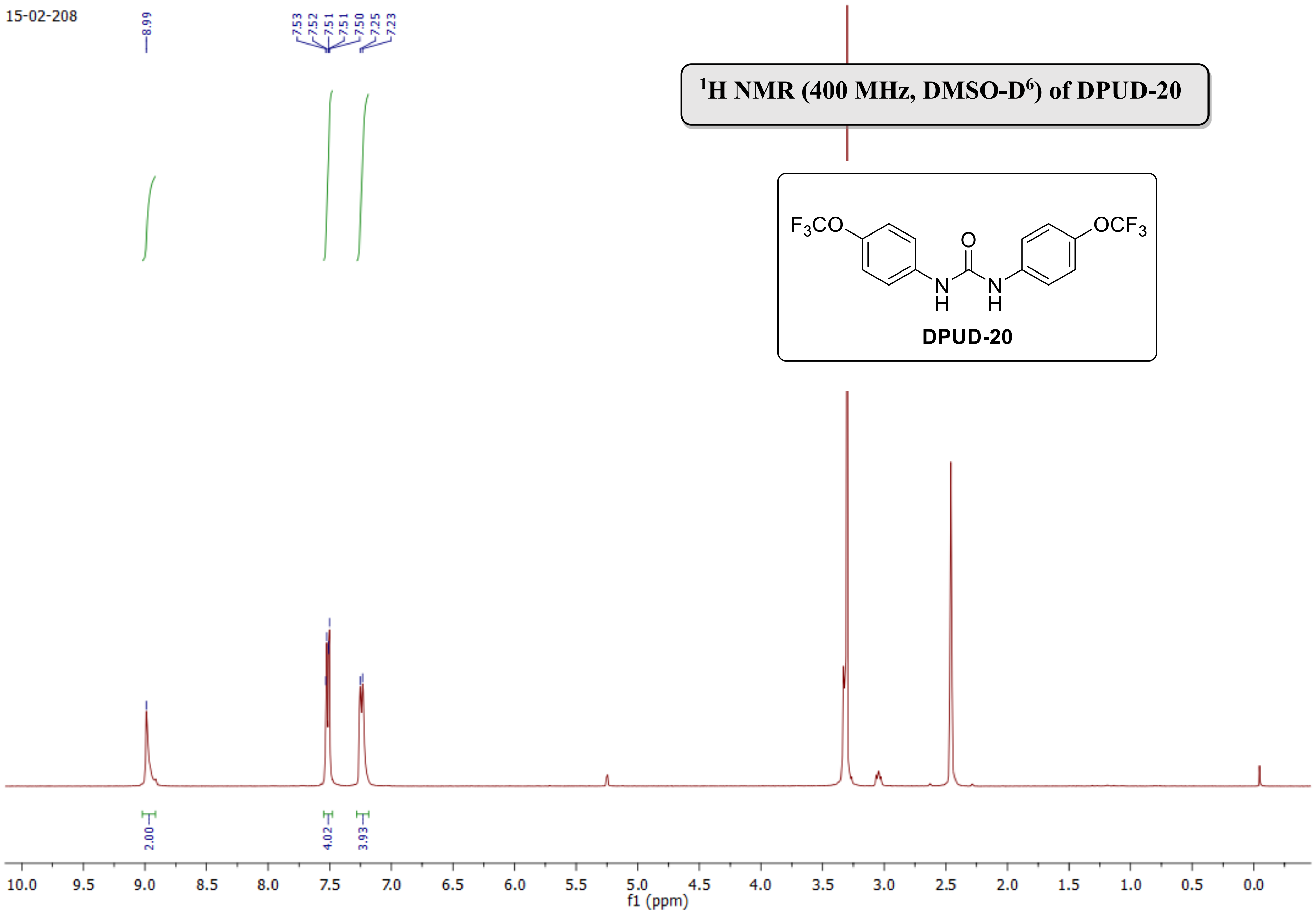
15-02-208
8.99
7.53
7.52
7.51
7.51
7.50
7.25
7.23
¹H NMR (400 MHz, DMSO-D⁶) of DPUD-20
F3CO
OCF3
O
N
H
N
H
DPUD-20
2.00
4.02
3.93
10.0
9.5
9.0
8.5
8.0
7.5
7.0
6.5
6.0
5.5
5.0
4.5
4.0
3.5
3.0
2.5
2.0
1.5
1.0
0.5
0.0
f1 (ppm)

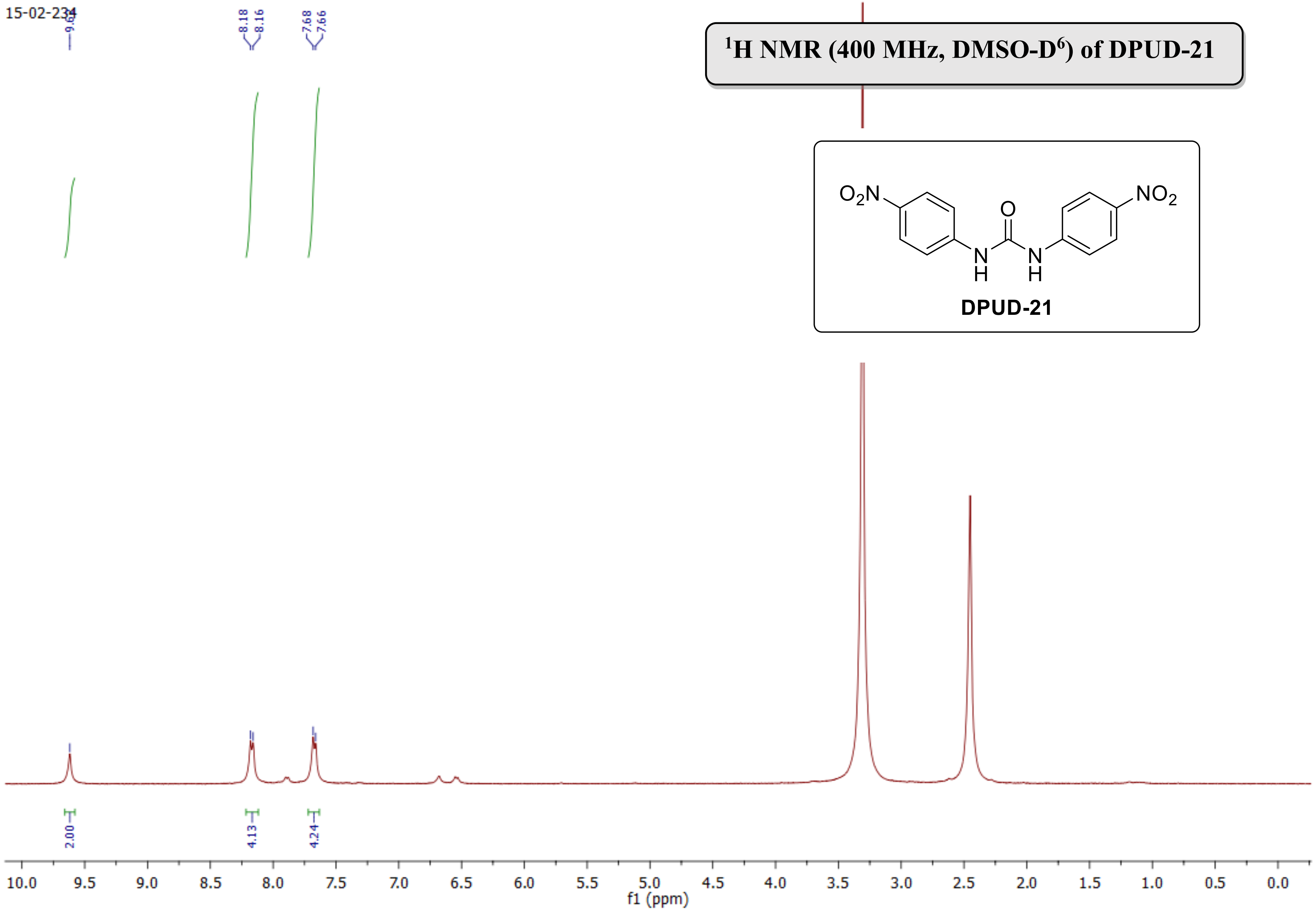
15-02-234
9.62
8.18
8.16
7.68
7.66
^{1}H NMR (400 MHz, DMSO-D^6) of DPUD-21
O_2N
NO_2
O
N H
N H
DPUD-21
2.00
4.13
4.24
10.0 9.5 9.0 8.5 8.0 7.5 7.0 6.5 6.0 5.5 5.0 4.5 4.0 3.5 3.0 2.5 2.0 1.5 1.0 0.5 0.0
f1 (ppm)

^{1}H NMR (400 MHz, DMSO-D^{6}) of DPUD-22

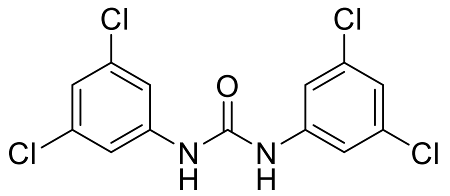

DPUD-22

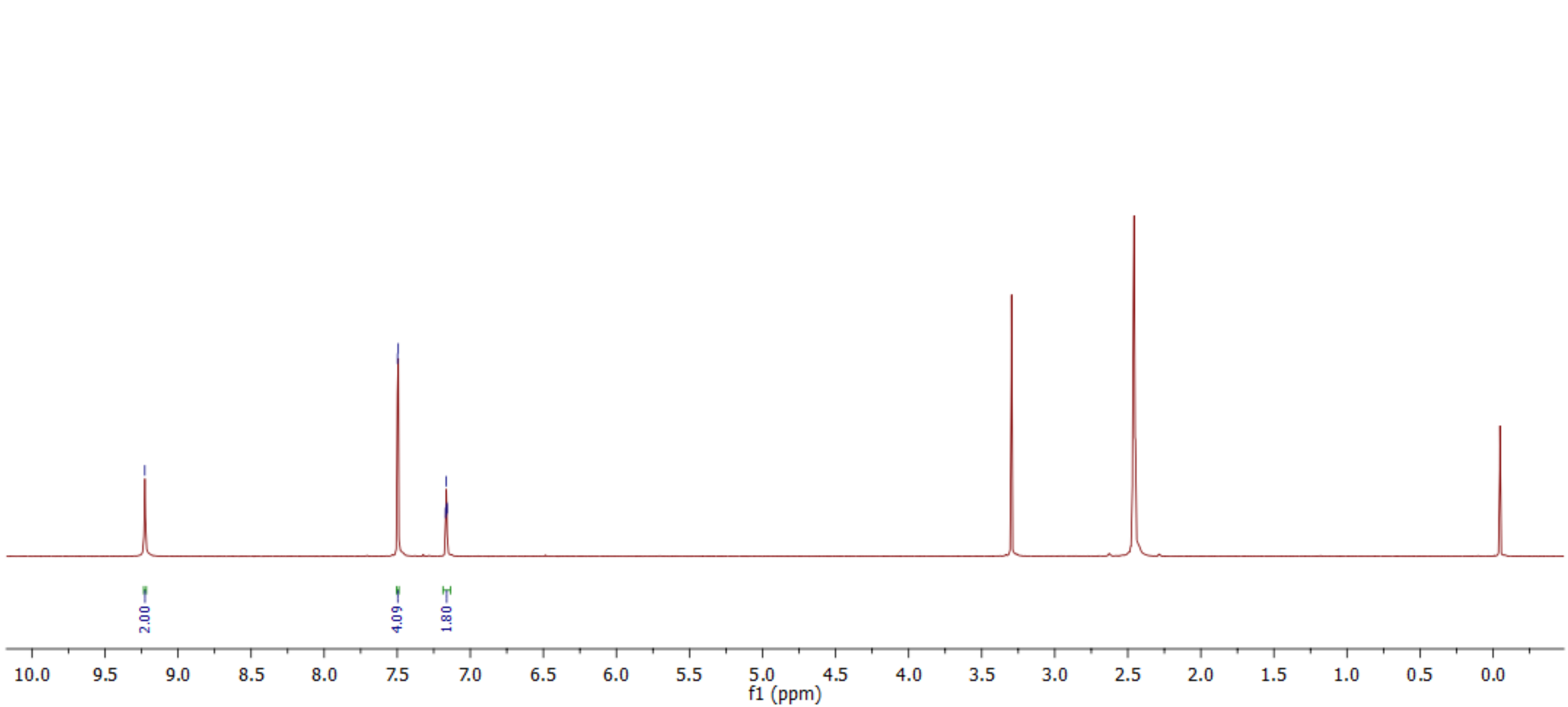

^{1}H NMR (400 MHz, DMSO-D[6]) of DPUD-23

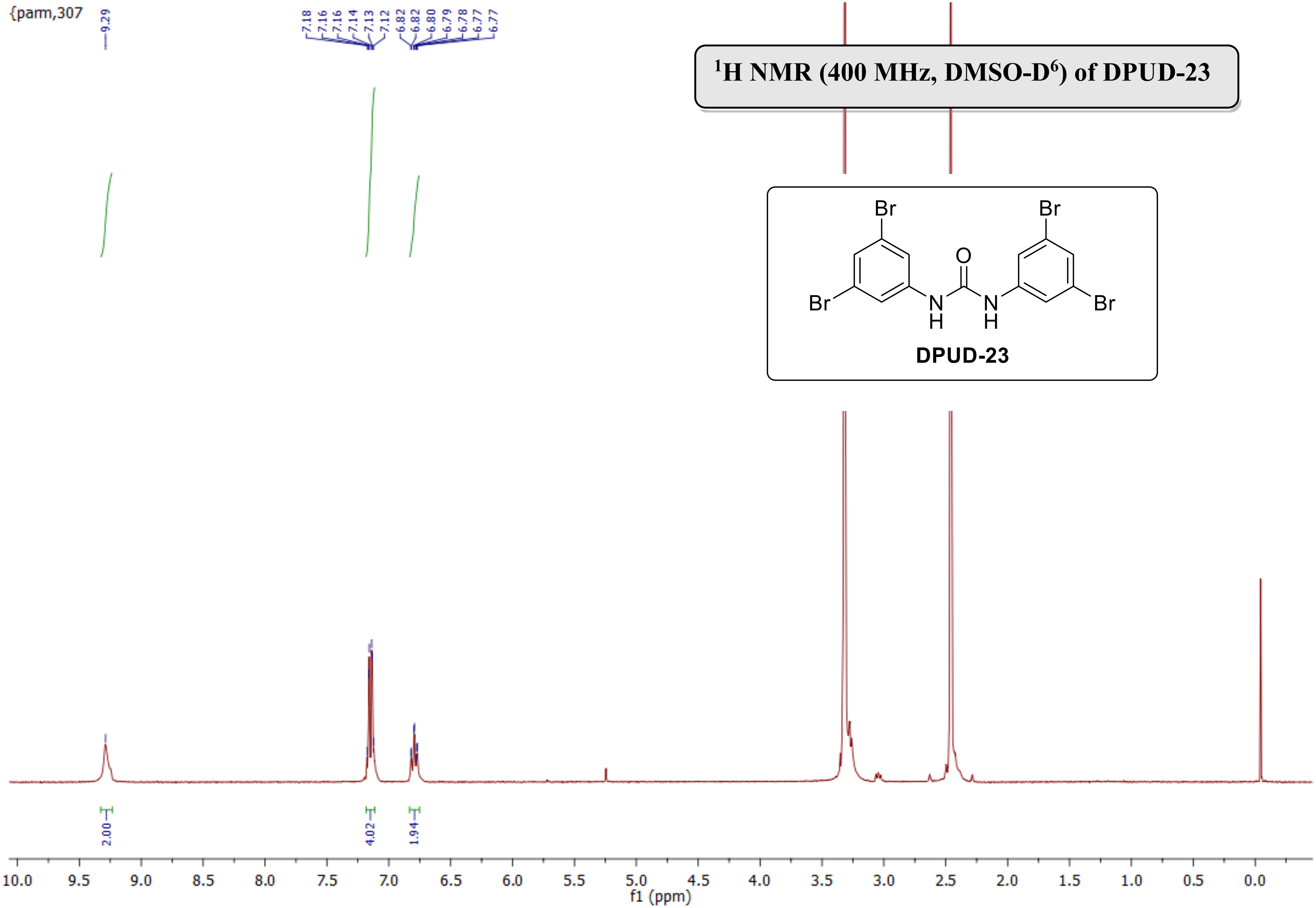

Supplement: S3 Table — (PDF) [file ppat.1011358.s010.pdf]
