# Supplementary material for: Identification of diphenylurea derivatives as novel endocytosis inhibitors that demonstrate broad-spectrum activity against SARS-CoV-2 and influenza A virus both in vitro and in vivo
Source: PLoS Pathog. 2023 May 1;19(5):e1011358. doi: 10.1371/journal.ppat.1011358 (PMC10174524; doi:10.1371/journal.ppat.1011358)
Supplement: S4 Table — (PDF) [file ppat.1011358.s011.pdf]

S4 Table

DPUD-1

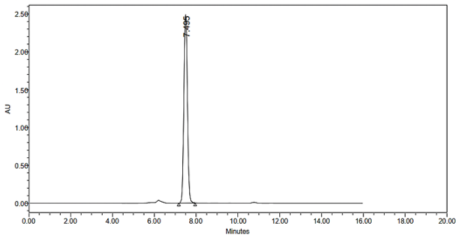

	RT	Area	% Area	Height
1	7.495	29083914	100.00	2.495210

DPUD-2

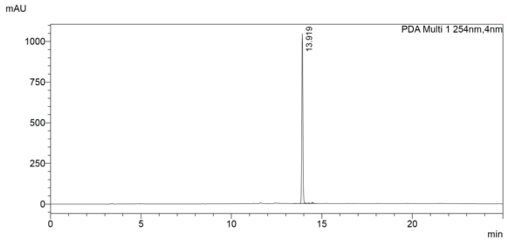

<Peak Table>

PDA Ch1 254nm

Peak#	Ret. Time	Area	Height	Conc.	Unit	Mark	Name
1	13.919	4284798	1045389	0.000		M	
Total		4284798	1045389				

DPUD-3

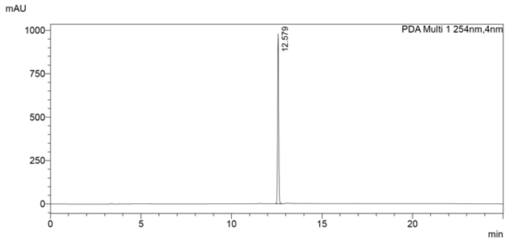

<Peak Table>

PDA Ch1 254nm

Peak#	Ret. Time	Area	Height	Conc.	Unit	Mark	Name
1	12.579	4002651	979113	0.000			
Total		4002651	979113				

DPUD-4

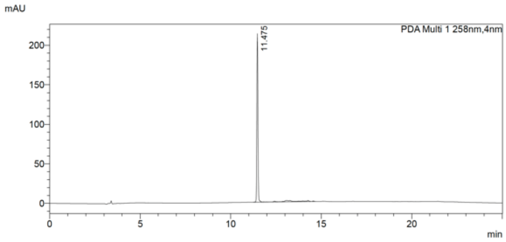

<Peak Table>

PDA Ch1 258nm

Peak#	Ret. Time	Area	Height	Conc.	Unit	Mark	Name
1	11.475	966539	213266	100.000		S	
Total		966539	213266				

DPUD-5

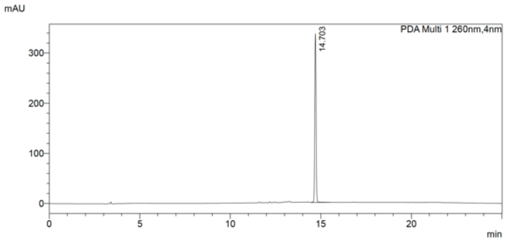

<Peak Table>

PDA Ch1 260nm

Peak#	Ret. Time	Area	Height	Conc.	Unit	Mark	Name
1	14.703	1641324	336722	100.000		S	
Total		1641324	336722				

DPUD-6

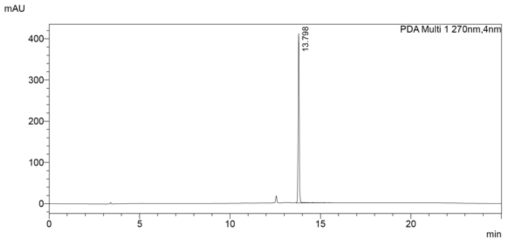

<Peak Table>

PDA Ch1 270nm

Peak#	Ret. Time	Area	Height	Conc.	Unit	Mark	Name
1	13.798	1794166	410569	0.000		S	
Total		1794166	410569				

DPUD-7

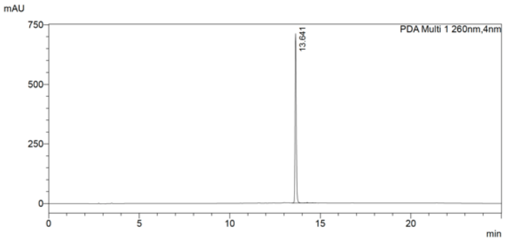

<Peak Table>

PDA Ch1 260nm

Peak#	Ret. Time	Area	Height	Conc.	Unit	Mark	Name
1	13.641	3262493	711158	100.000		S	
Total		3262493	711158				

DPUD-8

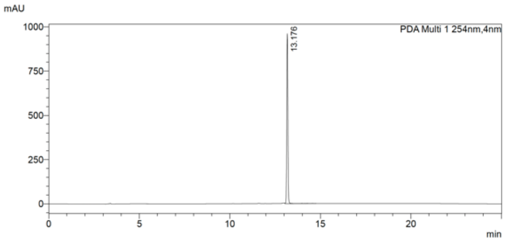

<Peak Table>

PDA Ch1 254nm

Peak#	Ret. Time	Area	Height	Conc.	Unit	Mark	Name
1	13.176	3973047	959786	0.000		S	
Total		3973047	959786				

DPUD-9

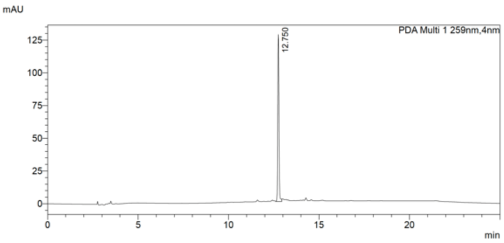

<Peak Table>
PDA Ch1 259nm

Peak#	Ret. Time	Area	Height	Conc.	Unit	Mark	Name
1	12.750	556538	127409	0.000			
Total		556538	127409				

DPUD-10

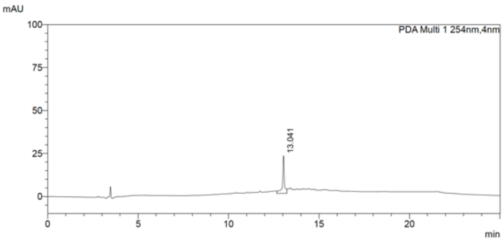

<Peak Table>
PDA Ch1 254nm

Peak#	Ret. Time	Area	Height	Conc.	Unit	Mark	Name
1	13.041	152921	21951	100.000			
Total		152921	21951				

DPUD-11

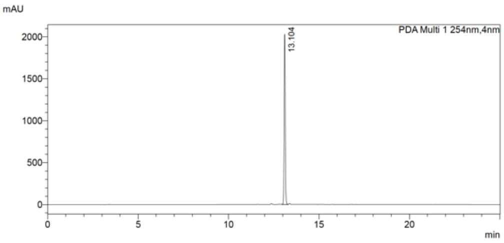

<Peak Table>
PDA Ch1 254nm

Peak#	Ret. Time	Area	Height	Conc.	Unit	Mark	Name
1	13.104	8755742	2029906	0.000			
Total		8755742	2029906				

DPUD-12

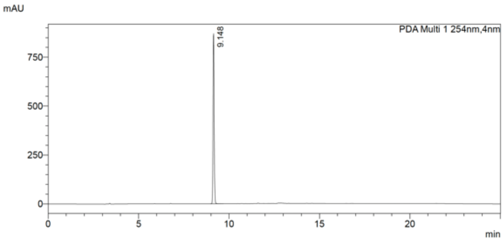

<Peak Table>
PDA Ch1 254nm

Peak#	Ret. Time	Area	Height	Conc.	Unit	Mark	Name
1	9.148	3692457	869786	0.000			
Total		3692457	869786				

DPUD-13

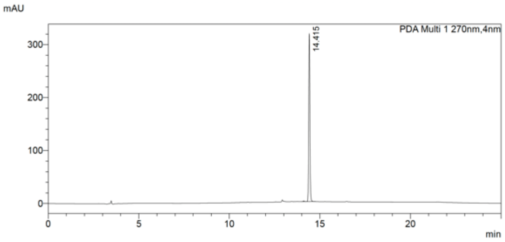

<Peak Table>
PDA Ch1 270nm

Peak#	Ret. Time	Area	Height	Conc.	Unit	Mark	Name
1	14.415	1440242	317140	0.000		M	
Total		1440242	317140				

DPUD-14

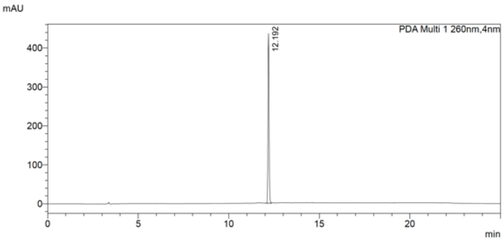

<Peak Table>
PDA Ch1 260nm

Peak#	Ret. Time	Area	Height	Conc.	Unit	Mark	Name
1	12.192	1755297	435441	100.000			
Total		1755297	435441				

DPUD-15

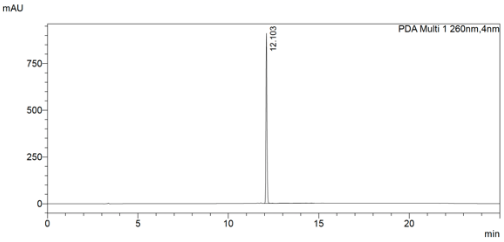

<Peak Table>
PDA Ch1 260nm

Peak#	Ret. Time	Area	Height	Conc.	Unit	Mark	Name
1	12.103	3668194	909698	0.000		V	
Total		3668194	909698				

DPUD-16

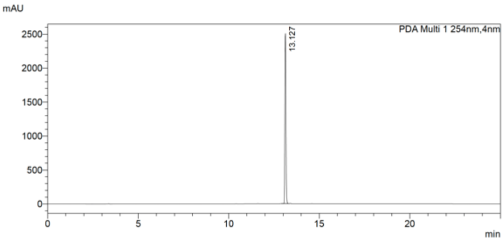

<Peak Table>
PDA Ch1 254nm

Peak#	Ret. Time	Area	Height	Conc.	Unit	Mark	Name
1	13.127	10067287	2505217	0.000		M	
Total		10067287	2505217				

DPUD17

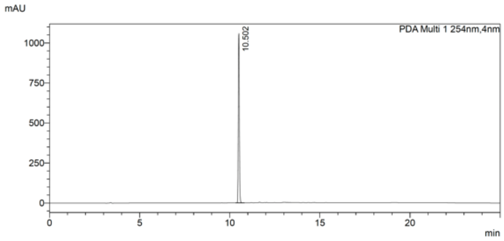

<Peak Table>

PDA Ch1 254nm

Peak#	Ret. Time	Area	Height	Conc.	Unit	Mark	Name
1	10.502	4300057	1058158	0.000			
Total		4300057	1058158				

DPUD-18

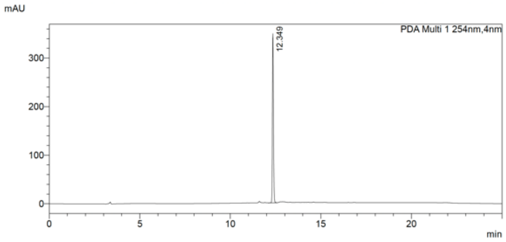

<Peak Table>

PDA Ch1 254nm

Peak#	Ret. Time	Area	Height	Conc.	Unit	Mark	Name
1	12.349	1396647	348435	0.000			
Total		1396647	348435				

DPUD-19

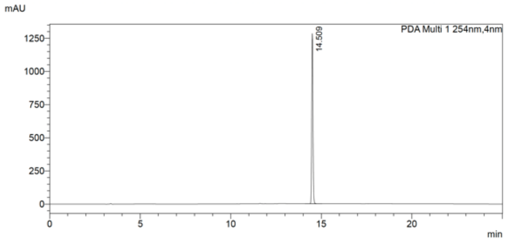

<Peak Table>

PDA Ch1 254nm

Peak#	Ret. Time	Area	Height	Conc.	Unit	Mark	Name
1	14.509	5858255	1282693	0.000		M	
Total		5858255	1282693				

DPUD-20

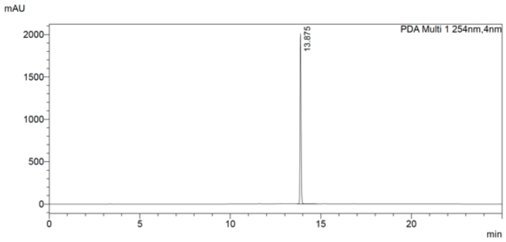

<Peak Table>

PDA Ch1 254nm

Peak#	Ret. Time	Area	Height	Conc.	Unit	Mark	Name
1	13.875	7912746	2008682	0.000			
Total		7912746	2008682				

DPUD-21

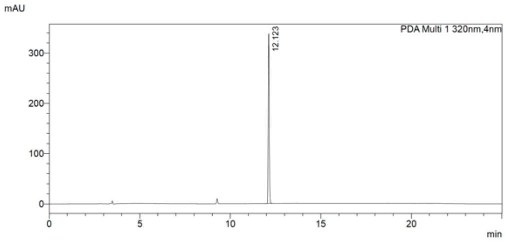

<Peak Table>

PDA Ch1 320nm

Peak#	Ret. Time	Area	Height	Conc.	Unit	Mark	Name
1	12.123	1283486	337967	100.000		M	
Total		1283486	337967				

DPUD-22

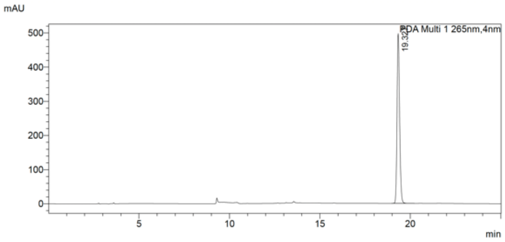

<Peak Table>

PDA Ch1 265nm

Peak#	Ret. Time	Area	Height	Conc.	Unit	Mark	Name
1	19.327	4898208	496966	100.000		S	
Total		4898208	496966				

DPUD-23

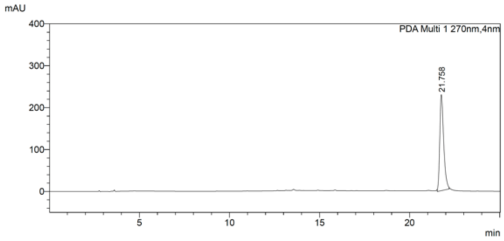

<Peak Table>

PDA Ch1 270nm

Peak#	Ret. Time	Area	Height	Conc.	Unit	Mark	Name
1	21.758	3481490	229554	0.000		M	
Total		3481490	229554				
